# Coordinated response of the *Desulfovibrio desulfuricans* 27774 transcriptome to nitrate, nitrite and nitric oxide

**DOI:** 10.1038/s41598-017-16403-4

**Published:** 2017-11-24

**Authors:** Ian T. Cadby, Matthew Faulkner, Jeanne Cheneby, Justine Long, Jacques van Helden, Alain Dolla, Jeffrey A. Cole

**Affiliations:** 1 School of Biosciences, University of Birmingham, Birmingham, B15 2TT UK; 2 Aix Marseille Univ, INSERM, TAGC, UMR_S 1090, 163, Avenue de Luminy, 13288 Marseille, France; 3 Aix Marseille Univ, CNRS, LCB, Marseille, France; 4 Present Address: The Institute of Integrative Biology, Bioscience building, University of Liverpool, Liverpool, Merseyside L69 7ZB UK

## Abstract

The sulfate reducing bacterium *Desulfovibrio desulfuricans* inhabits both the human gut and external environments. It can reduce nitrate and nitrite as alternative electron acceptors to sulfate to support growth. Like other sulphate reducing bacteria, it can also protect itself against nitrosative stress caused by NO generated when nitrite accumulates. By combining *in vitro* experiments with bioinformatic and RNA-seq data, metabolic responses to nitrate or NO and how nitrate and nitrite reduction are coordinated with the response to nitrosative stress were revealed. Although nitrate and nitrite reduction are tightly regulated in response to substrate availability, the global responses to nitrate or NO were largely regulated independently. Multiple NADH dehydrogenases, transcription factors of unknown function and genes for iron uptake were differentially expressed in response to electron acceptor availability or nitrosative stress. Amongst many fascinating problems for future research, the data revealed a YtfE orthologue, Ddes_1165, that is implicated in the repair of nitrosative damage. The combined data suggest that three transcription factors coordinate this regulation in which NrfS-NrfR coordinates nitrate and nitrite reduction to minimize toxicity due to nitrite accumulation, HcpR1 serves a global role in regulating the response to nitrate, and HcpR2 regulates the response to nitrosative stress.

## Introduction

Many strains of sulfate reducing bacteria such as *Desulfovibrio vulgaris* that have been isolated from natural environments outside warm blooded animals lack genes for nitrate reduction, but with few exceptions, they are able to reduce nitrite to ammonia^1–8^. Nitrite reduction is catalysed by the periplasmic NrfHA nitrite reductase^4,9–11^. Amongst the exceptions is *D. alaskensis* strain G20, which lacks the *nrfHA* genes and is extremely sensitive to nitrite toxicity^2,7^. Although originally there were conflicting reports concerning whether nitrite reduction is constitutive or inducible, it is now well established that *nrfHA* is expressed in the absence of nitrate or nitrite at a level that is sufficient to provide protection against the toxicity of nitrite produced, for example, by other bacteria that share their environment^2–4,12,13^. This background level increases significantly in the presence of nitrite^11,14^. Nitrite induction in *D. vulgaris* was recently shown to be dependent upon nitrite activation of a σ^54^-dependent two component regulatory system, NrfS-NrfR^11,15^. Boinformatic analysis revealed that the same is likely to be true for *D. desulfuricans* ^11^.

Unlike *D. vulgaris*, *D. desulfuricans* is able to use nitrate as an alternative to sulfate as the terminal electron acceptor to support growth^2,12,13,16^. Nitrate reduction is catalysed by a periplasmic nitrate reductase encoded in the *napCMADGH* genes^14^. Expression of the nitrate reductase operon in the most studied type strain, *D. desulfuricans* 27774, is induced during growth in the presence of nitrate but repressed by sulfate, even in the presence of nitrate^12,14^. This implies that nitrate reduction is regulated by at least two mechanisms, one for nitrate induction, the other for sulfate repression. We and others have noted the presence of potential binding sites for the NrfS-NrfR two-component regulatory system in the *nap* regulatory region^11,16^. No other potential binding sites for NrfR were found in the *D. desulfuricans* genome^17^.

Nitric oxide is an obligate intermediate during denitrification, but bacteria that reduce nitrate to ammonia also generate small quantities of NO, which in turn activates a protective nitrosative stress response. The main source of NO for free-living bacteria is nitrite generated either as a product of their own metabolism, or by other bacteria that share their environment. Bacteria that live in the bodies of warm-blooded animals are also exposed to NO generated from arginine as part of the host defence mechanisms. Homologues of genes that protect bacteria from nitrosative stress can be identified in the genomes of sulfate reducing bacteria^15,16,18–21^. They include transcription factors such as HcpR and genes that they regulate such as *hcp* encoding the hybrid cluster protein, Hcp. We recently showed that Hcp in enteric bacteria is a high affinity nitric oxide reductase that protects cytoplasmic proteins from nitrosative damage by NO generated as a side product of nitrite reduction to ammonia^22^. There is evidence that the same is true for Hcp in sulfate reducing bacteria^23^.

Sulfate reducing bacteria vary in that while some include single genes encoding Hcp and its transcription regulator, HcpR, others encode two or even more copies of these genes. For example, in *D. vulgaris* there are two copies of the *hcp* gene and also two copies of the gene for the flavodiiron protein ROO^24^. Both have been implicated in the protection against nitrosative stress^6,24–26^. Synthesis of Hcp2 is strongly induced by exposure to NO, and deletion of *hcp2* results in increased sensitivity to NO^27^.

Unlike *D. vulgaris*, strains of *D. desulfuricans* and its close relatives are commonly found in the bodies of warm blooded animals and can readily be isolated from human feces^28,29^. Two of the 5 transcription factors of the Crp-FNR family encoded in the *D. desulfuricans* 27774 genome, HcpR1 and HcpR2, are both predicted to regulate the response to nitrosative stress. Correlations were noted between the ability of sulfate reducing bacteria to reduce nitrate, their ability to survive in the human body, and the presence of genes for both HcpR1 and HcpR2^30^. A fascinating result from the previous study was that although NO induced expression of the *hcp* and *hcpR1* genes, HcpR2 was shown to be the transcription factor that regulates Hcp synthesis, but *hcpR2* transcription is not induced by NO.

HcpR1 binds to a perfect inverted repeat sequence, 5′-TGTGA-N6-TCACA, which is identical to the consensus binding site for the c-AMP receptor protein, CRP, in *Escherichia coli* ^14,19,30^. Binding of HcpR1 to this site regulates *hcpR1* transcription^19,30^. There are also inverted repeat sequences predicted to be the binding sites for HcpR1 immediately upstream of the *napC* promoter, suggesting that HcpR1 might regulate nitrate reduction as well as its own synthesis. This suggests that HcpR1 regulates the synthesis of enzymes that enable *D. desulfuricans* both to reduce nitrate and protect itself from toxic products generated during nitrate and nitrite reduction. The combined data from these earlier studies indicate that multiple transcription factors are likely to be involved in the regulation of nitrate, nitrite and NO reduction. They suggest that while HcpR1 serves a more global role, NrfS-NrfR is a dedicated system that senses the presence of nitrite or nitrate and coordinates nitrate and nitrite reduction to minimize toxicity due to nitrite accumulation. The first aims of the current work were to determine how expression of nitrate and nitrite reductase genes are regulated in response to substrate availability, and demonstrate that HcpR1 binds to the regulatory region of the *nap* gene cluster.

Although attempts by us and other laboratories to isolated specific mutants of *D. desulfuricans* have been unsuccessful, global transcriptome analysis has been used successfully to analyse how other sulphate reducing bacteria respond to oxidative stress, heat shock and electron acceptor availability^31–34^. We have therefore used RNA-seq based transcriptome analysis to determine the global response of *D. desulfuricans* 27774 to nitrate or NO.

## Results

### Conserved nap gene clusters and their regulatory motifs in Desulfovibrio species

Many genomes of sulfate reducing bacteria have been sequenced since the original report of the structure and sequence of the *napCMADGH* gene cluster of *D. desulfuricans* 27774^14^. This NapM sequence was used to identify similar *nap* gene clusters in other bacteria. These blast searches revealed additional *Desulfovibrio* species with similar clusters, despite their widely different genome sizes and GC contents. Similar clusters were found in sulfite reducing *Bilophila* species^35^. Some of the bacteria with similar NapM sequences lack the *napC* gene, but retain the *napMADGH* sequences (Supplementary Table S1). The *napM* gene is present in all *nap* gene clusters found in sulfate reducing bacteria.

We previously showed that HcpR1 binds to a DNA fragment containing the sequence immediately upstream of the *D. desulfuricans* 27774 *hcpR1* gene, suggesting that HcpR1 regulates its own synthesis^30^. We also reported the presence of three similar sequences in the *napC* regulatory region of this strain^14^. Potential HcpR1 binding sites were found in the regulatory regions of eight out of ten scanned *napM* sequences, although the location of these sites varies in different bacteria (Supplementary Table S1). The position of the sites in *Desulfovibrio* species is consistent with HcpR1 acting as a class 3 transcription activator, a mechanism in which the binding of transcription factors to two sites is required for optimal activation of gene expression^36^. In contrast, the predicted HcpR1 site in *Deferribacter* overlaps the translation start codon, consistent with a repression function. Binding sites similar to those bound by the two-component regulatory system NrfS-NrfR were also located upstream of *nap* gene clusters in these various sulphate reducing bacteria (Supplementary Table S1).

### Binding of purified recombinant HcpR1 to inverted repeat sequences in the regulatory region of the nap gene cluster

**Figure 1:**
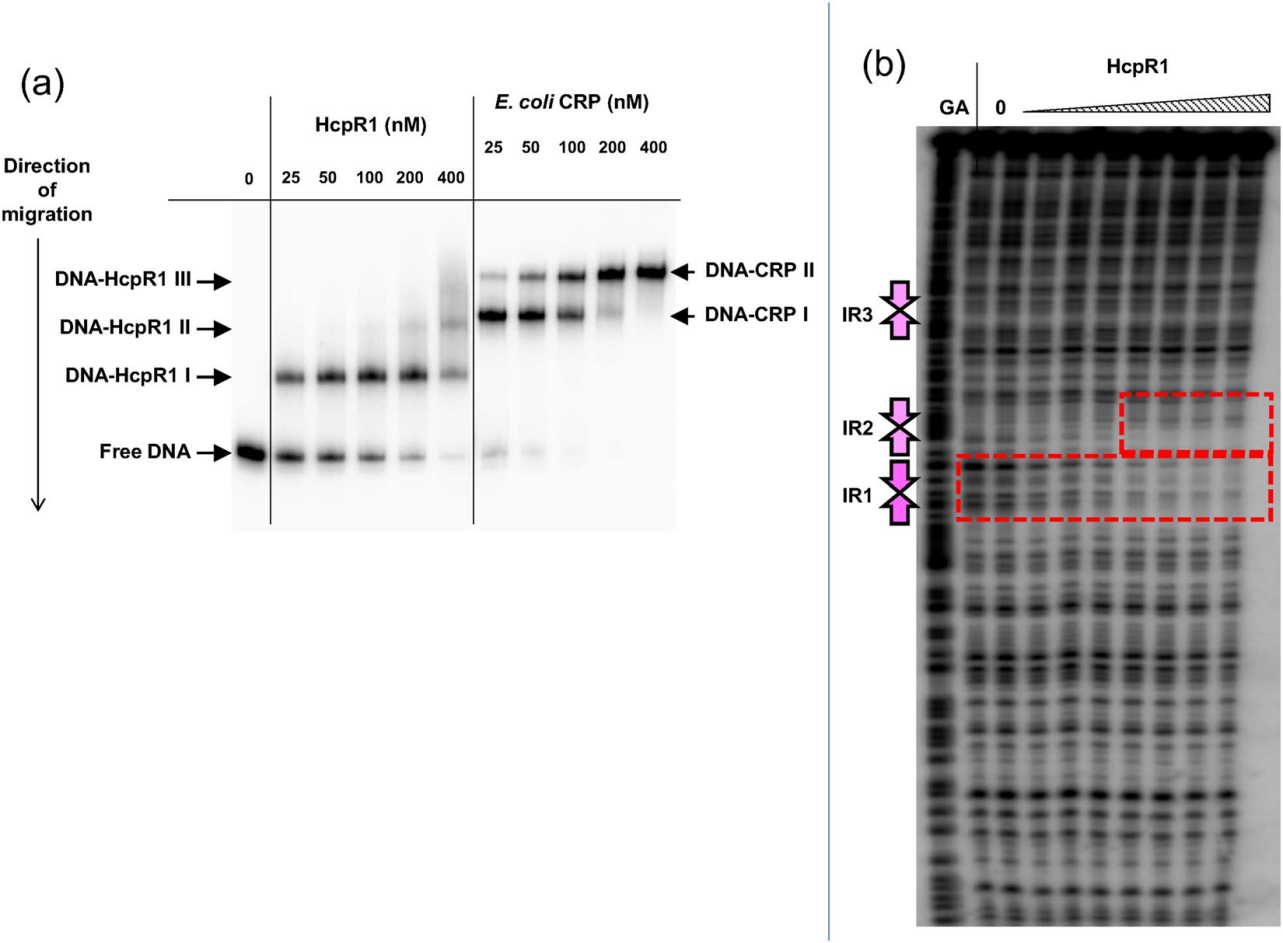
Comparison between the DNA binding affinities of HcpR1 and CRP for the *nap* promoter fragment and demonstration of binding to the p*nap* DNA fragment by DNaseI footprinting assays. (**a**) DNA binding affinities were assessed by EMSA. ^32^P-labelled *nap* promoter DNA fragment was incubated with increasing concentrations of HcpR1 alone, or CRP protein in the presence of 200 µM cAMP and then resolved by non-denaturing PAGE. Herring sperm DNA was also included in the incubation mixtures to act as non-specific competitor DNA. Free DNA, DNA-HcpR1 and DNA-CRP complexes are marked with arrows. (**b**) ^32^P-end-labelled *nap* promoter DNA fragment was incubated with increasing concentrations of HcpR1 protein and digested with DNaseI. Digest mixtures were then resolved by denaturing electrophoresis on urea acrylamide gels. Sequences were identified by including Maxam-Gilbert sequencing reactions (GA) on gels. Protected regions are marked by boxes and the positions of the three IR sequences are marked with pink arrows. Samples in tracks from left to right were incubated with 0, 5, 10, 20, 40, 80, 160, 360 and 720 nM HcpR1.

To confirm that HcpR1 binds to the inverted repeat sequences, different concentrations of purified HcpR1 were incubated with a ^32^P end-labelled DNA fragment covering the *napC* promoter and regulatory region. Protein-DNA complexes were separated from unbound DNA by non-denaturing PAGE and visualized by autoradiography (Fig. 1(a)). A single high affinity complex was detected even at the lowest concentration of HcpR1 protein. At the highest concentration, a second band due to a low affinity complex was detected. For comparison, the *napC* upstream fragment was also incubated with increasing concentrations of *E. coli* CRP plus 200 μM c-AMP. In contrast to HcpR1 binding, two band shifts were readily detected even at relatively low protein concentrations. Thus although CRP binds with relatively high affinity to two sites in the *napC* regulatory region, HcpR1 binds only one site. The concentrations of HcpR1 used in these experiments were in the same range as both the estimated concentrations of other transcription factors *in vivo* and the concentrations of transcription factors used in previous *in vitro* experiments. We therefore assume that the observed binding of HcpR1 to promoter DNA is physiologically relevant^37,38^.

DNA footprinting was used to confirm that HcpR1 binds specifically to the site identified from the bioinformatics analysis (Fig. 1(b)). The high affinity DNA-binding site marked with a red box around all tracks containing HcpR1 corresponds to IR1, the inverted repeat sequence closest to the transcription start site. At higher HcpR1 concentrations, the second binding site IR2 marked with the upper red box was protected.

### Effect of electron acceptor during growth on expression at the napC and nrfA promoters

**Figure 2:**
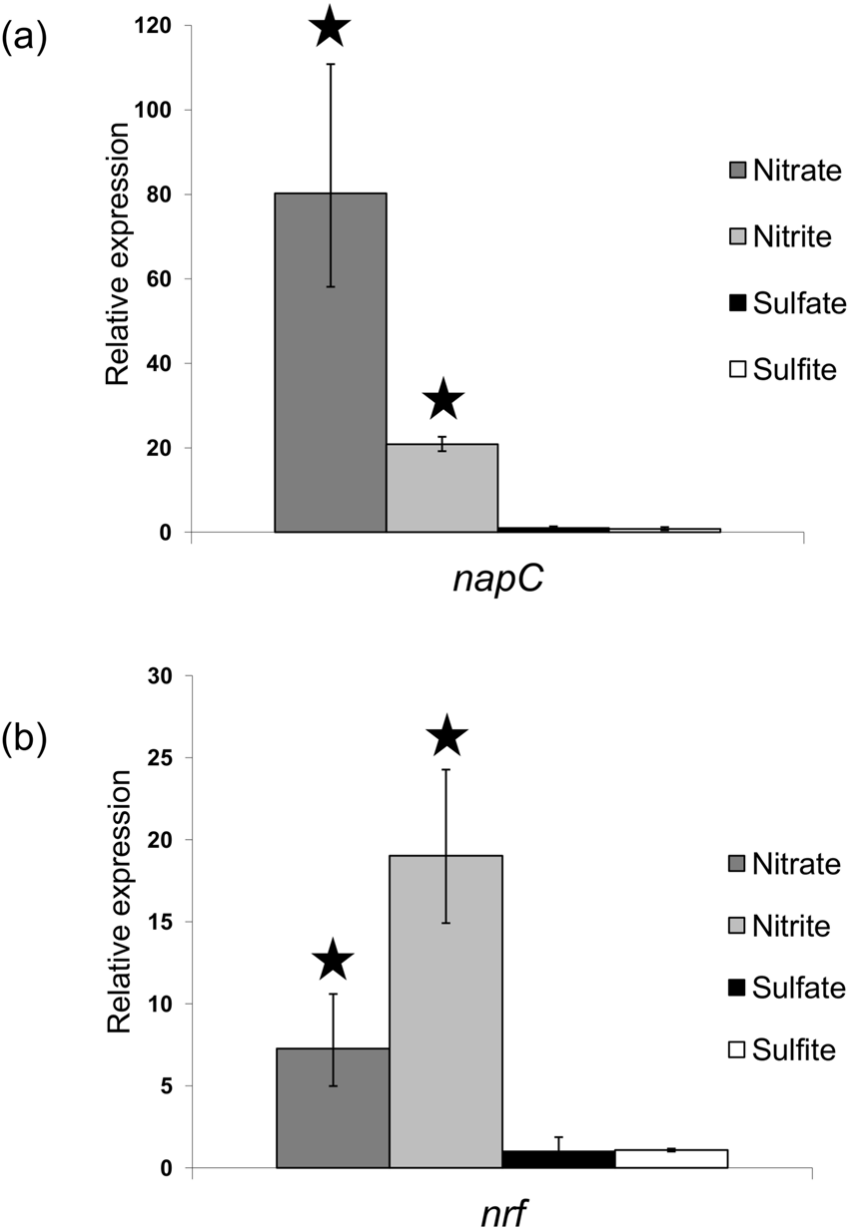
qRT-PCR of genes involved in nitrate and nitrite reduction in *D. desulfuricans*. RNA was purified from cells grown on medium containing nitrate, nitrite, sulfate or sulfite as the sole terminal electron acceptor. RNA was reverse-transcribed with random hexamers. Transcript levels were normalised against *polA* levels. Expression levels are derived from three biological replicates and are normalised to those given by sulfate grown cells. Stars indicate data derived from ΔCt values statistically significantly different to that for sulfate-grown cultures.

RNA was isolated from cultures growing exponentially in the presence of sulfate, sulfite, nitrate or nitrite. Levels of *nap* and *nrf* transcripts were assayed by qRT-PCR. The *napC* mRNA was 80-fold more abundant during growth with nitrate than in the sulfate control, and 20-fold higher during growth in the presence of nitrite, but less abundant in the sulfite cultures than in the sulphate cultures (Fig. 2a).

Transcription at the *nrfA* promoter was also induced 6-fold during growth in the presence of nitrate, in agreement with the RNA-seq data, but far more highly induced, 18-fold, during growth with nitrite than with nitrate (Fig. 2b). Levels of *nrfA* transcripts in cultures growing with sulfate or sulfite were similar. These results demonstrated that, although expression of both *nap* and *nrf* are regulated in response to the availability of nitrate or nitrite, they are not regulated coordinately.

### Global response of the D. desulfuricans transcriptome to nitrate and NO

RNA-seq was used to determine the full extent of changes in the transcriptome in response to replacing sulfate by nitrate as the terminal electron acceptor to support growth. Some of the many anticipated changes would be direct effects on genes required for nitrate reduction or for energy conservation. Secondary consequences were anticipated due to the production of nitrite, which is toxic. Finally, some nitric oxide is always generated as a side product during nitrite reduction to ammonia. This would result in a nitrosative stress response. As a first step to distinguish between responses to nitrate or to nitrosative stress, RNA was isolated from bacteria during growth under four conditions: sulfate as the only terminal electron acceptor (control); nitrate as the only electron acceptor; sulfate-grown cultures supplemented with 7.5 μM NO; and nitrate-grown cultures supplemented with 7.5 μM NO. Consistent with previous reports, the yield in the nitrate cultures was almost double that of the sulfate control, but there was a lag in growth before growth on nitrate commenced^2,9,14,39^. As the addition of NO also caused a lag in growth, bacteria for RNA analysis were harvested after growth had resumed, which was 4 h after the addition of NO when most of the NO had been reduced. Quantitative RT-PCR data had previously shown that similar transcription responses were detected for more than 20 h after NO addition^30^, so the 4 h exposure allowed the response to NO to be determined. Note, however, that nitrite was generated in cultures supplemented with nitrate, some of which was converted to NO, though the majority was reduced to ammonia. Supplementary Table S2 indicates the overall number of reads per sample at the different steps of the analysis (sequencing, genome mapping, gene assignment). The analysis of inter-sample correlation (Supplementary Fig. S1) shows a consistent grouping according to the electron acceptor (Nitrate or Sulfate). However, within the Sulfate cluster, samples SN1 and S1 are separated from the others. They were therefore considered as outliers and discarded from the analysis of differential expression. Additional statistical analysis that justifies the omission of these data are provided as a technical report at the end of the Supplementary Information. The combined data allowed 4 sets of differential analyses to be completed (Table 1).

**Table 1:** Summary of the differentially expressed gene analysis.

Growth conditions compared	Replicates	Differentially expressed genes	Up-regulated genes	Down regulated genes	Location of data
Sulfate + NO *v* sulfate	2 *v* 2	57	31	26	Table 4
Nitrate + NO *v* nitrate	3 *v* 3	20	18	2	Table S5
Nitrate *v* sulfate	3 *v* 2	672	310: *Table 2	362: *Table 3	Table S3
Nitrate + NO *v* sulfate + NO	3 *v* 2	447	211	236	Table S6

*Genes most strongly up- or down-regulated during growth in the presence of nitrate compared with growth in the presence of sulphate.

### Response of the D. desulfuricans transcriptome to growth in the presence of nitrate instead of sulfate

**Figure 3:**
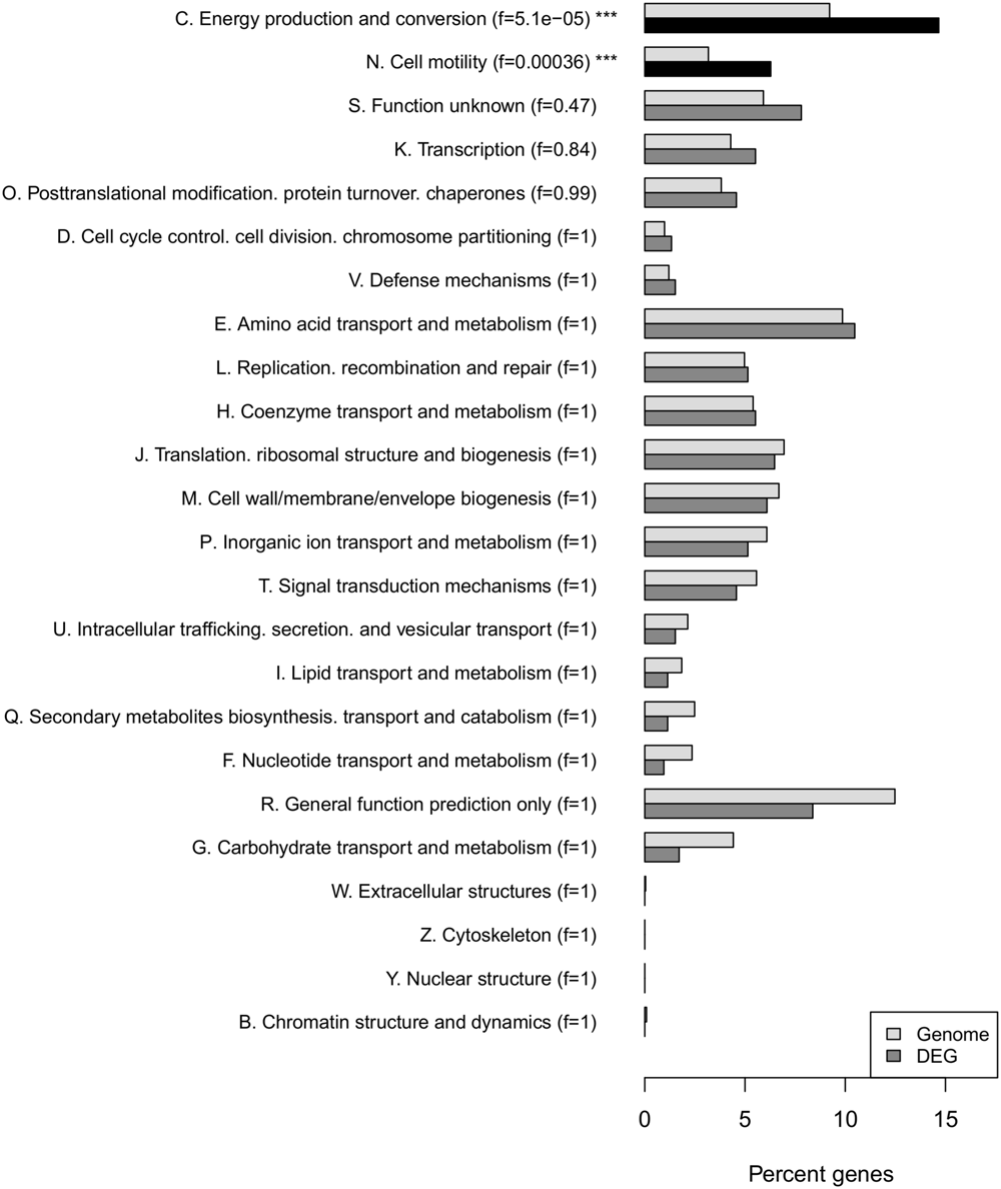
Distribution of the differentially expressed genes in COG categories (in percentage) during growth with nitrate compared to sulfate (in dark grey) and in the whole *D. desulfuricans* genome (in light grey). The two filled back bars and asterisks beside the first two entries indicate the COG classes showing significant over-representation (f < 0.001, where “f” stands for Family-Wise Error Rate). Full details of the statistical analysis of the data are provided in the Supplementary Information as a technical report and Supplementary Table S6.

Expression of 310 genes was significantly higher and expression of a further 362 genes was lower during growth in the presence of nitrate compared with growth in the presence of sulphate (Supplementary Table S3). For simplicity, these differences in gene expression are subsequently referred to as induction and repression without implying specific mechanisms, which are unknown. It suggests that switching from sulfate to nitrate respiration involves a rather large transcriptomic change (variation of expression of about 27% of the genes). Note that these groups should include genes specifically induced or repressed not only by nitrate, but also by nitrite and NO generated from nitrate, and therefore should be the largest groups of genes found to be differentially regulated in the study. As this list will include many transcripts that respond to secondary or even tertiary consequences of nitrate reduction, transcripts most strongly induced or repressed by nitrate or its reduction products are listed in Tables 2 and 3, respectively. Comparison of the COG distribution of the genes differentially expressed with the *D. desulfuricans* whole genome COG distribution revealed that the C (energy production and conversion) and N (cell motility) categories were significantly over-represented in the differentially expressed gene lists compared to the whole genome (Fig. 3 and Supplementary Table S4), showing that these categories were likely important for switching from sulfate respiration to nitrate respiration.

**Table 2:** Genes most highly induced by growth with nitrate instead of sulfate as electron acceptor.

Gene ID	Likely function	log_2_FC^1^	Padj^2^
Ddes_0021	NLP – P60 protein	1.63	0.004
Ddes_0081	Cytochrome c nitrite reductase, NrfA	4.04	0.00037
Ddes_0082	NrfH; electron donor to NrfA	3.57	0.000664
Ddes_0097	ErfK-family protein	1.91	7.65e-5
Ddes_0305	Unknown	2.8	0.000309
Ddes_0311	Unknown	2.46	3.91e-5
Ddes_0312	Glycosyl transferase family 9	1.75	0.00129
Ddes_0333	Major facilitator family membrane transport protein	1.79	0.00336
Ddes_0334	Prephenate dehydrogenase	1.85	0.00164
Ddes_0335	3-phosphoshikimate 1-carboxyvinyltransferase	2.29	4.16e-5
Ddes_0336	Chorismate mutase	2.47	3.53e-6
Ddes_0337	3-dehydroquinate synthase	1.87	0.00292
Ddes_0525	4Fe-4S ferredoxin family	1.94	0.00030
Ddes_0526	Pyridoxamine 5′-phosphate oxidase-related FMN-binding	2.6	1.67e-6
Ddes_0527	Flavodoxin family protein	2.05	9.34e-5
Ddes_0528	CRP-family transcription factor HcpR1	1.85	0.00109
Ddes_0545	2-hydroxyglutaryl-CoA dehydratase D-component	3.97	0.0004
Ddes_0614	Periplasmic nitrate reductase, NapC	3.67	1.92e-10
Ddes_0615	NapM	3.74	1.26e-10
Ddes_0616	NapA	3.25	3.24e-7
Ddes_0617	NapD	2.83	0.000187
Ddes_0619	NapH	1.98	0.0186
Ddes_0625	Unknown	3.53	3.71e-6
Ddes_0641	Alanine-glyoxylate transaminase	2.71	0.00178
Ddes_0695	Unknown	2.27	0.000168
Ddes_0786	Glycine cleavage system T protein	2.63	0.00375
Ddes_0787	Glycine cleavage system H protein	2.99	0.000285
Ddes_0789	Glycine dehydrogenase protein 2	1.87	0.000372
Ddes_0822	ABC-type glycine betaine transport system	2.94	0.000142
Ddes_0843	Rrf2 family transcription regulator	3.81	2.39e-5
Ddes_0844	Receiver domain response regulator	3.55	3.15e-6
Ddes_0851	Glucose-6-phosphate isomerase	2.25	3.90e-5
Ddes_0884	Unknown	2.47	6.82e-5
Ddes_0981	Unknown	2.55	3.20e-5
Ddes_1000	Efflux pump-like protein	2.9	2.76e-7
Ddes_1028	Flagellin domain protein	2.91	0.00107
Ddes_1118	Cell division protein FtsZ	2.43	9.43e-5
Ddes_1176	Triose phosphate isomerase	2.39	5.59e-5
Ddes_1238	NADH dehydrogenase 51 kDa subunit	2.33	0.00194
Ddes_1239	NQR2 and RnfD family protein	2.61	1.99e-5
Ddes_1240	FMN-binding protein	2.79	5.70e-5
Ddes_1241	Electron transfer complex protein	2.78	1.11e-5
Ddes_1242	Electron transfer complex protein	3.42	7.7e-9
Ddes_1243	4Fe-4S ferredoxin iron-sulfur protein	3.94	1.87e-8
Ddes_1244	Lipoprotein	2.96	2.86e-6
Ddes_1259	Flagella hook-length controlling protein	2.3	0.000148
Ddes_1260	Flagella hook capping protein	2.29	1.99e-5
Ddes_1261	Unknown	2.33	0.00443
Ddes_1528	Fumarate-tartrate hydrolyase iron-sulfur α subunit	3.77	8.22e-8
Ddes_1529	Fumarate-tartrate hydrolyase iron-sulfur β subunit	3.36	1.11e-7
Ddes_1530	Fumarate reductase trans-membrane subunit	3,55	6,00e-7
Ddes_1531	Fumarate reductase flavoprotein	3,94	2,77e-6
Ddes_1534	Malate dehydrogenase	2.43	0.00897
Ddes_1559	Unknown	1.92	0.00219
Ddes_1573	Flagella M-ring protein FliF	1.94	0.00314
Ddes_1574	Flagella hook-basal body complex subunit FliE	2.12	0.00198
Ddes_1575	Flagella basal-body rod protein FlgC	2.63	0.000137
Ddes_1576	Flagella basal-body rod protein FlgB	2.52	0.00181
Ddes_1587	Tryptophan synthase, α subunit	4.2	1.61e-6
Ddes_1588	Tryptophan synthase, β subunit	3.98	3.36e-5
Ddes_1589	Phosphoribosylanthranilate isomerase	2.87	6.69e-5
Ddes_1590	Indole-3-glycerol-phosphate synthase	3.15	3.32e-7
Ddes_1591	Anthranilate phosphoribosyltransferase	2.43	0.000678
Ddes_1668	4Fe-4S ferredoxin NADH-dependent dehydrogenase	4.16	6.11e-9
Ddes_1669	NADH-quinone oxidoreductase large subunit	2.1	0.00277
Ddes_1671	NADH-ubiquinone oxidoreductase 20 kDa subunit	3.23	2.99e-6
Ddes_1672	NADH dehydrogenase subunit 1	5.29	5.82e-8
Ddes_1673	NADH dehydrogenase (quinone)	2.1	0.000198
Ddes_1829	Hybrid cluster protein, Hcp	8.0	0.0008
Ddes_1846	FAD-dependent NAD(P)H-disulphide oxidoreductase	3.99	1.53e-5
Ddes_1847	Unknown	4.06	4.70e-7
Ddes_2002	Flagella assembly: FlgN family protein	3.81	5.06e-6
Ddes_2003	Flagellar protein FlgJ	2.34	0.00022
Ddes_2004	Flagellar P-ring protein	2.18	0.000532
Ddes_2106	ABC transport protein	2.63	9.09e-5
Ddes_2202	NAD-dependent epimerase/dehydratase	1.86	0.000454
Ddes_2205	Oxygen-independent coproporphyrinogen III oxidase	2.35	2.99e-5
Ddes_2334	Anaerobic cobalt chelatase	3.12	5.59e-5

^1^FC: Fold Change.

^2^Padj: adjusted p-values: multiple testing correction computed by the Benjamini-Hochberg method^72^.

**Table 3:** Genes most highly repressed during growth with nitrate as electron acceptor.

Gene ID	Likely function	log_2_FC^1^	Padj^2^
Ddes_0018	Response regulator receiver protein	−2.72	4.93e-7
Ddes_0032	Tryptophanyl-tRNA synthetase	−3.11	3.04e-8
Ddes_0111	Small hypothetical protein	−4.01	2.43e-11
Ddes_0112	Sarcosine reductase	−3.07	3.39e-7
Ddes_0113	Glycine/betaine/sarcosine/D-proline reductase family	−2.89	5.39e-7
Ddes_0114	Thioredoxin reductase	−3.06	8.77e-8
Ddes_0205	Unknown	−3.68	3.04e-6
Ddes_0219	Sigma54 specific transcriptional regulator, Fis family	−3.44	2.87e-7
Ddes_0226	Unknown function	−3.3	1.77e-11
Ddes_0227	Unknown function	−1.61	0.00193
Ddes_0408	Contains MurG-like glycosyltransferase domain	−3.48	4.73e-7
Ddes_0430	AraC family transcription factor	−2.79	5.31e-7
Ddes_0446	Metal dependent phosphohydrolase	−3.19	6.08e-7
Ddes_0477	Dihydrodipicolinate reductase	−3.51	2.65e-6
Ddes_0493	RNP-1 like RNA-binding protein	−2.89	9.73e-9
Ddes_0644	FeoA family protein	−3.05	4.73e-7
Ddes_0645	FeoA family protein	−3.92	1.96e-8
Ddes_0646	Small GTP-binding protein	−1.95	0.000582
Ddes_0647	Unknown	−2.64	1.28e-5
Ddes_0648	Unknown	−2.66	2.29e-8
Ddes_0768	PAS/PAC sensor signal transduction histidine kinase	−2.98	5.03e-06
Ddes_0819	Putative phage repressor	−4.22	7.29e-9
Ddes_0847	Unknown	–3.22	1.22e-10
Ddes_0897	Rubrerythrin	−4.3	1.40e-8
Ddes_1104	MraZ protein	−3.33	7.65e-9
Ddes_1161	AraC family transcription factor	−3.14	3.2e-13
Ddes_1247	Unknown	−4.74	4.62e-11
Ddes_1344	Biopolymer transport protein ExbD/TolR	−4.13	1.59e-13
Ddes_1345	Isochorismate synthase	−2.21	0.000518
Ddes_1346	Chorismate mutase related enzyme	−4.14	2.99e-10
Ddes_1484	Unknown	−3.21	2.6e-11
Ddes_1494	Catalase	−4.42	1.09e-7
Ddes_1585	Ferrous iron transport protein like FeoB	−1.41	0.00266
Ddes_1586	Unknown	−5.01	1.46e-6
Ddes_1661	NADH dehydrogenase	−3	3.03e-8
Ddes_1702	Unknown	−2.56	5.06e-6
Ddes_1729	ATPase associated with various cellular activities	−4.53	1.4e-8
Ddes_1750	FeoA family protein	−5.22	1.96e-13
Ddes_1864	Dinitrogenase iron-molybdenum cofactor biosynthesis	−5.35	1.68e-15
Ddes_1865	Cobyrinic acid ac-diamide synthase	−3.9	1.91e-9
Ddes_1866	4Fe-4S ferredoxin iron-sulfur protein	−3.63	1.57e-6
Ddes_1867	Dinitrogenase iron-molybdenum cofactor biosynthesis	−5.31	5.82e-10
Ddes_1925	Putative ArsR family transcription factor	−3.33	2.86e-6
Ddes_1951	Flavodoxin	−4.19	8.11e-7
Ddes_2223	Unknown	−3.03	2.27e-9
Ddes_2233	XRE family transcription factor	−3.13	9.32e-8

^1^FC: Fold Change.

^2^Padj: adjusted p-values, multiple testing correction computed by the Benjamini-Hochberg method^72^.

The genes most highly induced during growth with nitrate included the expected periplasmic nitrate reductase, Ddes_0614–0619, and nitrite reductase, Ddes_0081–0082 (Table 2 and Supplementary Table S3). It also includes two clusters, Ddes_1668–1673 and Ddes_1238–1244, potentially encoding respiratory NADH-dehydrogenases that transfer electrons from NADH to the quinone pool. Particularly intriguing is the strong induction by nitrate of a two-component regulatory system in which Ddes_0843 encodes an Rrf2 family sensor protein and Ddes_0844 encodes a receiver domain protein. Why genes for aromatic amino acid biosynthesis and flagella proteins are also induced during growth in the presence of nitrate remains to be determined. Note also the strong induction by nitrate of the fumarate reductase genes. This was surprising because in enteric bacteria expression of the fumarate reductase operon is repressed by nitrate^40–42^.

The genes most significantly repressed in the presence of nitrate included a hypothetical gene transfer island, Ddes_0702–0725, that were repressed up to 100-fold (Supplementary Table S3). Other strongly repressed genes encode proteins for various iron uptake mechanisms, and a cluster of genes for nitrogen fixation that are probably in an operon (Table 3 and Supplementary Table S3). Genes for six putative transcription factors were repressed by nitrate: the AraC family transcription factor encoded by Ddes_0430; the σ^54^-dependent Fis family transcription factor encoded by Ddes_0219; the ArsR family transcription factor encoded by Ddes_1925; the PAS/PAC sensor histidine kinase encoded by Ddes_0768; the response regulator receiver protein encoded by Ddes_0018; and an XRE family transcription factor encoded by Ddes_2233. This is in agreement with the fact that switching from sulfate to nitrate as terminal electron acceptor requires a fine tuning of the metabolic pathways controlled by several transcription factors. As previously reported the gene for the hybrid cluster protein, *hcp* (Ddes_1829) was induced in the presence of nitrate (Supplementary Table S3). It was intriguing that genes encoding two enzymes involved in hydrogen peroxide scavenging, catalase (Ddes_1494) and the rubrerythrin (Ddes_0897), were repressed by nitrate.

### Global effects of nitric oxide on gene expression

Comparison of data from sulfate grown cultures supplemented with NO with the sulfate control culture revealed that 31 genes were induced specifically by externally supplied NO, and 26 were repressed (Table 4). The two most strongly induced clusters were Ddes-0525 to 0528 and Ddes1828–1829 that are probably in two operons. The first group includes genes for an iron-sulfur protein, an FMN-binding protein, a flavodoxin, and Ddes_0528 encoding the NO-responsive transcription factor, HcpR1. The second group includes Ddes_1829 encoding the hybrid cluster protein (Hcp). Equally significant is the absence of induction of gene Ddes_1827 encoding the transcription factor, HcpR2. This confirmed our recent report that, although HcpR2 regulates Hcp synthesis in response to the availability of NO, *hcpR2* expression was not induced by NO^30^. Two further clusters, Ddes_0288–0289 and Ddes_0334–0338 predicted to encode proteins for heme synthesis and in aromatic amino acid biosynthesis, respectively, were also up-regulated in the presence of NO.

**Table 4:** Genes differentially expressed in the presence of exogenous NO with sulfate as electron acceptor.

Gene Id	Name	Description	log_2_FC1	Padj^2^	COG
Ddes_0111		Small hypothetical protein	−1.5	0.01	
Ddes_0153		OsmC family protein	−1.63	0.019	O
Ddes_0288		Porphobilinogen synthase	1.63	0.00303	H
Ddes_0289		SAM-binding methylase	2.41	1.59E-06	R
Ddes_0290	*alaS*	Alanyl-tRNA synthetase	1.49	0.0132	J
Ddes_0334		Prephenate dehydrogenase	1.52	0.0206	E
Ddes_0335		3-phosphoshikimate 1-carboxyvinyltransferase	1.35	0.0377	E
Ddes_0336		Chorismate mutase	1.56	0.00875	E
Ddes_0337		3-dehydroquinate synthase	1.45	0.0119	E
Ddes_0338		Fructose-bisphosphate aldolase	1.34	0.0334	G
Ddes_0339		Pyridoxal phosphate-dependent D-cysteine desulfhydrase family	−1.67	0.0392	E
Ddes_0382	*cooS*	Carbon monoxide dehydrogenase	2.01	0.0119	C
Ddes_0408		MurG-like glycosyltransferase domain containing protein	−1.99	0.00875	
Ddes_0524		HPP family transmembrane protein	1.75	0.00695	T
Ddes_0525		[4Fe-4S] iron-sulfur protein	2.16	3.77E-05	C
Ddes_0526	*wrbA*	Flavin mononucleotide binding protein	3.07	2.62E-10	R
Ddes_0527	*nimA*	Flavodoxin family protein	2.68	7.62E-09	C
Ddes_0528	*hcpR1*	Crp/Fnr family transcriptional regulator	3.05	2.62E-10	T
Ddes_0663	*rplB*	50 S ribosomal protein L2	1.23	0.0474	J
Ddes_0664	*rpsS*	30 S ribosomal protein S19	1.42	0.023	J
Ddes_0665	*rplV*	50 S ribosomal protein L22	1.58	0.0191	J
Ddes_0666	*rpsC*	30 S ribosomal protein S3	1.47	0.0206	J
Ddes_0703		Hypothetical; part of a putative gene transfer agent (GTA) island	−1.66	0.0169	
Ddes_0704		Hypothetical; part of a putative GTA island	−1.6	0.0474	
Ddes_0705		Hypothetical; part of a putative GTA island	−1.72	0.0474	
Ddes_0706		Hypothetical; part of a putative GTA island	−1.97	0.0164	
Ddes_0707		Hypothetical; part of a putative GTA island	−1.84	0.0372	
Ddes_0708		Hypothetical; part of a putative GTA island	−1.86	0.0306	K
Ddes_0710		Hypothetical; part of a putative GTA island	−1.84	0.0468	
Ddes_0713		Hypothetical; part of a putative GTA island	−2.12	0.0474	
Ddes_0715		Hypothetical; part of a putative GTA island	−2.07	0.0474	
Ddes_0824		Glycine betaine/L-proline ABC transporter ATPase	−1.22	0.0474	E
Ddes_0935		Short coiled-coil protein	−1.41	0.0164	
Ddes_1070		Insulinase-like protease; peptidase M16 domain-containing protein	1.32	0.0448	R
Ddes_1077		BadM/Rrf2 family transcriptional regulator	1.24	0.0434	K
Ddes_1165		YtfE-like protein containing hemerythrin diiron and PAS domains	1.98	0.0209	S
Ddes_1166		Short hypothetical protein	1.38	0.0209	
Ddes_1208		Periplasmic chaperone/protease	2.9	0.0206	O
Ddes_1269		Molybdenum cofactor biosynthesis protein A	1.26	0.0392	H
Ddes_1324		Kinase	−1.47	0.0498	R
Ddes_1427		Hypothetical protein	−1.5	0.00864	
Ddes_1468		O-acetylhomoserine/O-acetylserine sulfhydrylase; methionine biosynthesis	1.74	0.00488	E
Ddes_1502		FeFe Hydrogenase/Ferredoxin hydrogenase	1.24	0.0392	
Ddes_1585		Ferrous iron transport protein B: FeoB-like	1.74	0.0392	P
Ddes_1642		Small hypothetic protein of unknown function	−1.85	0.019	
Ddes_1643		Sigma 54 interacting domain-containing protein	−1.45	0.0169	T
Ddes_1644		Pyruvate phosphate dikinase	−1.21	0.0474	G
Ddes_1824	*moeA*	Molybdenum-binding protein	−2.14	0.00264	H
Ddes_1828		Cupin fold protein	4.4	9.30E-07	S
Ddes_1829	*hcp*	Hybrid cluster protein: NO reductase	3.17	1.61E-06	C
Ddes_2104		Membrane protein	2.31	0.000974	S
Ddes_2105		460 aa transmembrane protein	1.6	0.0169	V
Ddes_2130	*apsB*	Adenylylsulphate reductase β subunit	−1.83	0.0434	C
Ddes_2132		Hypothetical protein	−1.3	0.0406	
Ddes_2135		Putative NiFe hydrogenase	−1.33	0.0314	
Ddes_2150	*ssc*	Split-Soret cytochrome c	−1.74	0.0206	
Ddes_2235		Cdc6-like protein containing AAA + and winged-helix domains	−1.56	0.0372	

Note the absence of any significant difference in the level of expression of the *hcpR2* gene, Ddes_1827.

^1^FC: Fold change.

^2^Padj: adjusted p-values: multiple testing correction computed by the Benjamini-Hochberg method^72^.

At least two di-iron proteins protect enteric bacteria against nitrosative stress. One is the flavorubredoxin, NorV, with its characteristic β-lactamase fold. The second is the hemerythrin-like di-iron containing domain protein, YtfE (also known as RIC)^43–47^. An orthologue of NorV has been identified in *D. gigas*: this is the flavodiiron protein, ROO^23,26^, which is also the orthologue of Ddes_2012. Surprisingly, until the current work, no orthologue of YtfE has been found in sulphate reducing bacteria. It is therefore significant that expression of D_des 1165 was strongly induced under conditions of nitrosative stress (Table 4). HHpred analysis indicates that the N-terminus of Ddes_1165 is predicted to adopt a fold like that of YtfE from *E. coli* (probability 99.3%) whilst the C-terminus likely adopts a PAS domain-type fold^48^. Crucially, Ddes_1165 residues H234, H262 and E239 are equivalent to YtfE residues H129, H160 and E133 which are involved in co-ordination of the di-iron centre^49^. Analysis of the Ddes_1165 sequence with the tool PHMMER also identifies the presence of hemerythrin-like and PAS domains in this protein. PAS domains are involved in diverse processes but are mediators of intermolecular interactions including protein:protein interactions, a function consistent with the proposed role of YtfE interacting with iron-sulfur cluster proteins^43–47^. Interestingly, in other studies, the *D. vulgaris* homolog of Ddes_1165 (DVU2590) was found to be transiently up-regulated by nitrite stress but unaffected by oxidative stress, demonstrating a nitrosative stress-specific response by this gene product^50,51^. We propose that Ddes_1165 is the equivalent of YtfE in *D. desulfuricans* and related SRB.

The predicted functions of NO-repressed genes varied greatly, suggesting that many of them might be secondary responses, for example, to nitrosative damage to various enzymes and transcription factors. This rather limited group included Ddes_1643, which is predicted to encode a σ^54^-dependent transcription factor, and Ddes_2150 encoding the precursor of the “Split Soret” cytochrome *c*. Note that based upon Northern blot analysis, it was previously reported that expression of Ddes_2150 is more induced during growth with nitrate than with sulfate^52^. The decreased expression of the large cluster from Ddes_0703 to 0715 encoding a hypothetical gene transfer island might be due either to repression or to gene loss in response to NO exposure (Table 4). With the exception of the Ddes_0663 to Ddes_0666 cluster, genes for ribosomal proteins were absent from the list of NO-repressed genes, suggesting that the differential analysis revealed responses to NO rather than to a decreased growth rate.

The effect of NO on cultures growing with nitrate as the terminal electron acceptor was also determined. Fewer genes were differentially expressed compared to when sulfate was used as terminal electron acceptor: 18 genes were induced, and only 2 were repressed (Supplementary Table S5). This result was consistent with our prediction that some NO will have been generated endogenously from nitrite formed as a product of nitrate reduction. Therefore, some NO-responsive genes would be induced or repressed even during growth in the presence of nitrate alone. This would decrease any additional response to NO when nitrate is also present. Responses to NO were found for the Ddes_0524–0528 gene cluster, confirming that transcription of these genes responds to NO rather than to nitrate, which was present in all of these cultures. Perhaps the most significant result is that the genes of previously unknown function, Ddes_1164 and Ddes_1165, were more strongly induced in the presence of both nitrate and NO than in the absence of nitrate. This strongly supports our proposal above that the diiron protein encoded by Ddes_1165 is the orthologue of YtfE in enteric bacteria.

Only two genes, Ddes_1501 encoding a small GTP-binding protein, and Ddes_1581, encoding a hypothetical protein, were significantly repressed by exogenous NO in cultures growing with nitrate. These genes were not found to be differentially expressed in sulfate + NO *versus* sulfate conditions. Ddes_1501 was up-regulated in nitrate *versus* sulfate conditions, suggesting that it is important in nitrate respiration but its expression is down-regulated as a stress response to NO (Supplementary Table S3). Note that neither of the genes encoding enzymes involved in hydrogen peroxide scavenging, catalase (Ddes_1494) and the rubrerythrin (Ddes_0897), were differentially expressed when the cultures were challenged with exogenous NO with either nitrate or sulfate as electron acceptor. This suggests that their expression is not dependent on the presence of exogenous NO but rather to reaction products linked to the nature of the electron acceptor.

Only 7 genes responding to the presence of exogenous NO were found in common under sulfate and nitrate conditions. These included the gene cluster Ddes_0524–0528 as well as the genes Ddes_1165 and Ddes_0288. All of these genes were found to be induced in the presence of exogenous NO. Except for Ddes_1165, they were also found significantly up-regulated in nitrate versus sulfate conditions (Supplementary Table S3).

These results indicate that the genes induced by NO are almost certainly required to protect bacteria from nitrosative stress in one of two ways. Some such as Hcp (Ddes_1829) are involved directly as NO scavenging systems. Others such as enzymes involved in amino-acid metabolism might be involved indirectly, for example by limiting toxic effects of NO through mechanisms that need to be determined, or as secondary consequences of nitrosative damage to proteins that regulate their synthesis.

### Confirmation that nitrate partially induces a nitrosative stress response

A final comparison of differentially expressed genes during growth with nitrate rather than sulfate when both sets of cultures were also challenged with NO provided independent confirmation of many of the above results (Supplementary Table S6). About 68% of the up-regulated genes are shared between the two conditions, with and without exogenous NO. The smaller number of transcripts induced by nitrate in the presence of NO than in its absence (211 instead of 310) was again consistent with the proposal that some genes were induced by NO produced from nitrite during nitrate reduction. Ddes_1581 was down-regulated during growth with nitrate + NO compared with sulfate + NO, suggesting that this gene responds to exogenous NO only during nitrate respiration (Supplementary Table S6).

### Validation of selected RNAseq data by quantitative RT-PCR

To validate the RNA-seq data, expression of *hcpR1*, *hcp, hcpR2, nap* and *nrf* in the presence of nitrate, sulphate and nitric oxide were analysed by qRT-PCR (Supplementary Fig. S2a and b). No significant effects of nitrate or NO on *hcpR2* expression were detected by qRT-PCR. The expression profiles of the other targets mirrored those obtained by the RNA-seq analysis, although larger relative changes in expression of *napC* and *hcp* were observed in the qRT-PCR analysis. The RNA-seq data were also consistent with our earlier studies^14,30^ that demonstrated up-regulation of *nap, hcpR1*, and *hcp* and no change in expression of *hcpR2*, in response to nitrate or nitric oxide.

### Bioinformatic prediction of the extent of the HcpR1 regulon

The name HcpR was introduced to designate the transcription factor that regulates synthesis of the hybrid cluster protein and an associated electron transfer protein, FrdX^18,19^. Core regulons for HcpR in *D. vulgaris* and *D. alaskensis* were proposed based upon the presence of R, E and R residues in positions 1, 2 and 6 of the DNA recognition helix and hence its similarity to *E. coli* Crp. These residues are absent from the DNA recognition helix of *D. desulfuricans* HcpR2, but are present in HcpR1, which is only 24–25% identical to HcpR from the other two species and is not co-located with genes for either FrdX or Hcp.

To investigate the possible global role of HcpR1, a position specific scoring matrix (PSSM) strategy was used to scan the *D. desulfuricans* ATCC27774 genome for putative HcpR1 binding sites. A seed matrix derived from the HcpR1 consensus sequence TGTGA-N6-TCACA was used to scan the upstream sequences of all genes^30^ (Supplementary Fig. S3A). Sites having a p-value lower than 10^−4^ were used to build a “second-generation” matrix (Supplementary Fig. S3B), which in turn was used to scan upstream sequence of all genes to gather putative binding sites with more flexibility than the original seed matrix. This analysis returned 91 genes whose upstream region contains at least one predicted HcpR1 binding site with a p-value < 10^− 4^ (Supplementary Table S7). Among them are found the genes *hcpR1* (Ddes_0528) and *napC* (Ddes_0614), for which electromobility shift assays (EMSA) showed that HcpR1 effectively binds to the promoter sequences^30^ with high affinity (Fig. 2). In addition, binding of HcpR1 to promoter sequences of the *sat* (Ddes_0454) and Ddes_1825 genes with a low affinity was also confirmed by EMSA experiments (Supplementary Fig. S4). However, neither of these two genes was differentially expressed in any of the conditions tested (Supplementary Table S7). Out of the 91 genes, 38 were differentially expressed in the nitrate versus sulfate conditions, 13 being up-regulated (including the *napC* gene) and 25 down-regulated (Supplementary Table S7). These 38 genes can be considered as an HcpR1 regulon. Only 5 genes were also similarly differentially expressed in sulfate + NO versus sulfate conditions, Ddes_0528 encoding HcpR1 being the only one up-regulated. The other four genes encode two hypothetical proteins (Ddes_1427 and Ddes_1642), a transcription factor (Ddes_1643) and a molybdopterin-containing protein (Ddes_1824). The much lower number of genes in this case could be linked to a dose-dependent response. We previously showed that purified HcpR1 binds heme to give a complex with oxidized and reduced spectra typical of a *b*-type cytochrome^30^. Based upon this evidence, it has been proposed that HcpR1 is a heme-containing protein able to react with NO, but how NO modulates the transcriptional regulatory function of HcpR1 is still unknown^30^.

## Discussion

Many aspects of the diversity, environmental importance and potential for biotechnological exploitation of sulfate reducing bacteria for biodegradation or in the oil industry have been studied in depth^53–56^. In contrast, knowledge of their physiology, biochemistry, intermediary metabolism and gene regulation is far more limited. There are many gaps in knowledge of how electrons are transferred from primary dehydrogenases to a diverse range of terminal electron acceptors despite the availability of elegant structures of some of the redox protein complexes involved^48,57–63^. It is therefore potentially significant that the current study showed that the expression of genes for two NADH dehydrogenases is induced by nitrate, but a third potential NADH dehydrogenase, Ddes_1661, is repressed. This suggests that *D. desulfuricans* might form large electron transfer complexes in which proteins that reduce specific terminal electron acceptors form complexes with specific dehydrogenases, and that their synthesis might therefore be co-ordinately regulated. As current knowledge of operon structures and transcription start sites is extremely limited, mechanisms of gene regulation remain speculative.

We have previously reported that nitrate reduction by *D. desulfuricans* 27774 is tightly regulated by nitrate induction, which is over-ridden by sulfate repression^14^. Nitrate reduction is also strongly inhibited by sulfide generated from sulfate or sulfite reduction. We also proposed that the NO-sensitive transcription factor, HcpR1, is involved in the regulation of nitrate reduction^30^. In the current paper we demonstrated that, as expected, nitrite reduction is also tightly regulated, presumably by the NrfR-NrfS two-component regulatory system^11,15^ (Fig. 2). There is an almost perfect NrfR consensus binding site in the *napM* regulatory region, implying that the NrfR-NrfS system co-ordinately regulates nitrate and nitrite reduction to minimize the accumulation of nitrite. Although the pattern of regulation of *nrfA* expression is similar to our previously reported regulation of *hcpR1* and *hcp* expression, there are no potential binding sites for HcpR1 or HcpR2 in the *nrfHA* regulatory region, and while *nap* and *hcpR1* expression are regulated by HcpR1, it is HcpR2 that regulates *hcp* expression in response to the presence of NO. Clearly there are links between regulation by HcpR1, HcpR2 and NrfS-NrfR that merit further research. Use of alternative promoters appears to be the most likely mechanism for NrfR-dependent induction of the *nap* and *nrfHA* genes^11^. However, superimposed upon regulation of *nap* gene expression by NrfR is regulation by sulfate, nitrate, HcpR1 and hence by NO. The metabolic signal to which NrfS responds, nitrate or nitrite, remains to be revealed: we suggest it is most probably nitrite because the NrfR- NrfS system is present in many sulphate reducing bacteria that are able to reduce nitrite, but not nitrate. The mechanism by which HcpR2 activates *hcp* synthesis in response to the presence of NO also remains to be determined. Detailed molecular biological experiments will be required to reveal the mechanism of how HcpR1 regulates expression of the *nap* and *hcpR1* genes, or how NO induces HcpR2-dependent expression of the *hcp* operon. What is becoming clear is that there are widely different levels of complexity in gene regulation in this bacterium. While this manuscript was in review, a paper describing the proteomic response of *D. desulfuricans* 27774 to nitrate was published on line. Only proteins soluble in a low ionic strength buffer were analysed, so inevitably there are major differences between the proteomic and our RNAseq data. However, increased accumulation of Nap proteins in response to nitrate was confirmed^64^.

Unexplained is why genes for aromatic amino acid biosynthesis are strongly induced during growth in the presence of nitrate, but genes for catalase and putative iron uptake are repressed. A possible explanation for the induction of fumarate reductase genes during growth with nitrate might be an increased need for succinyl CoA for heme synthesis, but this is mere speculation. These are just a few of the many research challenges revealed from the genome-wide RNA-seq data available from this study. Many genes were differentially repressed by nitrate compared to sulfate when both groups of culture were challenged with NO.

Despite the absence of genetic systems to test many hypotheses based upon results obtained in the current study, the RNA-seq data have revealed many insights into how *D. desulfuricans* responds to an alternative electron acceptor such as nitrate and nitrosative stress. One of the most striking results is the very limited overlap between genes induced by NO or by nitrate. Two noteworthy exceptions were genes encoding nitrate reduction and HcpR1 synthesis, both of which were predicted be regulated by HcpR1^14,19,30^. This indicates that part of the nitrosative stress response is coordinated with a primary cause of stress, the generation of nitrite from nitrate. Consistent with this interpretation is the lower number of differentially expressed genes in response to NO in cultures with nitrate rather than sulfate as the primary electron acceptor (Table 1) and the lower induction or repression ratios observed in cultures supplemented with both nitrate and NO compared with sulfate plus NO (compare Supplementary Tables S2 and S3).

**Figure 4:**
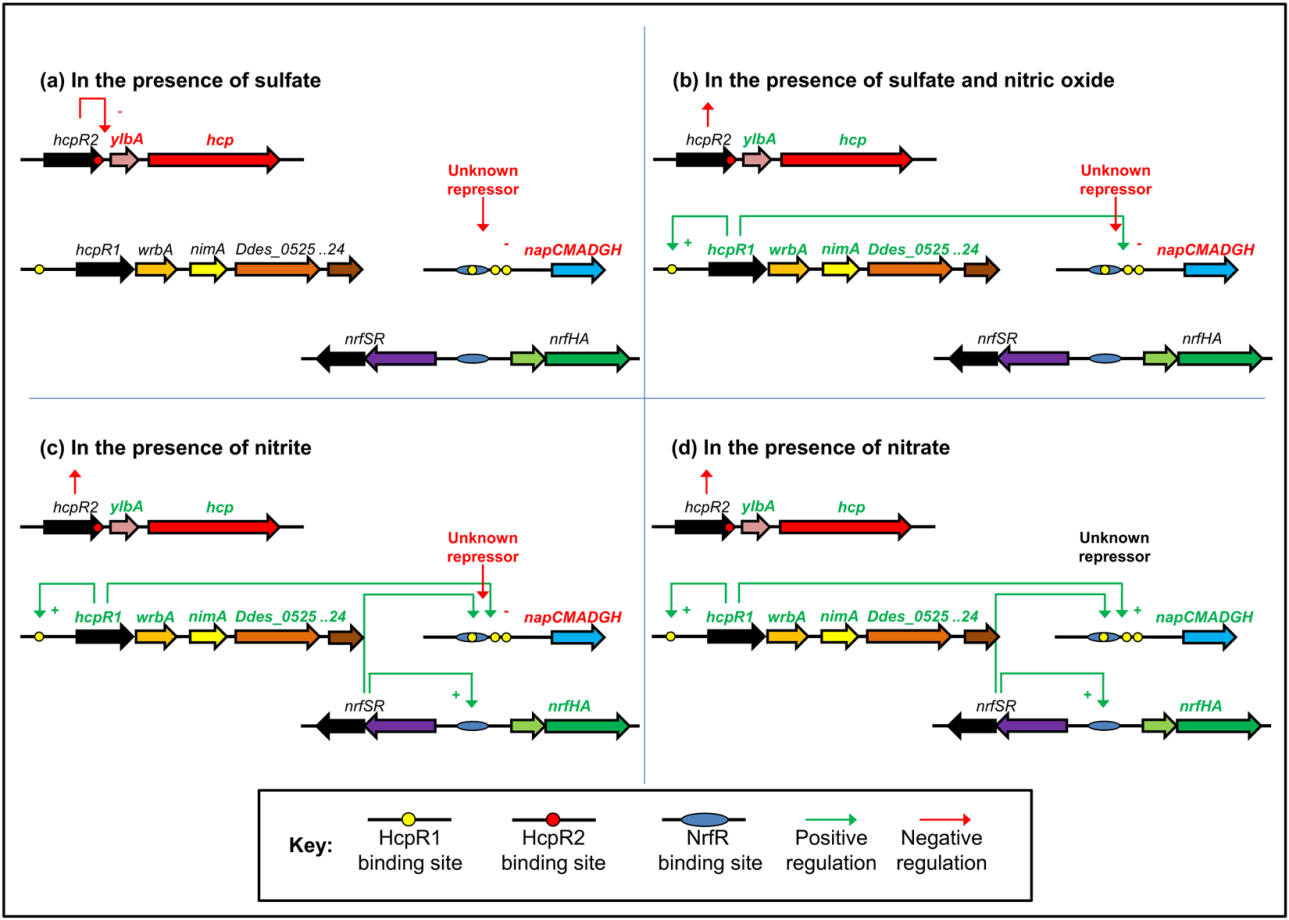
A model of the regulation of key genes involved in the response to nitrate or NO in *D. desulfuricans*. (**a**) In the presence of sulfate alone, HcpR2 binds as a repressor to the downstream DNA target sequence downstream of the *hcpR2* gene, Ddes_1828, so expression of *ylbA* and *hcp* (Ddes_1827 and Ddes_1826, respectively) is repressed. Expression of the *nap* gene cluster, Ddes_0614–0619, is repressed by an unidentified repressor protein^14^. (**b**) In the presence of sulfate and NO, the Fe-S cluster of HcpR2, is damaged by NO leading to the loss of DNA binding by HcpR2 and de-repression of *ylbA* and *hcpI* (Ddes_1827 and Ddes_1826). NO is also sensed by HcpR1, probably via a heme ligand, leading to up-regulation by an unknown mechanism of the *hcpR1-wrbA-nimA-Ddes_0525-Ddes_0524* (genes Ddes_0524-Ddes_0528). (**c**) The presence of nitrite is sensed by the NrfS-NrfR system leading to up-regulation of the divergent *nrfHA* operon (genes Ddes_0081 and Ddes_0082). Both the *hcp* and *hcpR1* operons are expressed since NO is produced as a by-product of nitrite reduction. The presence of both NrfR and HcpR1 in the absence of sulfate leads to a relatively modest increase in expression of *nap*. (**d**) The presence of nitrate in the absence of sulfate leads to full de-repression of the *nap* gene cluster (Ddes_0614 – Ddes_0619) via the action of an unidentified repressor. Co-activation of *nap* is achieved via the activities of HcpR1 and NrfR. Rapid nitrate reduction results in a burst of NO production leading to high levels of HcpR1 synthesis. HcpR1 could also function as a repressor of *nap* expression at high concentrations, providing the regulation of the *nap* cluster with a negative feedback mechanism. Note that this is a minimal model as no roles for sigma-54 or Rex have been included^65^.

A possible model for the regulation of genes for nitrate and nitrite reduction is shown in Fig. 4. The model predicts that in the presence of sulfate but absence of NO (Fig. 4a), HcpR2 is competent for binding the downstream DNA target sequence and so expression of *ylbA* and *hcp* is repressed. Expression of *nap* is repressed by a currently unidentified repressor protein^14^. In the presence of both sulfate and NO, the Fe-S clusters of HcpR2 are damaged by NO leading to the loss of DNA binding by HcpR2 and derepression of *ylbA* and *hcp*. It is likely that NO is also sensed by HcpR1, probably via a heme ligand, leading to increased expression by an unknown mechanism of the *hcpR1-wrbA-nimA-Ddes_0525-Ddes_0524* genes (Fig. 4b). The model assumes that the presence of nitrite is sensed by the NrfS-NrfR system leading to up-regulation of the divergent *nrfHA* operon (Fig. 4c). As NO is produced as a by-product of nitrite reduction, both the *hcp* and *hcpR1* operons are also expressed. The presence of both NrfR and HcpR1 in the absence of sulfate leads to a relatively modest increase in expression of *nap*. Finally, in the presence of nitrate but absence of sulfate, the unidentified repressor of the *nap* gene cluster is inactivated, so *nap* expression is fully de-repressed. It is suggested that co-activation of *nap* is achieved via the activities of HcpR1 and NrfR. Rapid nitrate reduction would result in a burst of NO production leading to high levels of HcpR1 synthesis. HcpR1 could also function as a repressor of *nap* expression at high concentrations, providing the regulation of the *nap* cluster with a negative feedback mechanism. However, this is a minimal model as roles for neither sigma-54 nor Rex have been included. The *nap* and *nrf* promoters have sigma-54 sequence determinants and Rex binds to sites identical to HcpR1 and so these two regulators are also likely to influence expression of these loci^65^.

## Methods

### Media and growth conditions

*D. desulfuricans* strain 27774 was stored at −80 °C as glycerol stocks. Cultures were initially grown in sealed serum bottles at 30 °C in Postgate medium B, which contains both sulfate and ferrous salts^30,66^. For growth and RNA experiments, exponential phase cultures of *D. desulfuricans* were sub-cultured in Postgate Zero medium (NaH_2_PO_4_.2H_2_O, 3 g l^−1^; KH_2_PO_4_, 0.5 g l^−1^; NH_4_Cl, 0.5 g l^−1^; CaCl_2_, 10 mg l^−1^; MgCl_2_.6H_2_O, 25 mg l^−1^; FeCl_2_.4H_2_O, 1.5 mg l^−1^; sodium lactate, 9 ml l^−1^; citric acid, 0.2 g l^−1^; Yeast extract, 1 g l^−1^; pH 7.0). Nitrate, sulfate and sulfite were added to a final concentration of 7.5 mM. NO was added to a final concentration of 7.5 μM. We have previously shown that 7.5 mM nitrite completely inhibits growth, but this strain is able to grow in the presence of sequential additions of nitrite to a final concentration of 2.5 mM, which was therefore the concentration of nitrite used in the growth experiments^14^.

### Electromobility shift assays

DNA fragments containing promoter proximal DNA sequences were amplified from genomic DNA by PCR and cloned in pGEM T Easy vector (Promega). T Easy-derived plasmids containing promoter fragments were purified from *E. coli*, digested with appropriate restriction enzymes, typically EcoRI and HindIII, and then treated with CIP. The excised promoter fragments were purified by electroelution, phenol-chloroform extraction and ethanol precipitation.

Purified DNA fragments were end-labelled with using T4 polynucleotide kinase (New England Biolabs, UK). The end-labelling reaction consisted of 2 µl polynucleotide kinase buffer, 1 µl γ ^32^P-ATP, 10–16 µl of DNA fragment, 1 µl T4 polynucleotide kinase and sterile distilled water to a total volume of 20 µl. Labelling reactions were incubated at 37 °C for 1 hour and then excess γ ^32^P-ATP was removed by passing the reaction mixture through two 200 µl bed volumes of Sephadex G-50 which had be pre-equilibrated with 1 x Tris-EDTA buffer pH 8.0.

EMSA incubation mixtures were prepared to a final volume of 10 µl and included: 0.2–1 µl of radiolabelled DNA fragment (2–4 ng of DNA fragment per EMSA incubation), 1 µl of 10 x binding buffer (20 mM Tris-HCl, pH 8.0; 100 mM KCl; 2 mM MgCl_2_; 10% glycerol (w/v)), 0.5 µl 4 mM spermidine, 0.5 µl 400 ng/µl herring sperm DNA, 0.5 µl 1 mg/ml bovine serum albumin and 1 µl of HcpR1 or HcpR2 at an appropriate concentration. Incubation mixtures were also supplemented with additional additives where noted. The CRP protein used in some of these experiments was kindly provided by Dr. David Lee, University of Birmingham. EMSA mixtures were incubated at 25 °C for 30 minutes and then loaded onto 6% (w/v) acrylamide gels prepared with 0.25 x TBE and 0.2% (v/v) glycerol. Electrophoresis was in 0.25 x TBE at 160 V for 90–180 minutes. Following electrophoresis, gels were fixed in 10% (v/v) acetic acid and 10% (v/v) methanol for 10 minutes. Fixed gels were transferred to 3 mm Whatman filter paper and dried under vacuum. Dried gels were stored in cassettes with a Fuji Imaging Phosphor screen overnight which were then visualised with the Bio-Rad Molecular FX Imager System and QuantityOne software (BioRad).

### DNaseI footprinting assays

Fragments for DNaseI footprinting were liberated from the plasmid pT*nap*WT by digestion with HindIII and then treated with CIP. The digest mixture was purified by phenol-chloroform and ethanol precipitation and then digested with EcoRI. The resulting fragment was purified by electroelution, phenol-chloroform extraction and ethanol precipitation prior to labeling with γ-^32^P-ATP as described previously.

Maxam-Gilbert G + A sequencing reactions were prepared as standards for DNaseI footprinting assays. End-labeled DNA fragments were treated with formic acid for 90 seconds at room temperature and the reaction was stopped by ethanol precipitation. The purified DNA was re-suspended in 1 M piperidine and incubated at 90 °C for 30 minutes. DNA was purified by ethanol precipitation and the DNA pellet re-suspended in loading buffer (20 mM EDTA, 0.05% (w/v) bromophenol blue, 0.05% (w/v) xylene cyanol, dissolved in deionized formamide). G + A reactions were stored at −20 °C until required.

End-labeled DNA fragments were used in *in vitro* DNaseI footprinting assays to map HcpR binding. Incubation mixtures were prepared as described for EMSAs using end-labeled DNA fragments in volumes of 20 µl. After 30 minutes at 25 °C the incubation mixtures were supplemented with a dilution series of DNaseI (Roche Applied Science) ranging from 0–0.003 U and then incubated for an additional 40 seconds prior to inactivation of DNaseI with 200 µl of 500 mM EDTA. Digest mixtures were purified by phenol-chloroform extraction and ethanol precipitation. Purified DNA pellets were resuspended in denaturing gel loading buffer and stored at −20 °C until required.

DNaseI footprinting reactions and G + A sequencing reactions were resolved by electrophoresis on 6% (w/v) acrylamide denaturing urea gels prepared using the SequaGel UreaGel system (National Diagnostics) according to the manufacturer’s instructions. Samples were heated to 90 °C prior to loading onto gels. Electrophoresis was achieved in 1xTBE buffer (89 mM Tris base, 89 mM boric acid, 2.5 mM EDTA) at 60 W for ~2 hours. Gels were fixed, dried and visualised as described for electromobility shift assays.

### RNA isolation and reverse transcription into cDNA

RNA was isolated from 5 to 15 ml samples of cultures in the exponential phase of growth that had been sedimented by centrifugation at 10,000 g for 5 min. Pellets were resuspended in 3 ml of RNAlater (Ambion), snap frozen in liquid nitrogen and stored for up to 2 weeks at −80 °C. Total RNA was purified with the QIAGEN RNeasy mini kit according to the manufacturer’s instructions with the inclusion of at least one on-column DNase digestion step to eliminate any contaminating DNA. RNA was eluted from the RNeasy spin columns with 30 μL RNase–free water and stored at −80 °C for up to 1 month. RNA integrity was assessed with a NanoDrop ND1000c Spectrophotometer (Labtech., UK). Samples with an A_260_/A_230_ ratio of less than 1.8 were rejected, as were samples less concentrated than 200 ng/μL.

RNA was reverse transcribed to cDNA with random hexamer primers using the Tetro cDNA Synthesis kit (BIOLINE, London, UK) and 2 ng of total RNA as template. Multiple RT-PCR reactions were prepared from each sample to provide a cDNA pool.

### Quantitative RT-PCR

A Stratagene Mxp3005 machine set to detect SYBR green fluorescence and 96-well plates capped with optical strip tops was used for qRT-PCR. Reaction mixes were prepared using Brilliant III Ultra-Fast SYBR Green QPCR mastermix kit (Agilent). Gene specific primers to a final concentration of 400 nM and cDNA at a concentration of 5 to 50 ng / μL were added. Cycling parameters were as recommended by the manufacturer except that samples were denatured for 20 s and annealed for 20 s at 56 °C. The dissociation curve for each qPCR reaction was calculated using the pre-programmed Mcp3005 protocol and allowed assessment of PCR efficiency for each reaction. Primer specificity was also checked by agarose gel electrophoresis. Transcript levels were quantified by the ΔΔCt method using the levels of *polA* mRNA as a reference^67^. For each growth condition tested, RNA was prepared from at least three biological replicates and each qPCR reaction was run in triplicate. Thus each qRT-PCR measurement was the result of at least 9 replicates.

### Preparation of RNA samples for RNA-seq analysis

RNA was prepared as described above for up to 20 independent cultures for each growth condition: these were culture growing with sulfate, sulfate plus NO, nitrate, or nitrate plus NO. Each RNA sample was treated twice to remove any traces of DNA. Samples were checked by qPCR for DNA contamination without reverse transcription. The 5 samples with highest concentration and A_260_/A_230_ ratio were transferred on dry ice to Oxford Gene Technologies (UK) for further quality control testing before RNA-seq analysis. Three samples from each growth condition were depleted for ribosomal RNA before reverse transcription.

### Analysis of the RNA-seq data

The automated workflow for the RNA-seq analysis was implemented in the snakemake programming language, and is available on the supporting website. The workflow includes the following sequential steps: *i* > *read quality control* with fastQC (www.bioinformatics.babraham.ac.uk/projects/fastqc/); ii > *read mapping* with bowtie2 in non-oriented paired-ends mode, with at most 1 mismatch per read, and all other options left to their default values^68,69^. The genome sequence (fasta-formatted) and features (GTF-formatted) were downloaded from EnsemblGenomes Bacteria release 32 (http://bacteria.ensembl.org/index.html) (strain identifier Desulfovibrio_desulfuricans_subsp_desulfuricans_str_atcc_27774.ASM2212v1); iii > *assignment of reads per genes* in each sample by using feature Counts (from the subread suite)^70^. Finally the detection of “differentially expressed genes” (DEG) relied on the R library edgeR^71^. We applied a threshold of 0.05 on the adjusted p-value, and of 1.5 on the fold change to select for DEG. Table 1 gives the number of differentially expressed genes for each comparison between growth conditions (Sulfate + NO *vs* Sulfate; Nitrate + NO *vs* Nitrate; Nitrate *vs* Sulfate; Nitrate + NO *vs* Sulfate + NO).

A detailed technical report of all the statistical treatments (data exploration, differential analysis, cross-species functional enrichment) is available on the supporting website.

### Analysis of regulatory sequences

The software suite Regulatory Sequence Analysis Tools (RSAT, http://rsat.eu/) was used to analyse regions upstream of genes in the reference genome Desulfovibrio_desulfuricans_ATCC_27774_uid59213, which was downloaded from NCBI.

The tool *retrieve-seq* was used to retrieve promoter sequences up to 400 bp upstream from position −1 relative to the translation start codon up to the nearest neighbouring gene.

The HcpR1 (Ddes_0528) binding sequence TGTGA-N6-TCACA, where N6 denotes a succession of 6 undefined nucleotides, was used to construct a seed matrix by setting an arbitrary weight of 8 on each residue of the binding site, and 0 on other nucleotides (Supplementary Fig. S2). The upstream sequences of all genes were scanned with *matrix-scan* to collect sequences matching this seed matrix with a p-value < 10^−4^. The 125 resulting sites were aligned to build a second-generation matrix with the tool *convert-matrix*. This second-generation matrix was used to rescan all promoters with *matrix-scan*, and sites with a p-value < 10^−4^ were identified.

## Electronic supplementary material

Supplementary data
