# Supplementary material for: Coordinated response of the Desulfovibrio desulfuricans 27774 transcriptome to nitrate, nitrite and nitric oxide
Source: Sci Rep. 2017 Nov 24;7:16228. doi: 10.1038/s41598-017-16403-4 (PMC5701242; doi:10.1038/s41598-017-16403-4)
Supplement: Supplementary file 1 — Supplementary data [file 41598_2017_16403_MOESM1_ESM.doc]

**Supplementary information**

**Coordinated response of the *Desulfovibrio desulfuricans* 27774 transcriptome to nitrate, nitrite and nitric oxide**

**Ian T. Cadby^1^, Matthew Faulkner^1**^, Jeanne Cheneby^2^, Justine Long^2^, Jacques van Helden^2^, Alain Dolla^3^ and Jeffrey A. Cole^1*^,**

*^1^School of Biosciences, University of Birmingham, Birmingham B15 2TT, UK*

*^2^Aix Marseille Univ, INSERM, TAGC*, *UMR_S 1090, 163, Avenue de Luminy, 13288 Marseille, France*

*^3^Aix Marseille Univ, CNRS, LCB, Marseille, France*

**Supplementary table S1. Candidate binding sites for HcpR1 and NrfR proteins upstream of the *nap* gene clusters of *Desulfovibrio* and related bacteria**.

| **Bacterium** | **Candidate HcpR1 sites** | **Position*** | **Candidate NrfR sites** | **Position*** | ***nap* cluster** |
| --- | --- | --- | --- | --- | --- |
| ***D. desulfuricans* 27774** | TATGAAACTGGTCATA | -186 | GTCATGTTTTTATGAAACTGGTCATATTTTTATGAATG | -186 | *CMADGH* |
|  | TGTGATGTTTTTCTCA | -143 |  |  |  |
|  | AGTGAGTAATATCACA | -124 |  |  |  |
| ***Desulfovibrio 6_1_46AFAA*** | TGTGAGCTTTTTCTCT | -141 | GTCATGATATTATGACGGTTGTCAGATTTTTCTGAATC | -184 | *CMADGH* |
|  | TATGACGGTTGTCAGA | -184 |  |  |  |
| ***Bilophila sp. 4_1_30*** | TGTGAAAAAAAGCACT | -32 | GTCATCTTTTCATGACAATCAGTCAGAAAAAGATGACT | -152 | *CMADGH* |
| ***Desulfovibrio sp. 3_1_syn3*** | TATGACGGTTGTCAGA | -302 | GTCATGATATTATGACGGTTGTCAGATTTTTCTGAATA | -301 | *CMADGH* |
|  | TGTGAGCTTTTTCTCT | -259 |  |  |  |
| ***Desulfovibrio sp. A2*** | TGTGCAATCTTTCACA | -43 | TTCAGAAATAGCTGACAGCATTTCAGAAAAACATGACT | -149 | *CMADGH* |
| ***Bilophila sp.* 3_1_6** | TGTGAAAAAAAGCACT | +7 | GTCATCTTTTCATGACAATCAGTCAGAAAAAGATGACT | -113 | *CMADGH* |
| ***Deferribacter desulfuricans SSM1*** | TGTGGCGACATTCATA | -312 | GTCAACCCCATAATAGAGCCATTGAATACCACCACAGG | -128 | *MADGH* |
|  | TGTGGAGGTGCTCATG | -5 |  |  |  |
| ***Desulfurispirillum indicum S5*** | TGTCTTCTCCTTCACA | -189 | ATCAACCTGAATACCTTTGATTTCAAGAGCGAATGCAC | -250 | *MADGH* |
|  | TGTGAATAAATTCACC | +16 |  |  |  |

**Supplementary table S2: Analysis of RNA-seq data. The cDNA libraries from three independent biological replicates were prepared from cells grown in the presence of sulfate or nitrate as electron acceptor in the presence or absence of NO.**

| Sample | | |  | Million reads |  |
| --- | --- | --- | --- | --- | --- |
| Electron acceptor | NO | Replicate | Before mapping | Aligned reads | Assigned to genes |
| Nitrate | - | N1 | 53.1 | 51.7 | 39.2 |
|  | - | N2 | 38.2 | 36.2 | 30.5 |
|  | - | N4 | 26.6 | 24.7 | 22.8 |
| Nitrate | + | NN2 | 36.1 | 33.2 | 23.6 |
|  | + | NN4 | 22.5 | 19.3 | 16.0 |
|  | + | NN5 | 40.1 | 38.1 | 35.1 |
| Sulfate | - | S1 | 36.8 | 35.1 | 20.5 |
|  | - | S4 | 33.6 | 32.0 | 29.7 |
|  | - | S5 | 35.7 | 8.7 | 7.1 |
| Sulfate | + | SN1 | 36.8 | 34.3 | 23.5 |
|  | + | SN2 | 35.0 | 30.6 | 22.4 |
|  | + | SN5 | 33.1 | 30.7 | 26.7 |

**Table S3** : Genes differentially expressed during growth with nitrate compared to sulfate as electron acceptor.

| Gene Id | Name | Description | log_2_FC^1^ | padj^2^ | COG |
| --- | --- | --- | --- | --- | --- |
| Ddes_0001 |  | hypothetical protein | -1.23 | 0.048 |  |
| Ddes_0016 |  | hypothetical protein | -1.19 | 0.0381 |  |
| Ddes_0018 |  | response regulator receiver protein | -2.72 | 4.93E-07 | T |
| Ddes_0019 |  | DSBA oxidoreductase | -1.79 | 0.00767 | O |
| Ddes_0021 |  | NLP/P60 protein | 1.63 | 0.00401 | M |
| Ddes_0025 |  | NAD-dependent epimerase/dehydratase | -1.15 | 0.0125 | G |
| Ddes_0027 |  | Precorrin-8X methylmutase CbiC/CobH | 1.54 | 0.0101 | H |
| Ddes_0031 |  | Holliday junction resolvase YqgF | -1.57 | 0.0115 | L |
| Ddes_0032 |  | tryptophanyl-tRNA synthetase | -3.11 | 3.04E-08 | J |
| Ddes_0040 |  | ArsR family transcriptional regulator | -2.69 | 0.000172 | Q |
| Ddes_0042 |  | hypothetical protein | -1.92 | 0.00277 |  |
| Ddes_0045 |  | glutamate decarboxylase | 2.95 | 0.00219 | E |
| Ddes_0055 | *rpsO* | 30S ribosomal protein S15 | 1.41 | 0.0125 | J |
| Ddes_0056 |  | tRNA pseudouridine synthase B | 1.91 | 0.0168 | J |
| Ddes_0058 |  | hypothetical protein | -2.71 | 3.36E-05 | S |
| Ddes_0065 |  | cyclophilin type peptidyl-prolyl cis-trans isomerase | -1.08 | 0.0306 | O |
| Ddes_0067 |  | YceI family protein | 1.86 | 0.0199 | S |
| Ddes_0069 |  | family 2 glycosyl transferase | 1.65 | 0.0416 | R |
| Ddes_0070 |  | thiamine-monophosphate kinase | 1.87 | 0.000929 | H |
| Ddes_0073 |  | putative rRNA methylase | 1.56 | 0.0178 | Q |
| Ddes_0076 |  | Dyp-type peroxidase family protein | -1.87 | 0.00041 | P |
| Ddes_0077 |  | DEAD/DEAH box helicase domain-containing protein | 1.43 | 0.0156 | R |
| Ddes_0081 | *nrfA* | Nitrite reductase | 4.04 | 0.000366 | P |
| Ddes_0082 | *nrfH* | NapC/NirT cytochrome c family protein | 3.57 | 0.000664 | C |
| Ddes_0083 |  | Hpt sensor hybrid histidine kinase | -1.73 | 0.00422 | T |
| Ddes_0087 |  | histone family protein DNA-binding protein | -1.46 | 0.00719 | L |
| Ddes_0097 |  | ErfK/YbiS/YcfS/YnhG family protein | 1.91 | 7.69E-05 | S |
| Ddes_0101 |  | isocitrate/isopropylmalate dehydrogenase | 1.34 | 0.00983 | C |
| Ddes_0111 |  | small hypothetical protein | -4.01 | 2.43E-11 |  |
| Ddes_0112 |  | sarcosine reductase | -3.07 | 3.39E-07 |  |
| Ddes_0113 |  | selenoprotein B, glycine/betaine/sarcosine/D-proline reductase family | -2.89 | 5.39E-07 |  |
| Ddes_0114 |  | thioredoxin reductase | -3.06 | 8.77E-08 | O |
| Ddes_0115 |  | betaine reductase | -2.17 | 5.69E-06 | I |
| Ddes_0116 |  | glycine reductase | -1.21 | 0.0209 | I |
| Ddes_0122 |  | adenylylsulfate kinase-like protein | 1.75 | 0.00229 | P |
| Ddes_0134 |  | methyl-accepting chemotaxis sensory transducer | -1.96 | 0.00659 | N |
| Ddes_0135 |  | pyruvate ferredoxin/flavodoxin oxidoreductase | 1.37 | 0.0146 | C |
| Ddes_0142 |  | hypothetical protein | 1.8 | 0.00164 |  |
| Ddes_0152 |  | glycine cleavage system H protein | -1.97 | 0.00115 | E |
| Ddes_0153 |  | OsmC family protein | -2.67 | 8.11E-07 | O |
| Ddes_0154 |  | Na+/solute symporter | -1.2 | 0.0284 | E |
| Ddes_0165 |  | hypothetical protein | -1.27 | 0.00925 | R |
| Ddes_0173 |  | NAD(P)(+) transhydrogenase | 2.51 | 0.00966 | C |
| Ddes_0174 |  | putative NADPH-NAD transhydrogenase alpha subunit | 1.74 | 0.0199 | C |
| Ddes_0175 |  | NAD(P)(+) transhydrogenase | 1.62 | 0.016 | C |
| Ddes_0178 |  | Sel1 domain-containing protein | 1.29 | 0.0207 | R |
| Ddes_0179 |  | hypothetical protein | 1.37 | 0.0287 |  |
| Ddes_0180 |  | membrane bound O-acyl transferase MBOAT family protein | 1.89 | 0.00411 | M |
| Ddes_0184 |  | hypothetical protein | 2.34 | 0.0212 |  |
| Ddes_0185 |  | hypothetical protein | 2.36 | 0.000663 |  |
| Ddes_0203 |  | YbaK/prolyl-tRNA synthetase associated protein | -2.03 | 2.15E-05 | S |
| Ddes_0204 |  | hypothetical protein | -2.54 | 1.93E-05 | R |
| Ddes_0205 |  | hypothetical protein | -3.68 | 3.04E-06 |  |
| Ddes_0210 |  | LysR family transcriptional regulator | -1.09 | 0.0302 | K |
| Ddes_0214 |  | NAD(P)(+) transhydrogenase | 2.33 | 0.007 | C |
| Ddes_0215 |  | putative NADPH-NAD transhydrogenase alpha subunit | 1.61 | 0.0424 | C |
| Ddes_0216 |  | NAD(P)(+) transhydrogenase | 1.38 | 0.0184 | C |
| Ddes_0217 |  | hypothetical protein | 1.87 | 0.0111 |  |
| Ddes_0219 |  | Fis family PAS modulated sigma54 specific transcriptional regulator | -3.44 | 2.87E-07 | K |
| Ddes_0226 |  | hypothetical protein | -3.3 | 1.77E-11 | S |
| Ddes_0227 |  | hypothetical protein | -1.61 | 0.00193 |  |
| Ddes_0233 |  | hypothetical protein | -2.3 | 0.00181 |  |
| Ddes_0265 |  | hypothetical protein | -1.66 | 0.00143 |  |
| Ddes_0266 |  | hypothetical protein | -2.41 | 0.000946 | K |
| Ddes_0272 |  | DNA-cytosine methyltransferase | -1.05 | 0.0377 | L |
| Ddes_0273 |  | hypothetical protein | -1.16 | 0.0359 | V |
| Ddes_0275 |  | transposase IS3/IS911 family protein | -2.31 | 1.61E-06 | L |
| Ddes_0278 |  | hypothetical protein | -1.52 | 0.0466 |  |
| Ddes_0280 |  | hypothetical protein | 2.34 | 0.00219 |  |
| Ddes_0283 |  | Mg chelatase, subunit ChlI | 1.3 | 0.0381 | O |
| Ddes_0284 |  | Serine-type D-Ala-D-Ala carboxypeptidase | 2.41 | 0.000267 | M |
| Ddes_0288 |  | delta-aminolevulinic acid dehydratase | 1.14 | 0.042 | H |
| Ddes_0289 |  | Radical SAM domain-containing protein | 2.18 | 0.00114 | R |
| Ddes_0294 |  | hypothetical protein | 1.46 | 0.0468 | K |
| Ddes_0300 |  | methyl-accepting chemotaxis sensory transducer | -1.32 | 0.0115 | N |
| Ddes_0302 |  | hypothetical protein | 1.61 | 0.0262 |  |
| Ddes_0303 |  | hypothetical protein | -1.87 | 0.0114 |  |
| Ddes_0305 |  | hypothetical protein | 2.8 | 0.000309 | O |
| Ddes_0311 |  | hypothetical protein | 2.46 | 3.91E-05 |  |
| Ddes_0312 |  | glycosyl transferase family 9 | 1.75 | 0.00129 | M |
| Ddes_0317 |  | hypothetical protein | -2.76 | 4.13E-05 |  |
| Ddes_0323 |  | flagellar basal body-associated protein FliL | -2.55 | 9.80E-06 | N |
| Ddes_0326 |  | Cobyrinic acid ac-diamide synthase | 1.43 | 0.0124 | D |
| Ddes_0327 |  | GTP-binding signal recognition particle SRP54 G- domain-containing protein | 1.94 | 0.00267 | N |
| Ddes_0329 |  | XRE family transcriptional regulator | -1.38 | 0.0473 | K |
| Ddes_0331 | *flhA* | flagellar biosynthesis protein FlhA | 1.48 | 0.0128 | N |
| Ddes_0333 |  | major facilitator superfamily protein | 1.79 | 0.00336 | E |
| Ddes_0334 |  | Prephenate dehydrogenase | 1.85 | 0.00164 | E |
| Ddes_0335 |  | 3-phosphoshikimate 1-carboxyvinyltransferase | 2.29 | 4.16E-05 | E |
| Ddes_0336 |  | chorismate mutase | 2.47 | 3.53E-06 | E |
| Ddes_0337 |  | 3-dehydroquinate synthase | 1.87 | 0.00292 | E |
| Ddes_0339 |  | D-cysteine desulfhydrase | -2.81 | 0.0115 | E |
| Ddes_0340 |  | Na+/H+ antiporter NhaC | -1.57 | 0.0102 | C |
| Ddes_0341 |  | GntR family transcriptional regulator | -1.42 | 0.00608 | K |
| Ddes_0343 |  | hypothetical protein | -0.983 | 0.049 |  |
| Ddes_0347 | *tsf* | Elongation factor Ts | 1.06 | 0.0473 | J |
| Ddes_0350 |  | phosphoglycerate mutase | -2.74 | 1.35E-05 | G |
| Ddes_0353 |  | hypothetical protein | 1.61 | 0.00258 |  |
| Ddes_0355 |  | hypothetical protein | -2.07 | 0.000217 |  |
| Ddes_0365 |  | hypothetical protein | -1.14 | 0.0218 |  |
| Ddes_0369 |  | hypothetical protein | 1.57 | 0.0282 | C |
| Ddes_0375 |  | flagellar motor switch protein FliN | 1.1 | 0.0459 | N |
| Ddes_0376 |  | flagellar biosynthesis protein FliO | 1.34 | 0.0279 | N |
| Ddes_0378 |  | flagellar biosynthetic protein FliQ | 1.57 | 0.0317 | N |
| Ddes_0382 |  | carbon-monoxide dehydrogenase, catalytic subunit | -1.46 | 0.0195 | C |
| Ddes_0383 |  | putative Crp/Fnr family transcriptional regulator | -2.46 | 0.00219 | T |
| Ddes_0388 |  | tol-pal system protein YbgF | 1.82 | 0.00361 | S |
| Ddes_0391 |  | hypothetical protein | 2.36 | 0.00292 | N |
| Ddes_0392 | *flgG* | flagellar basal body rod protein FlgG | 2.12 | 0.00139 | N |
| Ddes_0393 |  | flagella basal body P-ring formation protein FlgA | 1.92 | 0.00122 | N |
| Ddes_0394 | *flgH* | flagellar basal body L-ring protein | 1.64 | 0.00788 | N |
| Ddes_0395 |  | hypothetical protein | -1.07 | 0.0315 | L |
| Ddes_0396 |  | glycosyltransferase-like protein | -1.22 | 0.0137 | M |
| Ddes_0397 |  | group 1 glycosyl transferase | -1.68 | 0.000956 | M |
| Ddes_0399 |  | family 2 glycosyl transferase | -1.24 | 0.0178 | M |
| Ddes_0407 |  | transferase hexapeptide repeat containing protein | -1.73 | 0.000399 | R |
| Ddes_0408 |  | contains MurG-like glycosyltransferase domain | -3.48 | 4.73E-07 |  |
| Ddes_0409 |  | hypothetical protein | -1.38 | 0.0221 |  |
| Ddes_0410 |  | ABC transporter-like protein | -2.48 | 0.00015 | V |
| Ddes_0419 |  | 4-vinyl reductase 4VR | -1.75 | 0.000451 | R |
| Ddes_0421 |  | diguanylate cyclase/phosphodiesterase | -1.6 | 0.00216 | T |
| Ddes_0430 |  | AraC family transcriptional regulator | -2.79 | 5.31E-07 | K |
| Ddes_0431 |  | transposase IS3/IS911 family protein | -2.22 | 5.03E-06 | L |
| Ddes_0435 |  | Cobyrinic acid ac-diamide synthase | -2.11 | 0.00105 | D |
| Ddes_0436 |  | hypothetical protein | -2.05 | 0.000759 |  |
| Ddes_0437 |  | hypothetical protein | -1.92 | 8.04E-05 |  |
| Ddes_0442 |  | hypothetical protein | 1.74 | 0.000967 |  |
| Ddes_0443 |  | indolepyruvate ferredoxin oxidoreductase subunit alpha | 1.2 | 0.0335 | C |
| Ddes_0444 |  | Indolepyruvate ferredoxin oxidoreductase | 1.46 | 0.0298 | C |
| Ddes_0446 |  | metal dependent phosphohydrolase | -3.19 | 6.08E-07 | J |
| Ddes_0457 |  | hypothetical protein | 1.38 | 0.0162 |  |
| Ddes_0477 |  | dihydrodipicolinate reductase | -3.51 | 2.65E-06 | E |
| Ddes_0478 |  | MltA-interacting MipA family protein | -1.65 | 0.000684 | M |
| Ddes_0488 | *rpmI* | 50S ribosomal protein L35 | -1.25 | 0.0359 | J |
| Ddes_0489 | *infC* | translation initiation factor IF-3 | -2.45 | 3.09E-06 | J |
| Ddes_0493 |  | RNP-1 like RNA-binding protein | -2.89 | 9.73E-09 | R |
| Ddes_0496 |  | hypothetical protein | -2.15 | 1.35E-05 |  |
| Ddes_0513 |  | rare lipoprotein A | -2.03 | 0.0087 | M |
| Ddes_0524 |  | HPP family protein | 1.71 | 0.0148 | T |
| Ddes_0525 |  | 4Fe-4S iron-sulfur protein | 1.94 | 0.000299 | C |
| Ddes_0526 | *wrbA* | FMN-binding protein | 2.6 | 1.67E-06 | R |
| Ddes_0527 | *nimA* | flavodoxin family protein | 2.05 | 9.34E-05 | C |
| Ddes_0528 | *hcpR1* | Crp/Fnr family transcription factor HcpR1 | 1.85 | 0.00109 | T |
| Ddes_0534 |  | Mammalian cell entry related domain-containing protein | -1.95 | 0.00497 | Q |
| Ddes_0543 |  | hypothetical protein | -1.15 | 0.014 | S |
| Ddes_0545 |  | 2-hydroxyglutaryl-CoA dehydratase D-component | 3.97 | 0.000399 | E |
| Ddes_0546 |  | CoA-substrate-specific enzyme activase | 2.45 | 0.00143 | I |
| Ddes_0548 |  | 4-amino-4-deoxy-L-arabinose transferase-like protein | 1.37 | 0.00921 | M |
| Ddes_0549 |  | hypothetical protein | 1.22 | 0.0231 |  |
| Ddes_0556 |  | formate dehydrogenase subunit beta | 2.36 | 0.000878 | C |
| Ddes_0558 |  | cytochrome c class III | 1.35 | 0.0318 |  |
| Ddes_0560 |  | iojap-like protein | -1.27 | 0.0191 | S |
| Ddes_0567 |  | winged helix family two component transcriptional regulator | 1.22 | 0.0372 | K |
| Ddes_0569 |  | phosphate ABC transporter ATPase | 1.6 | 0.0359 | P |
| Ddes_0580 |  | chaperone DnaJ domain-containing protein | -2.28 | 0.00172 | O |
| Ddes_0589 |  | family 3 extracellular solute-binding protein | 1.71 | 0.0381 | E |
| Ddes_0592 |  | tRNA (5-methylaminomethyl-2-thiouridylate)-methyltransferase | 1.51 | 0.0164 | J |
| Ddes_0593 |  | hypothetical protein | -1.77 | 0.00017 |  |
| Ddes_0594 |  | sodium/calcium exchanger membrane protein | -1.8 | 0.000131 | P |
| Ddes_0596 |  | hypothetical protein | 1.51 | 0.00773 |  |
| Ddes_0602 |  | peroxiredoxin | -2.7 | 0.000543 | O |
| Ddes_0604 |  | hypothetical protein | -1.78 | 0.0159 |  |
| Ddes_0609 |  | N-acetylmuramoyl-L-alanine amidase | 1.69 | 0.0303 | M |
| Ddes_0612 |  | ABC transporter-like protein | -1.3 | 0.0113 | V |
| Ddes_0614 | *napC* | NapC/NirT cytochrome c domain-containing protein | 3.67 | 1.92E-10 | C |
| Ddes_0615 | *napM* | hypothetical protein | 3.74 | 1.26E-10 | O |
| Ddes_0616 | *napA* | Periplasmic nitrate reductase | 3.25 | 3.24E-07 | C |
| Ddes_0617 | *napD* | hypothetical protein | 2.83 | 0.000187 |  |
| Ddes_0619 | *napH* | quinol dehydrogenase membrane component | 1.98 | 0.0186 | C |
| Ddes_0622 |  | UPF0210 protein Ddes_0622 | 1.58 | 0.00353 | S |
| Ddes_0624 |  | hypothetical protein | 1.16 | 0.0417 |  |
| Ddes_0625 |  | hypothetical protein | 3.53 | 3.71E-06 | L |
| Ddes_0628 |  | thiamine biosynthesis protein ThiC | 1.12 | 0.0268 | H |
| Ddes_0637 |  | putative PAS/PAC sensor protein | 1.17 | 0.0386 | T |
| Ddes_0641 |  | Alanine--glyoxylate transaminase | 2.71 | 0.00178 | E |
| Ddes_0644 |  | FeoA family protein | -3.05 | 4.73E-07 | P |
| Ddes_0645 |  | FeoA family protein | -3.92 | 1.96E-08 | P |
| Ddes_0646 |  | small GTP-binding protein | -1.95 | 0.000582 | P |
| Ddes_0647 |  | hypothetical protein | -2.64 | 1.28E-05 |  |
| Ddes_0648 |  | hypothetical protein | -2.66 | 2.29E-08 | P |
| Ddes_0654 |  | integral membrane protein MviN | 1.86 | 0.0148 | R |
| Ddes_0661 | *rplD* | 50S ribosomal protein L4 | 1.52 | 0.0202 | J |
| Ddes_0662 | *rplW* | 50S ribosomal protein L23 | 1.96 | 0.000905 | J |
| Ddes_0663 | *rplB* | 50S ribosomal protein L2 | 2.27 | 0.00146 | J |
| Ddes_0664 | *rpsS* | 30S ribosomal protein S19 | 1.41 | 0.0418 | J |
| Ddes_0665 | *rplV* | 50S ribosomal protein L22 | 1.34 | 0.0359 | J |
| Ddes_0677 | *rpsE* | 30S ribosomal protein S5 | 1.3 | 0.0309 | J |
| Ddes_0680 | *secY* | preprotein translocase subunit SecY | -1.06 | 0.0262 | U |
| Ddes_0688 |  | short chain dehydrogenase | -1.68 | 0.0012 | I |
| Ddes_0692 | *clpX* | ATP-dependent protease ATP-binding subunit ClpX | -1.16 | 0.0335 | O |
| Ddes_0693 |  | hypothetical protein | -2.57 | 0.000663 |  |
| Ddes_0695 |  | hypothetical protein | 2.27 | 0.000168 |  |
| Ddes_0697 |  | hypothetical protein | 1.65 | 0.00118 | S |
| Ddes_0698 |  | flagellar export protein FliJ | 1.85 | 0.00118 | N |
| Ddes_0702 |  | hypothetical protein: part of a putative gene transfer island | -2.32 | 3.12E-06 |  |
| Ddes_0703 |  | hypothetical protein: part of a putative gene transfer island | -1.51 | 0.0306 |  |
| Ddes_0704 |  | hypothetical protein: part of a putative gene transfer island | -2.17 | 0.000136 |  |
| Ddes_0705 |  | hypothetical protein: part of a putative gene transfer island | -2.76 | 9.35E-06 |  |
| Ddes_0706 |  | hypothetical protein: part of a putative gene transfer island | -4.68 | 3.26E-14 |  |
| Ddes_0707 |  | hypothetical protein: part of a putative gene transfer island | -3.91 | 1.04E-11 |  |
| Ddes_0708 |  | GCN5-like N-acetyltransferase: part of a putative gene transfer island | -4.29 | 1.28E-12 | K |
| Ddes_0709 |  | hypothetical protein: part of a putative gene transfer island | -5.08 | 2.83E-13 |  |
| Ddes_0710 |  | hypothetical protein: part of a putative gene transfer island | -5.33 | 2.21E-24 |  |
| Ddes_0711 |  | hypothetical protein: part of a putative gene transfer island | -4.75 | 4.24E-12 | U |
| Ddes_0712 |  | hypothetical protein: part of a putative gene transfer island | -5.37 | 1.72E-20 |  |
| Ddes_0713 |  | hypothetical protein: part of a putative gene transfer island | -6.66 | 1.99E-14 |  |
| Ddes_0714 |  | hypothetical protein: part of a putative gene transfer island | -6.47 | 1.96E-13 |  |
| Ddes_0715 |  | hypothetical protein: part of a putative gene transfer island | -6.14 | 1.27E-16 |  |
| Ddes_0716 |  | hypothetical protein: part of a putative gene transfer island | -7.27 | 1.85E-22 |  |
| Ddes_0717 |  | hypothetical protein: part of a putative gene transfer island | -6.33 | 6.03E-17 |  |
| Ddes_0718 |  | hypothetical protein: part of a putative gene transfer island | -5.85 | 1.37E-15 |  |
| Ddes_0719 |  | hypothetical protein: part of a putative gene transfer island | -5.43 | 1.55E-14 |  |
| Ddes_0720 |  | hypothetical protein: part of a putative gene transfer island | -5.96 | 8.59E-17 |  |
| Ddes_0721 |  | hypothetical protein: part of a putative gene transfer island | -6.44 | 2.04E-19 |  |
| Ddes_0722 |  | hypothetical protein: part of a putative gene transfer island | -5.81 | 1.26E-15 |  |
| Ddes_0723 |  | hypothetical protein: part of a putative gene transfer island | -6.35 | 5.85E-18 | R |
| Ddes_0725 |  | hypothetical protein: part of a putative gene transfer island | -5.15 | 1.85E-22 |  |
| Ddes_0726 |  | putative phage repressor: part of a putative gene transfer island | -2.63 | 0.000138 | K |
| Ddes_0729 |  | hypothetical protein: part of a putative gene transfer island | 1.38 | 0.0348 | S |
| Ddes_0732 |  | RND family efflux transporter MFP subunit | 1.5 | 0.0208 | M |
| Ddes_0733 |  | acriflavin resistance protein | 1.3 | 0.0387 | V |
| Ddes_0743 | *argS* | Arginine--tRNA ligase | 1.23 | 0.0148 | J |
| Ddes_0749 |  | MarR family transcriptional regulator | -2.39 | 1.65E-06 | K |
| Ddes_0751 |  | carboxylyase-like protein | 1.48 | 0.00661 | H |
| Ddes_0759 |  | hypothetical protein | 2.47 | 0.00343 | R |
| Ddes_0761 |  | histidinol-phosphate aminotransferase | -1.24 | 0.00689 | E |
| Ddes_0763 |  | hypothetical protein | 2.3 | 0.00547 |  |
| Ddes_0767 |  | hypothetical protein | 1.99 | 0.0243 | S |
| Ddes_0768 |  | PAS/PAC sensor signal transduction histidine kinase | -2.98 | 5.03E-06 | T |
| Ddes_0772 |  | hypothetical protein | -1.89 | 0.00855 | E |
| Ddes_0777 |  | respiratory-chain NADH dehydrogenase, subunit 1 | 1.74 | 0.0369 | C |
| Ddes_0778 |  | NADH dehydrogenase (quinone) | 2.06 | 0.000331 | C |
| Ddes_0781 |  | phosphate binding protein | 2.36 | 0.0305 | P |
| Ddes_0786 |  | glycine cleavage system T protein | 2.63 | 0.00375 | E |
| Ddes_0787 |  | glycine cleavage system H protein | 2.99 | 0.000285 | E |
| Ddes_0789 |  | glycine dehydrogenase subunit 2 | 1.87 | 0.0372 | E |
| Ddes_0792 |  | metallophosphoesterase | -1.51 | 0.0243 | R |
| Ddes_0794 |  | hypothetical protein | 1.69 | 0.00653 | S |
| Ddes_0796 |  | transport system permease | 1.95 | 0.0309 | P |
| Ddes_0819 |  | putative phage repressor | -4.22 | 7.29E-09 | K |
| Ddes_0821 |  | methyl-accepting chemotaxis sensory transducer | -1.62 | 0.0198 | N |
| Ddes_0822 |  | glycine betaine ABC transporter substrate-binding protein | 2.94 | 0.000142 | E |
| Ddes_0829 |  | formate dehydrogenase family accessory protein FdhD | -1.99 | 0.00529 | C |
| Ddes_0838 |  | hypothetical protein | 1.81 | 0.0409 | F |
| Ddes_0843 |  | BadM/Rrf2 family transcriptional regulator | 3.81 | 2.39E-05 | K |
| Ddes_0844 |  | response regulator receiver protein | 3.55 | 3.15E-06 | T |
| Ddes_0847 |  | hypothetical protein | -3.22 | 1.22E-10 |  |
| Ddes_0849 |  | SEC-C motif domain-containing protein | -1.07 | 0.0285 | S |
| Ddes_0851 |  | glucose-6-phosphate isomerase | 2.25 | 3.90E-05 | G |
| Ddes_0865 |  | hypothetical protein | 2.29 | 3.91E-05 |  |
| Ddes_0869 |  | Membrane-bound lytic murein transglycosylase B-like protein | 1.16 | 0.0344 | M |
| Ddes_0870 |  | hypothetical protein | 2.35 | 0.00651 |  |
| Ddes_0877 |  | glucose-1-phosphate cytidylyltransferase | -1.17 | 0.0185 | J |
| Ddes_0883 |  | hypothetical protein | 2.22 | 0.00802 | C |
| Ddes_0884 |  | hypothetical protein | 2.47 | 6.82E-05 |  |
| Ddes_0888 |  | hypothetical protein | -1.04 | 0.0456 | R |
| Ddes_0897 |  | Rubrerythrin | -4.3 | 1.40E-08 | C |
| Ddes_0898 |  | hypothetical protein | -1.46 | 0.0068 | S |
| Ddes_0903 |  | hypothetical protein | 1.53 | 0.0113 |  |
| Ddes_0905 |  | hypothetical protein | 2.17 | 0.0125 | R |
| Ddes_0907 |  | HesB/YadR/YfhF-family protein | -1.85 | 0.0292 |  |
| Ddes_0908 |  | HesB/YadR/YfhF-family protein | -1.78 | 0.0302 |  |
| Ddes_0913 |  | hypothetical protein | 1.34 | 0.0138 | S |
| Ddes_0921 |  | sulfite reductase, dissimilatory-type alpha subunit | 1.95 | 0.0393 | C |
| Ddes_0925 |  | Cse3 family CRISPR-associated protein | -1.73 | 0.00015 |  |
| Ddes_0930 |  | hypothetical protein | 2.07 | 0.00898 | S |
| Ddes_0931 |  | hypothetical protein | 1.57 | 0.024 | C |
| Ddes_0932 |  | hypothetical protein | 3.22 | 9.13E-05 | T |
| Ddes_0934 |  | hypothetical protein | -1.4 | 0.0251 | R |
| Ddes_0935 |  | hypothetical protein | -1.53 | 0.0183 |  |
| Ddes_0937 |  | hypothetical protein | -3.62 | 2.26E-06 | L |
| Ddes_0942 |  | methyl-accepting chemotaxis sensory transducer | -1.4 | 0.0138 | N |
| Ddes_0943 |  | transposase IS3/IS911 family protein | -1.89 | 0.000515 | L |
| Ddes_0947 |  | hypothetical protein | -1.63 | 0.00217 |  |
| Ddes_0951 | *proS* | Proline--tRNA ligase | 1.12 | 0.0315 | J |
| Ddes_0953 |  | peptidase M23 | 1.13 | 0.0336 | M |
| Ddes_0955 |  | polyprenyl synthetase | 1.09 | 0.0466 | H |
| Ddes_0957 |  | tRNA(Ile)-lysidine synthetase | 1.68 | 0.0222 | D |
| Ddes_0963 |  | hypothetical protein | 1.21 | 0.028 | S |
| Ddes_0966 |  | DNA protecting protein DprA | 1.21 | 0.0362 | L |
| Ddes_0967 |  | integrase family protein | 1.63 | 0.0153 | L |
| Ddes_0974 |  | RNA polymerase sigma-54 subunit RpoN | -1.76 | 0.0356 | K |
| Ddes_0981 |  | hypothetical protein | 2.55 | 3.20E-05 |  |
| Ddes_0983 |  | amino acid permease-associated protein | -2.03 | 0.00135 | E |
| Ddes_1000 |  | Multidrug resistance efflux pump-like protein | 2.9 | 2.76E-07 | V |
| Ddes_1001 |  | hypothetical protein | 1.58 | 0.00375 | S |
| Ddes_1004 |  | ribosomal L11 methyltransferase | 1.22 | 0.0468 | J |
| Ddes_1011 |  | Redoxin domain-containing protein | -1.75 | 0.028 | O |
| Ddes_1012 | *hslU* | ATP-dependent protease ATPase subunit HslU | -1.51 | 0.00199 | O |
| Ddes_1016 |  | LicD family protein | -1.46 | 0.0146 | M |
| Ddes_1020 |  | aspartate racemase | -1.06 | 0.0282 | M |
| Ddes_1026 |  | hypothetical protein | -2.51 | 0.000993 |  |
| Ddes_1028 |  | flagellin domain-containing protein | 2.91 | 0.00107 | N |
| Ddes_1053 |  | GrpE protein | -2.41 | 0.00292 | O |
| Ddes_1063 | *rpsI* | 30S ribosomal protein S9 | 1.5 | 0.00948 | J |
| Ddes_1076 |  | Fe-S cluster assembly protein NifU | 2.04 | 0.00797 | C |
| Ddes_1077 |  | BadM/Rrf2 family transcriptional regulator | 1.85 | 0.00728 | K |
| Ddes_1091 | *ksgA* | dimethyladenosine transferase | 2.42 | 0.0451 | J |
| Ddes_1092 |  | elongation factor G | 1.41 | 0.0102 | J |
| Ddes_1094 | *argH* | Argininosuccinate lyase | 1.59 | 0.00226 | E |
| Ddes_1101 |  | 4Fe-4S ferredoxin iron-sulfur binding domain-containing protein | 1.54 | 0.00542 | C |
| Ddes_1104 |  | MraZ protein | -3.33 | 7.65E-09 | S |
| Ddes_1105 | *rsmH* | Ribosomal RNA small subunit methyltransferase H | 1.63 | 0.0313 | M |
| Ddes_1107 |  | peptidoglycan glycosyltransferase | 1.17 | 0.0416 | M |
| Ddes_1108 |  | UDP-N-acetylmuramyl-tripeptide synthetase | 2.05 | 0.000199 | M |
| Ddes_1117 |  | cell division protein FtsA | 1.36 | 0.00948 | D |
| Ddes_1118 |  | cell division protein FtsZ | 2.43 | 9.34E-05 | D |
| Ddes_1146 |  | Fis family transcriptional regulator | -2.29 | 6.78E-06 | K |
| Ddes_1153 |  | transglutaminase family protein cysteine peptidase BTLCP | -1.06 | 0.0405 | S |
| Ddes_1158 |  | nitroreductase | -3.17 | 1.30E-05 | C |
| Ddes_1160 |  | methylated-DNA/protein-cysteine methyltransferase | -1.33 | 0.0162 | L |
| Ddes_1161 |  | AraC family transcriptional regulator | -3.14 | 3.20E-13 | F |
| Ddes_1162 |  | GntR family transcriptional regulator | -1.37 | 0.0052 | E |
| Ddes_1164 |  | Cupin 2 barrel domain-containing protein | -2.61 | 0.000341 | G |
| Ddes_1176 |  | Triose-phosphate isomerase | 2.39 | 5.59E-05 | G |
| Ddes_1177 | *secG* | preprotein translocase subunit SecG | 1.85 | 0.00109 | U |
| Ddes_1183 |  | histidine triad (HIT) protein | -1.27 | 0.0262 | F |
| Ddes_1191 |  | Mrp protein | -1.1 | 0.0264 | D |
| Ddes_1192 |  | CDP-diacylglycerol/glycerol-3-phosphate 3-phosphatidyltransferase | -1.84 | 7.39E-05 | I |
| Ddes_1198 | *gcp* | Probable tRNA threonylcarbamoyladenosine biosynthesis protein Gcp | -1.19 | 0.00957 | O |
| Ddes_1209 |  | phosphoglucomutase | -1.75 | 0.0141 | G |
| Ddes_1219 |  | hypothetical protein | -1.68 | 0.0269 | L |
| Ddes_1224 |  | XRE family transcriptional regulator | -1.12 | 0.0492 | K |
| Ddes_1229 |  | integrase family protein | -2.71 | 0.000236 | L |
| Ddes_1232 |  | metal dependent phosphohydrolase | -1.44 | 0.00421 |  |
| Ddes_1235 |  | teichoic acid biosynthesis-like protein | -1.24 | 0.00584 | C |
| Ddes_1238 |  | Respiratory-chain NADH dehydrogenase domain 51 kDa subunit | 2.33 | 0.00194 | C |
| Ddes_1239 |  | NQR2 and RnfD family protein | 2.61 | 1.99E-05 | C |
| Ddes_1240 |  | FMN-binding domain-containing protein | 2.79 | 5.70E-05 | C |
| Ddes_1241 |  | electron transport complex RsxE subunit | 2.78 | 1.11E-05 | C |
| Ddes_1242 |  | RnfABCDGE type electron transport complex subunit A | 3.42 | 7.71E-09 | C |
| Ddes_1243 |  | ferredoxin | 3.94 | 1.87E-08 | C |
| Ddes_1244 |  | ApbE family lipoprotein | 2.96 | 2.86E-06 | H |
| Ddes_1247 |  | hypothetical protein | -4.74 | 4.62E-11 |  |
| Ddes_1248 |  | hypothetical protein | -2.6 | 0.000169 |  |
| Ddes_1253 |  | AMP-dependent synthetase and ligase | -2 | 4.45E-05 | I |
| Ddes_1254 | *dtd* | D-tyrosyl-tRNA(Tyr) deacylase | -1.1 | 0.0466 | J |
| Ddes_1259 |  | flagellar hook-length control protein | 2.3 | 0.000148 | N |
| Ddes_1260 |  | flagellar hook capping protein | 2.29 | 1.99E-05 | N |
| Ddes_1261 |  | hypothetical protein | 2.33 | 0.00443 | N |
| Ddes_1263 |  | transposase IS3/IS911 family protein | -1.74 | 0.00219 | L |
| Ddes_1264 |  | ArsR family transcriptional regulator | 2.42 | 0.0161 | K |
| Ddes_1270 |  | hypothetical protein | -2.21 | 2.17E-05 |  |
| Ddes_1271 |  | hypothetical protein | -1.68 | 0.0108 |  |
| Ddes_1276 |  | response regulator receiver protein | -1.12 | 0.0372 | T |
| Ddes_1282 | *gatB* | Aspartyl/glutamyl-tRNA(Asn/Gln) amidotransferase subunit B | 1.08 | 0.0377 | J |
| Ddes_1289 |  | TetR family transcriptional regulator | -2.85 | 0.000154 | K |
| Ddes_1291 |  | Na+/solute symporter | 1.53 | 0.00683 | E |
| Ddes_1297 |  | hypothetical protein | -2.77 | 2.49E-07 |  |
| Ddes_1305 |  | Fis family sigma-54 specific transcriptional regulator | -1.16 | 0.0331 | T |
| Ddes_1315 |  | hypothetical protein | 1.15 | 0.0438 | R |
| Ddes_1316 |  | PpkA-like protein | 1.28 | 0.0195 |  |
| Ddes_1324 |  | hypothetical protein | -1.63 | 0.0208 | R |
| Ddes_1336 |  | beta-ketoacyl synthase | -1.26 | 0.0381 | Q |
| Ddes_1343 |  | TonB-dependent receptor | -2.9 | 4.13E-05 | P |
| Ddes_1344 |  | Biopolymer transport protein ExbD/TolR | -4.13 | 1.59E-13 | U |
| Ddes_1345 |  | isochorismate synthase | -2.21 | 0.000518 | H |
| Ddes_1346 |  | chorismate mutase-like protein | -4.14 | 2.99E-10 | E |
| Ddes_1360 |  | acetaldehyde dehydrogenase | 1.5 | 0.00515 | C |
| Ddes_1361 |  | microcompartments protein | 2.06 | 0.00428 | C |
| Ddes_1378 |  | HEAT domain containing protein | 1.68 | 0.0126 | C |
| Ddes_1379 |  | CheR-type MCP methyltransferase | 1.28 | 0.0202 | N |
| Ddes_1383 |  | signal transduction histidine kinase CheA | -1.55 | 0.0368 | N |
| Ddes_1385 |  | Rhomboid family protein | -1.65 | 0.000566 | R |
| Ddes_1386 | *rd2* | Rubredoxin-2 | 1.79 | 0.0173 | C |
| Ddes_1388 |  | Fur family ferric uptake regulator | -3.22 | 1.99E-05 | P |
| Ddes_1411 |  | cyclic nucleotide-binding protein | -1.52 | 0.0291 | T |
| Ddes_1419 |  | hypothetical protein | -3.01 | 6.63E-06 |  |
| Ddes_1422 |  | metal dependent phosphohydrolase | -1.49 | 0.00308 | R |
| Ddes_1427 |  | hypothetical protein | -2.38 | 3.04E-08 |  |
| Ddes_1428 |  | leucyl/phenylalanyl-tRNA/protein transferase | -1.14 | 0.0246 | O |
| Ddes_1430 |  | ATP-dependent Clp protease adaptor protein ClpS | -2.38 | 3.48E-07 | S |
| Ddes_1431 |  | adenylate cyclase | -2.12 | 4.68E-06 | F |
| Ddes_1439 |  | hypothetical protein | 2.28 | 1.35E-05 |  |
| Ddes_1442 |  | homocysteine S-methyltransferase | 1.17 | 0.0222 | E |
| Ddes_1443 |  | putative RNA polymerase, sigma 70 family subunit | -1.37 | 0.0407 | K |
| Ddes_1455 |  | CarD family transcriptional regulator | -2.19 | 0.00306 | K |
| Ddes_1459 |  | MotA/TolQ/ExbB proton channel | -3.56 | 4.54E-06 | U |
| Ddes_1460 |  | Biopolymer transport protein ExbD/TolR | -2.71 | 3.18E-07 | U |
| Ddes_1461 |  | TonB family protein | -1.59 | 0.00966 | M |
| Ddes_1462 |  | hypothetical protein | -1.72 | 0.000258 |  |
| Ddes_1468 |  | O-acetylhomoserine/O-acetylserine sulfhydrylase | 2.68 | 0.00128 | E |
| Ddes_1472 |  | hypothetical protein | 1.6 | 0.0238 |  |
| Ddes_1476 |  | pseudouridine synthase | -1.57 | 0.0292 | J |
| Ddes_1484 |  | hypothetical protein | -3.21 | 2.61E-11 |  |
| Ddes_1494 |  | Catalase | -4.42 | 1.09E-07 | P |
| Ddes_1499 |  | hypothetical protein | -1.35 | 0.00686 | R |
| Ddes_1501 |  | small GTP-binding protein | 2.82 | 8.11E-07 | R |
| Ddes_1502 |  | Fe-Fe hydrogenase: ferredoxin hydrogenase | 4.58 | 5.22E-06 |  |
| Ddes_1503 |  | hydrogenase, Fe-only | 3.76 | 0.000694 | R |
| Ddes_1504 |  | biotin synthase | 2.77 | 0.00011 | H |
| Ddes_1507 |  | sodium:dicarboxylate symporter | 1.87 | 0.00428 | C |
| Ddes_1515 |  | periplasmic binding protein | 1.89 | 0.000678 | P |
| Ddes_1518 |  | hypothetical protein | 1.81 | 0.00561 | P |
| Ddes_1519 | *glpC* | sn-glycerol-3-phosphate dehydrogenase subunit C | 1.13 | 0.0416 | C |
| Ddes_1520 |  | anaerobic glycerol-3-phosphate dehydrogenase subunit B | 1.16 | 0.0478 | E |
| Ddes_1526 |  | hypothetical protein | -1.85 | 0.000228 |  |
| Ddes_1527 |  | anion transporter | 2.16 | 0.00787 | P |
| Ddes_1528 |  | hydro-lyase, Fe-S type, tartrate/fumarate subfamily subunit alpha | 3.77 | 8.22E-08 | C |
| Ddes_1529 |  | hydro-lyase, Fe-S type, tartrate/fumarate subfamily subunit beta | 3.36 | 1.11E-07 | C |
| Ddes_1530 |  | fumarate reductase respiratory complex transmembrane subunit | 3.55 | 6.00E-07 | C |
| Ddes_1531 |  | fumarate reductase flavoprotein subunit | 3.94 | 2.77E-06 | C |
| Ddes_1533 |  | CBS domain containing membrane protein | 2.38 | 0.00435 | R |
| Ddes_1534 |  | malate dehydrogenase | 2.43 | 0.00897 | C |
| Ddes_1536 |  | (p)ppGpp synthetase I SpoT/RelA | -1.53 | 0.00636 | K |
| Ddes_1538 |  | family 5 extracellular solute-binding protein | -1.7 | 0.00225 | E |
| Ddes_1545 |  | molybdopterin oxidoreductase | 1.47 | 0.0125 | C |
| Ddes_1547 | *groS* | 10 kDa chaperonin | -2.01 | 0.0101 | O |
| Ddes_1548 | *groL* | 60 kDa chaperonin | -2.65 | 0.00346 | O |
| Ddes_1549 |  | UV-endonuclease UvdE | -1.13 | 0.0356 | L |
| Ddes_1550 |  | TetR family transcriptional regulator | -1.28 | 0.0203 | K |
| Ddes_1558 |  | PhoH family protein | -1.78 | 0.000145 | T |
| Ddes_1559 |  | hypothetical protein | 1.92 | 0.00219 |  |
| Ddes_1562 |  | polar amino acid ABC transporter inner membrane subunit | -1.7 | 0.00665 | E |
| Ddes_1570 |  | hypothetical protein | 1.95 | 0.00039 | N |
| Ddes_1573 |  | flagellar M-ring protein FliF | 1.94 | 0.00314 | N |
| Ddes_1574 |  | flagellar hook-basal body complex subunit FliE | 2.12 | 0.00198 | N |
| Ddes_1575 |  | flagellar basal-body rod protein FlgC | 2.63 | 0.000137 | N |
| Ddes_1576 | *flgB* | flagellar basal body rod protein FlgB | 2.52 | 0.00181 | N |
| Ddes_1579 |  | phosphoadenosine phosphosulfate reductase | -2.83 | 1.37E-05 | R |
| Ddes_1585 |  | Fe^++^ transport protein similar to FeoB | -1.41 | 0.00266 | P |
| Ddes_1586 |  | hypothetical protein | -5.01 | 1.46E-06 |  |
| Ddes_1587 | *trpA* | Tryptophan synthase alpha chain | 4.2 | 1.61E-06 | E |
| Ddes_1588 |  | tryptophan synthase subunit beta | 3.98 | 3.36E-05 | E |
| Ddes_1589 | *trpF* | N-(5'-phosphoribosyl)anthranilate isomerase | 2.87 | 6.69E-05 | E |
| Ddes_1590 |  | Indole-3-glycerol-phosphate synthase | 3.15 | 3.32E-07 | E |
| Ddes_1591 |  | anthranilate phosphoribosyltransferase | 2.43 | 0.000678 | E |
| Ddes_1592 |  | Anthranilate synthase | 2.05 | 0.0104 | E |
| Ddes_1594 |  | FKBP-type peptidylprolyl isomerase | 1.25 | 0.0262 | O |
| Ddes_1595 |  | diaminopimelate decarboxylase | -1.6 | 0.0255 | E |
| Ddes_1605 |  | Molecular chaperone-like protein | -1.5 | 0.00508 | O |
| Ddes_1606 |  | Sel1 domain-containing protein | -1.66 | 0.0335 | R |
| Ddes_1607 |  | hypothetical protein | -1.85 | 0.00259 | O |
| Ddes_1611 | *rpmF* | 50S ribosomal protein L32 | 1.51 | 0.0162 | J |
| Ddes_1624 |  | DNA polymerase III delta | -2.82 | 9.80E-06 | L |
| Ddes_1625 |  | DNA repair protein RadC | -1.83 | 0.0429 | L |
| Ddes_1631 |  | NusG antitermination factor | -1.26 | 0.0163 | K |
| Ddes_1632 | *rplK* | 50S ribosomal protein L11 | -2.15 | 8.41E-06 | J |
| Ddes_1633 | *rplA* | 50S ribosomal protein L1 | -1.6 | 0.00164 | J |
| Ddes_1638 |  | hypothetical protein | -2.79 | 2.44E-05 |  |
| Ddes_1639 |  | hypothetical protein | -3.91 | 3.48E-08 |  |
| Ddes_1640 |  | putative lipoprotein | -2.41 | 0.0332 |  |
| Ddes_1642 |  | hypothetical protein | -3.29 | 0.00232 |  |
| Ddes_1643 |  | Sigma 54 interacting domain-containing protein | -1.52 | 0.000961 | T |
| Ddes_1644 |  | pyruvate phosphate dikinase | -1.55 | 0.00152 | G |
| Ddes_1645 |  | major facilitator superfamily protein | -1.71 | 0.000185 | E |
| Ddes_1648 |  | hypothetical protein | -1.74 | 0.0253 | S |
| Ddes_1651 |  | hypothetical protein | -0.969 | 0.0441 | S |
| Ddes_1652 |  | hypothetical protein | -2.22 | 0.000105 |  |
| Ddes_1660 |  | NADH-quinone oxidoreductase subunit I | 1.39 | 0.0483 | C |
| Ddes_1661 |  | NADH dehydrogenase (quinone) | -3 | 3.03E-08 | C |
| Ddes_1666 |  | 4Fe-4S ferredoxin iron-sulfur binding domain-containing protein | 1.34 | 0.0207 | C |
| Ddes_1668 |  | 4Fe-4S ferredoxin iron-sulfur binding domain-containing protein | 4.16 | 6.11E-09 | C |
| Ddes_1669 |  | NADH-ubiquinone oxidoreductase chain 49kDa | 2.1 | 0.00277 | C |
| Ddes_1670 |  | NADH dehydrogenase (ubiquinone) 30 kDa subunit | 1.9 | 0.00472 | C |
| Ddes_1671 |  | NADH ubiquinone oxidoreductase 20 kDa subunit | 3.23 | 2.99E-06 | C |
| Ddes_1672 |  | respiratory-chain NADH dehydrogenase subunit 1 | 3.29 | 5.82E-08 | C |
| Ddes_1673 |  | NADH dehydrogenase (quinone) | 2.1 | 0.00198 | C |
| Ddes_1674 |  | putative diguanylate cyclase | -1.59 | 0.00164 | N |
| Ddes_1692 |  | hypothetical protein | 2.3 | 0.000408 | S |
| Ddes_1695 |  | hypothetical protein | -2.17 | 0.00332 | L |
| Ddes_1701 |  | hypothetical protein | 1.43 | 0.0341 |  |
| Ddes_1702 |  | hypothetical protein | -2.56 | 5.06E-06 |  |
| Ddes_1703 |  | magnesium-translocating P-type ATPase | -1.73 | 0.00502 | P |
| Ddes_1704 |  | hypothetical protein | -2.1 | 0.0132 |  |
| Ddes_1705 |  | thiamine pyrophosphate TPP binding domain-containing protein | -2.4 | 0.000154 | E |
| Ddes_1708 |  | hypothetical protein | -1.34 | 0.00983 | H |
| Ddes_1719 |  | GntR family transcriptional regulator | -2.3 | 9.09E-05 | K |
| Ddes_1728 |  | hypothetical protein | -2.22 | 1.32E-05 | S |
| Ddes_1729 |  | ATPase | -4.53 | 1.40E-08 | R |
| Ddes_1730 |  | diguanylate cyclase and serine/threonine protein kinase with TPR repeats | 2.4 | 0.000186 | R |
| Ddes_1731 |  | beta-lactamase domain-containing protein | -1.61 | 0.00508 | R |
| Ddes_1732 |  | NADPH-dependent FMN reductase | 1.2 | 0.0302 | R |
| Ddes_1734 |  | hypothetical protein | -1.34 | 0.0298 | P |
| Ddes_1737 |  | thioesterase superfamily protein | -1.92 | 0.000638 | Q |
| Ddes_1738 |  | hypothetical protein | -1.75 | 0.00277 | T |
| Ddes_1745 |  | hypothetical protein | 1.21 | 0.0381 | S |
| Ddes_1747 |  | Maf-like protein | 1.69 | 0.0125 | D |
| Ddes_1750 |  | FeoA family protein | -5.22 | 1.96E-13 | P |
| Ddes_1757 |  | Undecaprenyl-phosphate galactose phosphotransferase, WbaP | -2.18 | 1.77E-06 | M |
| Ddes_1758 |  | family 3 extracellular solute-binding protein | 2.1 | 0.00626 | E |
| Ddes_1762 |  | diguanylate cyclase/phosphodiesterase | -1.36 | 0.0185 | T |
| Ddes_1766 | *folE2* | GTP cyclohydrolase folE2 | -2.07 | 0.000519 | S |
| Ddes_1774 |  | sulfur transfer protein ThiS | -1.36 | 0.0256 | H |
| Ddes_1775 |  | aldehyde ferredoxin oxidoreductase | -2.39 | 0.000466 | C |
| Ddes_1781 |  | LacI family transcriptional regulator | -1.19 | 0.0169 | K |
| Ddes_1782 |  | major facilitator superfamily protein | -2.31 | 4.20E-06 | E |
| Ddes_1790 | *rplS* | 50S ribosomal protein L19 | 1.33 | 0.0251 | J |
| Ddes_1793 |  | Uroporphyrin-III C/tetrapyrrole (Corrin/Porphyrin) methyltransferase | -1.67 | 0.000346 | R |
| Ddes_1807 |  | twin-arginine translocation protein, TatB subunit | 2.34 | 0.0019 | U |
| Ddes_1810 |  | putative lipoprotein | 1.34 | 0.036 |  |
| Ddes_1812 |  | small multidrug resistance protein | -2.32 | 8.49E-07 | P |
| Ddes_1815 |  | phosphoglycerate mutase | 1.88 | 0.0252 | G |
| Ddes_1816 |  | metal dependent phosphohydrolase | -1.51 | 0.0134 | R |
| Ddes_1817 |  | coenzyme F390 synthetase | 1.45 | 0.0348 | H |
| Ddes_1824 |  | molybdopterin binding domain-containing protein | -1.55 | 0.0174 | H |
| Ddes_1827 | *hcpR2* | Crp/Fnr family transcriptional regulator | -1.69 | 9.31E-05 | T |
| Ddes_1829 | *hcp* | hybrid cluster protein: NO reductase | 3.19 | 0.00121 | C |
| Ddes_1831 |  | cyclopropane fatty acyl phospholipid synthase | -1.88 | 0.00387 | M |
| Ddes_1834 |  | hypothetical protein | 1.78 | 0.00538 | J |
| Ddes_1835 |  | iron-sulfur cluster-binding protein | 1.82 | 0.00188 | R |
| Ddes_1838 |  | hypothetical protein | 1.51 | 0.0281 | S |
| Ddes_1845 | *murA* | UDP-N-acetylglucosamine 1-carboxyvinyltransferase | 2.05 | 0.000975 | M |
| Ddes_1846 |  | FAD-dependent pyridine nucleotide-disulfide oxidoreductase | 3.99 | 1.53E-05 | R |
| Ddes_1847 |  | hypothetical protein | 4.06 | 4.70E-07 | S |
| Ddes_1848 |  | hypothetical protein | -1.62 | 0.000819 |  |
| Ddes_1852 |  | hypothetical protein | 1.8 | 0.00812 |  |
| Ddes_1863 |  | hypothetical protein | -2.18 | 0.0153 | S |
| Ddes_1864 |  | Dinitrogenase iron-molybdenum cofactor biosynthesis protein | -5.35 | 1.68E-15 | S |
| Ddes_1865 |  | Cobyrinic acid ac-diamide synthase | -3.9 | 1.91E-09 | C |
| Ddes_1866 |  | 4Fe-4S ferredoxin iron-sulfur binding domain-containing protein | -3.63 | 1.57E-06 | C |
| Ddes_1867 |  | Dinitrogenase iron-molybdenum cofactor biosynthesis protein | -5.31 | 5.82E-10 | S |
| Ddes_1873 |  | hypothetical protein | -2.21 | 1.35E-05 | S |
| Ddes_1874 |  | amidohydrolase | 1.15 | 0.0348 | F |
| Ddes_1875 |  | Hpt protein | -1.09 | 0.0226 | T |
| Ddes_1876 |  | heavy metal translocating P-type ATPase | -2.58 | 0.000249 | P |
| Ddes_1877 |  | Heavy metal transport/detoxification protein | -2.93 | 3.39E-07 | P |
| Ddes_1881 |  | respiratory-chain NADH dehydrogenase subunit 1 | -1.49 | 0.00287 | C |
| Ddes_1882 |  | NADH ubiquinone oxidoreductase 20 kDa subunit | -1.67 | 0.00223 | C |
| Ddes_1884 |  | NADH dehydrogenase (ubiquinone) 30 kDa subunit | -1.85 | 8.89E-05 | C |
| Ddes_1886 |  | hydrogenase expression/synthesis HypA | -1.56 | 0.0156 | R |
| Ddes_1890 |  | N-acetylneuraminate synthase | -2.46 | 0.000392 | M |
| Ddes_1897 |  | SNO glutamine amidotransferase | -1.37 | 0.0161 | H |
| Ddes_1898 | *pdxS* | Pyridoxal biosynthesis lyase pdxS | -1.49 | 0.0129 | H |
| Ddes_1899 |  | hypothetical protein | 1.14 | 0.03 |  |
| Ddes_1902 |  | hypothetical protein | 2.4 | 0.00625 | S |
| Ddes_1904 | *ilvH* | acetolactate synthase 3 regulatory subunit | 1.64 | 0.00966 | E |
| Ddes_1905 | *ilvC* | Ketol-acid reductoisomerase | 1.55 | 0.0466 | E |
| Ddes_1915 |  | hypothetical protein | 1.57 | 0.0339 | S |
| Ddes_1919 |  | tRNA methyltransferase complex GCD14 subunit | -1.42 | 0.00292 | J |
| Ddes_1921 |  | hypothetical protein | -1.55 | 0.0354 |  |
| Ddes_1922 |  | ABC transporter-like protein | -1.33 | 0.0492 | O |
| Ddes_1925 |  | putative ArsR family transcriptional regulator | -3.33 | 2.86E-06 | K |
| Ddes_1926 |  | type 11 methyltransferase | -1.87 | 0.000364 | Q |
| Ddes_1927 |  | RNP-1 like RNA-binding protein | -1.62 | 0.0086 | R |
| Ddes_1935 |  | hypothetical protein | -1.65 | 0.000142 |  |
| Ddes_1937 | *lnt* | apolipoprotein N-acyltransferase | 1.06 | 0.0381 | M |
| Ddes_1939 |  | putative diguanylate cyclase | 2.2 | 0.000121 | T |
| Ddes_1942 |  | dihydrodipicolinate synthase | 1.96 | 0.0187 | E |
| Ddes_1943 |  | iron-containing alcohol dehydrogenase | 1.97 | 0.0264 | C |
| Ddes_1946 | *thiH* | thiamine biosynthesis protein ThiH | -2.53 | 0.000878 | H |
| Ddes_1951 |  | Flavodoxin | -4.19 | 8.11E-07 | C |
| Ddes_1961 |  | hypothetical protein | -1.42 | 0.00661 | P |
| Ddes_1973 | *aroK* | shikimate kinase | -2.34 | 2.49E-07 | E |
| Ddes_1977 |  | hypothetical protein | -2.32 | 0.00219 | E |
| Ddes_1979 |  | precorrin-6y C5,15-methyltransferase subunit CbiE | 1.37 | 0.00473 | H |
| Ddes_1981 |  | aldehyde dehydrogenase | -2.54 | 7.73E-05 | C |
| Ddes_1985 |  | histidinol phosphate phosphatase protein | -1.88 | 0.0115 | E |
| Ddes_1988 |  | hypothetical protein | -0.983 | 0.0353 | R |
| Ddes_1992 |  | hypothetical protein | -1.95 | 0.000463 | S |
| Ddes_1995 |  | hypothetical protein | -2.1 | 0.00292 |  |
| Ddes_1996 |  | outer membrane autotransporter barrel domain-containing protein | 1.08 | 0.0377 | U |
| Ddes_2001 |  | flagellar hook-associated protein FlgK | 1.35 | 0.0466 | N |
| Ddes_2002 |  | FlgN family protein | 3.81 | 5.06E-06 |  |
| Ddes_2003 |  | flagellar protein FlgJ | 2.34 | 0.00022 | M |
| Ddes_2004 | *flgI* | Flagellar P-ring protein | 2.18 | 0.000532 | N |
| Ddes_2005 |  | anti-sigma-28 factor FlgM | -1.32 | 0.00966 | K |
| Ddes_2006 |  | hypothetical protein | -2.48 | 7.82E-06 |  |
| Ddes_2008 |  | methylenetetrahydrofolate reductase (NAD(P)H) | -3.25 | 1.11E-05 | E |
| Ddes_2015 |  | cobalamin (vitamin B12) biosynthesis CbiG protein | 2.16 | 0.000515 | H |
| Ddes_2030 |  | Spermine synthase | -1.32 | 0.0125 | R |
| Ddes_2036 | *miaA* | tRNA dimethylallyltransferase | 1.74 | 0.0179 | J |
| Ddes_2039 |  | 4Fe-4S ferredoxin iron-sulfur binding domain-containing protein | 1.92 | 0.0109 | C |
| Ddes_2040 |  | polysulfide reductase NrfD | 1.43 | 0.0458 | E |
| Ddes_2041 |  | hypothetical protein | 2.54 | 0.000409 |  |
| Ddes_2044 |  | hypothetical protein | -0.975 | 0.0486 |  |
| Ddes_2046 |  | transferase hexapeptide repeat containing protein | 1.13 | 0.05 | R |
| Ddes_2047 |  | 3-deoxy-manno-octulosonate cytidylyltransferase | 1.91 | 0.00292 | M |
| Ddes_2050 |  | MoeA domain-containing protein domain I and II | 1.61 | 0.0431 | H |
| Ddes_2051 |  | iron-containing alcohol dehydrogenase | 2.97 | 0.00419 | C |
| Ddes_2052 |  | TOBE domain-containing protein | -2.02 | 0.00752 | R |
| Ddes_2054 |  | peptidase M48 Ste24p | -1.77 | 0.0149 | O |
| Ddes_2057 |  | hypothetical protein | 1.47 | 0.0483 |  |
| Ddes_2058 |  | group 1 glycosyl transferase | 1.59 | 0.0146 | M |
| Ddes_2066 |  | hypothetical protein | 1.68 | 0.0169 |  |
| Ddes_2075 | *lipB* | Octanoyltransferase | -1.6 | 0.0101 | H |
| Ddes_2079 |  | lytic transglycosylase | 1.37 | 0.00941 | M |
| Ddes_2088 |  | hypothetical protein | -1.36 | 0.0496 | R |
| Ddes_2090 |  | Rhodanese domain-containing protein | 1.29 | 0.0415 | P |
| Ddes_2098 |  | heterodisulfide reductase subunit C | 1.76 | 0.00772 | C |
| Ddes_2099 |  | CoB-CoM heterodisulfide reductase | 1.31 | 0.0271 | C |
| Ddes_2104 |  | hypothetical protein | 2.23 | 0.00587 | S |
| Ddes_2105 |  | 460 residue transmembrane protein | 2.52 | 3.73E-05 | V |
| Ddes_2106 |  | ABC transporter-like protein | 2.63 | 9.09E-05 | V |
| Ddes_2130 |  | adenylylsulfate reductase subunit beta | -2.6 | 0.00259 | C |
| Ddes_2133 |  | hypothetical protein | -2.29 | 0.00343 |  |
| Ddes_2136 |  | 3,4-dihydroxy-2-butanone 4-phosphate synthase | -1.67 | 0.000364 | H |
| Ddes_2142 |  | Hydantoinase/oxoprolinase | 1.24 | 0.0303 | E |
| Ddes_2143 |  | branched-chain amino acid aminotransferase | 1.74 | 0.013 | E |
| Ddes_2144 |  | DNA polymerase III subunits gamma/tau | 1.53 | 0.00217 | L |
| Ddes_2147 |  | recombination protein RecR | 1.84 | 0.0381 | L |
| Ddes_2151 | *dnaK* | Chaperone protein DnaK | -1.72 | 0.04 | O |
| Ddes_2153 |  | glutamyl-tRNA(Gln) amidotransferase subunit C | -2.11 | 0.00099 | J |
| Ddes_2156 |  | sodium:dicarboxylate symporter | -1.11 | 0.0341 | C |
| Ddes_2157 |  | hypothetical protein | -1.98 | 0.00216 |  |
| Ddes_2158 |  | small multidrug resistance protein | -2.53 | 1.78E-06 | P |
| Ddes_2159 |  | peptidase M23 | -1.61 | 0.0195 | M |
| Ddes_2162 |  | chaperone protein DnaJ | -1.35 | 0.0466 | O |
| Ddes_2163 |  | carbon starvation protein CstA | -2.31 | 0.000364 | T |
| Ddes_2164 |  | hypothetical protein | -2.58 | 1.41E-06 |  |
| Ddes_2165 |  | thiamine biosynthesis protein ThiS | 1.71 | 0.00626 | H |
| Ddes_2166 | *thiG* | thiazole synthase | 1.92 | 0.000288 | H |
| Ddes_2174 |  | DegT/DnrJ/EryC1/StrS aminotransferase | 1.64 | 0.0113 | M |
| Ddes_2175 |  | CheW protein | -1.64 | 0.00067 | N |
| Ddes_2177 |  | cobyric acid synthase CobQ | 1.15 | 0.0406 | H |
| Ddes_2180 |  | OmpA/MotB domain-containing protein | -1.62 | 0.0189 | N |
| Ddes_2184 |  | H+transporting two-sector ATPase B/B' subunit | 1.4 | 0.031 | C |
| Ddes_2185 | *atpH* | ATP synthase subunit delta | 2.09 | 0.00297 | C |
| Ddes_2186 | *atpA* | ATP synthase subunit alpha | 1.69 | 0.0381 | C |
| Ddes_2202 |  | NAD-dependent epimerase/dehydratase | 1.86 | 0.000454 | G |
| Ddes_2205 |  | oxygen-independent coproporphyrinogen III oxidase | 2.35 | 2.99E-05 | H |
| Ddes_2219 |  | family 2 glycosyl transferase | -1.74 | 0.000139 | M |
| Ddes_2223 |  | hypothetical protein | -3.03 | 2.27E-09 |  |
| Ddes_2227 |  | methyl-accepting chemotaxis sensory transducer | -1.5 | 0.00354 | N |
| Ddes_2228 |  | transposase IS3/IS911 family protein | -3.22 | 5.87E-11 | L |
| Ddes_2230 |  | hypothetical protein | -1.59 | 0.000825 |  |
| Ddes_2233 |  | XRE family transcriptional regulator | -3.13 | 9.32E-08 | K |
| Ddes_2234 |  | hypothetical protein | -1.57 | 0.0466 |  |
| Ddes_2235 |  | hypothetical protein | -1.9 | 0.0194 |  |
| Ddes_2242 |  | BamHI-like type II restriction endonuclease | -1.55 | 0.00176 |  |
| Ddes_2243 |  | DNA methylase N-4/N-6 domain-containing protein | -1.5 | 0.00988 | L |
| Ddes_2249 | *rpmA* | 50S ribosomal protein L27 | 1.07 | 0.048 | J |
| Ddes_2252 |  | hypothetical protein | -2.74 | 1.95E-08 | S |
| Ddes_2253 |  | hypothetical protein | -2.51 | 1.77E-06 |  |
| Ddes_2257 |  | redox-sensing transcriptional repressor Rex | -1.42 | 0.0424 | R |
| Ddes_2261 |  | diguanylate cyclase | -2.42 | 0.000173 | T |
| Ddes_2267 |  | DNA polymerase III subunit delta' | -2.05 | 0.0025 | L |
| Ddes_2268 |  | PAS/PAC sensor-containing diguanylate cyclase | -1.38 | 0.0368 | E |
| Ddes_2269 |  | hypothetical protein | 1.75 | 0.00325 |  |
| Ddes_2274 |  | aspartate-semialdehyde dehydrogenase | 1.61 | 0.0413 | E |
| Ddes_2275 |  | class IV aminotransferase | 1.44 | 0.0113 | E |
| Ddes_2280 |  | flagellin domain-containing protein | -1.58 | 0.0471 | N |
| Ddes_2285 |  | hypothetical protein | -1.41 | 0.0146 |  |
| Ddes_2286 | *panC* | Pantothenate synthetase | -2.25 | 0.00216 | H |
| Ddes_2287 |  | S-adenosylmethionine synthetase | -1.26 | 0.0298 | H |
| Ddes_2296 |  | hypothetical protein | -1.59 | 0.0151 |  |
| Ddes_2300 |  | hypothetical protein | 1.4 | 0.0227 | R |
| Ddes_2305 |  | hypothetical protein | 1.39 | 0.0486 |  |
| Ddes_2306 |  | putative lipoprotein | 1.49 | 0.00931 |  |
| Ddes_2308 |  | Antibiotic biosynthesis monooxygenase | 1.78 | 0.00135 | S |
| Ddes_2311 |  | hypothetical protein | 1.22 | 0.0246 |  |
| Ddes_2314 |  | homoserine dehydrogenase | 1.29 | 0.00988 | E |
| Ddes_2325 |  | Fmu (Sun) domain-containing protein | 1.4 | 0.0105 | J |
| Ddes_2330 |  | hypothetical protein | 1.24 | 0.0372 |  |
| Ddes_2334 |  | anaerobic cobalt chelatase | 3.12 | 5.59E-05 | H |
| Ddes_2338 |  | Phenylacetate--CoA ligase | 1.48 | 0.00346 | H |
| Ddes_2339 |  | Asparaginase | 1.56 | 0.00261 | E |
| Ddes_2345 |  | Fis family sigma-54 specific transcriptional regulator | -1.04 | 0.0353 | T |
| Ddes_2352 |  | RNA-directed DNA polymerase | -1.72 | 0.0191 | L |
| Ddes_2353 |  | hypothetical protein | -1.79 | 0.00231 |  |
| Ddes_2355 |  | transposase IS3/IS911 family protein | -2.29 | 1.46E-06 | L |
| Ddes_2358 |  | filamentation induced by cAMP protein Fic | -1.99 | 0.00198 | S |
| Ddes_2359 |  | integrase family protein | -3.06 | 3.73E-06 | L |
| Ddes_2367 |  | heme exporter protein CcmA | 1.49 | 0.0154 | V |
| Ddes_2370 |  | diguanylate cyclase | -1.48 | 0.0401 | T |
| Ddes_R0007 |  | 16S ribosomal RNA | -2.29 | 0.0381 |  |
| Ddes_R0023 |  | tRNA-Leu | -1.37 | 0.0356 |  |
| Ddes_R0032 | *ssrA* |  | 2.99 | 0.00579 |  |
| Ddes_R0041 |  | 16S ribosomal RNA | -2.3 | 0.0381 |  |
| Ddes_R0049 |  | tRNA-Thr | -1.66 | 0.0139 |  |
| Ddes_R0050 |  | tRNA-Tyr | -2 | 0.000285 |  |
| Ddes_R0051 |  | tRNA-Gly | -1.71 | 0.00219 |  |
| Ddes_R0059 |  | 16S ribosomal RNA | -2.3 | 0.0381 |  |
| EBG00001164127 |  |  | -1.65 | 0.00519 |  |
| EBG00001164141 |  |  | -2.3 | 0.0381 |  |
| EBG00001164144 |  |  | 7.16 | 0.0138 |  |
| EBG00001164161 |  |  | -2.31 | 0.0381 |  |
| EBG00001164166 |  |  | 2.22 | 0.0339 |  |
| EBG00001164170 |  |  | -1.7 | 0.00287 |  |
| EBG00001164173 |  |  | -1.91 | 0.000105 |  |
| EBG00001164181 |  |  | -2.31 | 0.0381 |  |
| EBG00001164187 |  |  | 2.98 | 0.00577 |  |

^1^. FC : fold change.

^2^. Pdaj : adjusted p-value

**Table S4.** Enrichment of differentially expressed genes for functional classes of COGs, in the comparison between sulfate and nitrate as electron acceptors. **DEG nb**: number of differentially expressed genes assigned to the COG class; **DEG %:** percent of the DEG belonging to the COG class; **Genome nb**: total number of genes assigned to the COG class; **Genome %**: percentage of the genes assigned to the considered COG class among the 2,332 genes assigned to at least one COG class; **expected DEG nb**: number of DEGs expected by chance in the COG class; **DEG ratio**: ratio between DEG nb and expected DEG nb; **Pval:** nominal P-value of the hypergeometric test, corresponding to the risk of false positive (FPR) for a single test; **FWER**: family-wise error rate, i.e. multiple testing correction indicating the probability of observing at least one false positive among all COG classes, given the nominal P-value; **FDR**: False Discovery Rate, i.e. the expected proportion of false positive among the DEG considered as significant.

Table S5. Genes differentially expressed in the presence of exogenous NO with nitrate as electron acceptor.

| Gene Id | Name | Description | log_2_FC^1^ | Padj^2^ | COG |
| --- | --- | --- | --- | --- | --- |
| Ddes_0204 |  | hypothetical protein | 1.77 | 0.0315 | R |
| Ddes_0205 |  | hypothetical protein | 2.75 | 0.00866 |  |
| Ddes_0286 |  | putative AsnC family transcriptional regulator | 1.5 | 0.0107 | K |
| Ddes_0288 |  | delta-aminolevulinic acid dehydratase | 1.68 | 0.00714 | H |
| Ddes_0524 |  | HPP family protein | 1.83 | 0.00323 | T |
| Ddes_0525 |  | 4Fe-4S ferredoxin iron-sulfur binding domain-containing protein | 1.8 | 0.0269 | C |
| Ddes_0526 |  | pyridoxamine 5'-phosphate oxidase-like protein | 2.57 | 0.0041 | R |
| Ddes_0527 |  | flavodoxin family protein | 2.11 | 0.00347 | C |
| Ddes_0528 | *hcpR1* | Crp/Fnr family transcriptional regulator | 2.54 | 0.00504 | T |
| Ddes_0648 |  | hypothetical protein | 1.65 | 0.0107 | P |
| Ddes_1164 |  | Cupin 2 barrel domain-containing protein | 3.03 | 0.00323 | G |
| Ddes_1165 |  | hypothetical protein | 3.38 | 0.00323 | S |
| Ddes_1248 |  | hypothetical protein | 2.23 | 0.0269 |  |
| Ddes_1336 |  | beta-ketoacyl synthase | 2.21 | 0.0408 | Q |
| Ddes_1501 |  | small GTP-binding protein | -1.83 | 0.00714 | R |
| Ddes_1581 |  | hypothetical protein | -2.65 | 0.0429 |  |
| Ddes_1729 |  | ATPase | 2.21 | 0.00714 | R |
| Ddes_1750 |  | FeoA family protein | 1.69 | 0.0429 | P |
| Ddes_1877 |  | Heavy metal transport/detoxification protein | 1.92 | 0.00714 | P |
| Ddes_1951 |  | Flavodoxin | 2.55 | 0.0325 | C |

**Table S6** : Genes differentially expressed in the presence of exogenous NO when grown with nitrate compared to the presence of NO with sulfate as electron acceptor.

| Gene Id | Name | Description | log_2_FC^1^ | padj^2^ | COG |
| --- | --- | --- | --- | --- | --- |
| Ddes_0017 |  | Cobyrinic acid ac-diamide synthase | -1.23 | 0.0281 | D |
| Ddes_0018 |  | response regulator receiver protein | -1.72 | 0.00676 | T |
| Ddes_0021 |  | NLP/P60 protein | 2.94 | 3.13E-06 | M |
| Ddes_0032 |  | tryptophanyl-tRNA synthetase | -1.52 | 0.0129 | J |
| Ddes_0045 |  | glutamate decarboxylase | 2.47 | 0.00381 | E |
| Ddes_0056 |  | tRNA pseudouridine synthase B | 1.88 | 0.0397 | J |
| Ddes_0058 |  | hypothetical protein | -1.66 | 0.00514 | S |
| Ddes_0065 |  | cyclophilin type peptidyl-prolyl cis-trans isomerase | -1.68 | 0.00269 | O |
| Ddes_0070 |  | thiamine-monophosphate kinase | 1.33 | 0.0232 | H |
| Ddes_0073 |  | putative rRNA methylase | 1.34 | 0.0386 | Q |
| Ddes_0076 |  | Dyp-type peroxidase family protein | -1.09 | 0.0489 | P |
| Ddes_0081 | *nrfA* | Nitrite reductase | 2.89 | 0.00707 | P |
| Ddes_0082 | *nrfH* | NapC/NirT cytochrome c family protein | 2.49 | 0.00546 | C |
| Ddes_0087 |  | histone family protein DNA-binding protein | -1.66 | 0.000879 | L |
| Ddes_0117 |  | Thioredoxin domain-containing protein | 1.72 | 0.0209 | C |
| Ddes_0118 |  | glycine/sarcosine/betaine reductase complex protein A | 1.89 | 0.0155 | I |
| Ddes_0122 |  | adenylylsulfate kinase-like protein | 1.98 | 0.000726 | P |
| Ddes_0146 |  | hypothetical protein | -1.44 | 0.00545 | R |
| Ddes_0165 |  | hypothetical protein | -1.25 | 0.0194 | R |
| Ddes_0173 |  | NAD(P)(+) transhydrogenase | 1.75 | 0.0449 | C |
| Ddes_0175 |  | NAD(P)(+) transhydrogenase | 1.75 | 0.00545 | C |
| Ddes_0184 |  | hypothetical protein | 3.05 | 0.00149 |  |
| Ddes_0185 |  | hypothetical protein | 2.43 | 0.000367 |  |
| Ddes_0186 |  | sensor protein ZraS | -1.27 | 0.0312 | T |
| Ddes_0193 |  | flagellar biosynthetic protein FliR | 2.21 | 0.000297 | N |
| Ddes_0199 |  | CutA1 divalent ion tolerance protein | -1.31 | 0.0219 | P |
| Ddes_0203 |  | YbaK/prolyl-tRNA synthetase associated protein | -1.39 | 0.0243 | S |
| Ddes_0204 |  | hypothetical protein | -1.48 | 0.0416 | R |
| Ddes_0210 |  | LysR family transcriptional regulator | -1.22 | 0.0436 | K |
| Ddes_0214 |  | NAD(P)(+) transhydrogenase | 1.64 | 0.0403 | C |
| Ddes_0216 |  | NAD(P)(+) transhydrogenase | 1.73 | 0.00463 | C |
| Ddes_0219 |  | Fis family PAS modulated sigma54 specific transcriptional regulator | -1.82 | 0.0283 | K |
| Ddes_0226 |  | hypothetical protein | -2.27 | 1.61E-05 | S |
| Ddes_0227 |  | hypothetical protein | -1.19 | 0.0271 |  |
| Ddes_0229 |  | hypothetical protein | -1.47 | 0.0149 |  |
| Ddes_0265 |  | hypothetical protein | -2.12 | 0.000197 |  |
| Ddes_0266 |  | hypothetical protein | -1.5 | 0.0451 | K |
| Ddes_0270 |  | DNA mismatch endonuclease Vsr | -1.25 | 0.0267 | L |
| Ddes_0271 |  | DNA-cytosine methyltransferase | -1.21 | 0.025 | L |
| Ddes_0275 |  | transposase IS3/IS911 family protein | -1.44 | 0.0103 | L |
| Ddes_0282 |  | signal peptidase I | -1.19 | 0.033 | U |
| Ddes_0284 |  | Serine-type D-Ala-D-Ala carboxypeptidase | 2.08 | 7.00E-04 | M |
| Ddes_0286 |  | putative AsnC family transcriptional regulator | 1.37 | 0.0216 | K |
| Ddes_0293 |  | TraR/DksA family transcriptional regulator | -1.48 | 0.00274 | T |
| Ddes_0300 |  | methyl-accepting chemotaxis sensory transducer | -1.4 | 0.0191 | N |
| Ddes_0302 |  | hypothetical protein | 1.52 | 0.0266 |  |
| Ddes_0305 |  | hypothetical protein | 2.14 | 0.00514 | O |
| Ddes_0311 |  | hypothetical protein | 2.72 | 2.05E-05 |  |
| Ddes_0312 |  | glycosyl transferase family 9 | 2.16 | 0.000414 | M |
| Ddes_0313 |  | hypothetical protein | 2.21 | 0.00764 |  |
| Ddes_0314 |  | hypothetical protein | 2.23 | 5.84E-05 |  |
| Ddes_0315 |  | phosphoribosylaminoimidazolesuccinocarboxamide synthase | -1.4 | 0.00887 | F |
| Ddes_0317 |  | hypothetical protein | -2.29 | 0.0154 |  |
| Ddes_0323 |  | flagellar basal body-associated protein FliL | -2.32 | 6.24E-05 | N |
| Ddes_0326 |  | Cobyrinic acid ac-diamide synthase | 1.41 | 0.0157 | D |
| Ddes_0327 |  | GTP-binding signal recognition particle SRP54 G- domain-containing protein | 2.54 | 7.06E-05 | N |
| Ddes_0330 |  | XRE family transcriptional regulator | -1.25 | 0.0389 | K |
| Ddes_0331 | *flhA* | flagellar biosynthesis protein FlhA | 2.19 | 0.00144 | N |
| Ddes_0332 | *flhB* | flagellar biosynthesis protein FlhB | 1.75 | 0.0313 | N |
| Ddes_0339 |  | D-cysteine desulfhydrase | -1.8 | 0.0295 | E |
| Ddes_0340 |  | Na+/H+ antiporter NhaC | -2.1 | 0.00147 | C |
| Ddes_0341 |  | GntR family transcriptional regulator | -1.32 | 0.0411 | K |
| Ddes_0350 |  | phosphoglycerate mutase | -1.6 | 0.00713 | G |
| Ddes_0353 |  | hypothetical protein | 2.26 | 0.000208 |  |
| Ddes_0361 | *hisI* | phosphoribosyl-AMP cyclohydrolase | -1.23 | 0.0388 | E |
| Ddes_0373 |  | flagellar basal body-associated protein FliL | 1.18 | 0.0339 | N |
| Ddes_0376 |  | flagellar biosynthesis protein FliO | 1.98 | 0.000232 | N |
| Ddes_0377 | *fliP* | flagellar biosynthesis protein FliP | 2.1 | 0.00339 | N |
| Ddes_0378 |  | flagellar biosynthetic protein FliQ | 2.18 | 0.000589 | N |
| Ddes_0382 |  | carbon-monoxide dehydrogenase, catalytic subunit | -2.51 | 0.000726 | C |
| Ddes_0389 |  | putative chemotaxis phosphatase, CheZ | -1.56 | 0.0113 | N |
| Ddes_0391 |  | hypothetical protein | 2.99 | 0.000422 | N |
| Ddes_0392 | *flgG* | flagellar basal body rod protein FlgG | 2.65 | 4.28E-05 | N |
| Ddes_0393 |  | flagella basal body P-ring formation protein FlgA | 2.65 | 7.06E-05 | N |
| Ddes_0394 | *flgH* | flagellar basal body L-ring protein | 2.52 | 5.29E-05 | N |
| Ddes_0430 |  | AraC family transcriptional regulator | -2.24 | 0.000259 | K |
| Ddes_0431 |  | transposase IS3/IS911 family protein | -1.52 | 0.00545 | L |
| Ddes_0435 |  | Cobyrinic acid ac-diamide synthase | -1.85 | 0.0225 | D |
| Ddes_0445 |  | ribosomal RNA large subunit methyltransferase N | -1.27 | 0.0252 | R |
| Ddes_0446 |  | metal dependent phosphohydrolase | -2.1 | 0.00605 | J |
| Ddes_0456 |  | hypothetical protein | 1.33 | 0.0312 |  |
| Ddes_0489 | *infC* | translation initiation factor IF-3 | -1.44 | 0.00707 | J |
| Ddes_0493 |  | RNP-1 like RNA-binding protein | -1.72 | 0.0434 | R |
| Ddes_0496 |  | hypothetical protein | -1.47 | 0.0107 |  |
| Ddes_0500 | *moaC* | Molybdenum cofactor biosynthesis protein C | 1.27 | 0.0407 | H |
| Ddes_0524 |  | HPP family protein | 1.82 | 0.000879 | T |
| Ddes_0525 |  | 4Fe-4S ferredoxin iron-sulfur binding domain-containing protein | 1.6 | 0.0162 | C |
| Ddes_0526 |  | pyridoxamine 5'-phosphate oxidase-like protein | 2.11 | 0.00803 | R |
| Ddes_0527 |  | flavodoxin family protein | 1.49 | 0.0241 | C |
| Ddes_0529 |  | flagellar protein FliS | 1.14 | 0.0436 | N |
| Ddes_0556 |  | formate dehydrogenase subunit beta | 2.4 | 0.0165 | C |
| Ddes_0558 |  | cytochrome c class III | 1.52 | 0.0148 |  |
| Ddes_0559 |  | Phenylacetate--CoA ligase | -1.21 | 0.0257 | H |
| Ddes_0560 |  | iojap-like protein | -1.39 | 0.035 | S |
| Ddes_0593 |  | hypothetical protein | -1.41 | 0.00714 |  |
| Ddes_0594 |  | sodium/calcium exchanger membrane protein | -1.58 | 0.00397 | P |
| Ddes_0596 |  | hypothetical protein | 1.72 | 0.00243 |  |
| Ddes_0602 |  | peroxiredoxin | -1.81 | 0.0286 | O |
| Ddes_0609 |  | N-acetylmuramoyl-L-alanine amidase | 2.21 | 0.00516 | M |
| Ddes_0612 |  | ABC transporter-like protein | -1.39 | 0.03 | V |
| Ddes_0614 | *napC* | NapC/NirT cytochrome c domain-containing protein | 2.28 | 0.00124 | C |
| Ddes_0615 | *napM* | hypothetical protein | 2.66 | 1.02E-05 | O |
| Ddes_0616 | *napA* | Periplasmic nitrate reductase | 2.35 | 0.00453 | C |
| Ddes_0617 | *napD* | hypothetical protein | 1.99 | 0.00254 |  |
| Ddes_0625 |  | hypothetical protein | 1.82 | 0.00228 | L |
| Ddes_0641 |  | Alanine--glyoxylate transaminase | 2.61 | 0.00632 | E |
| Ddes_0642 |  | amino acid permease-associated protein | -1.27 | 0.0367 | E |
| Ddes_0644 |  | FeoA family protein | -3.45 | 8.82E-06 | P |
| Ddes_0645 |  | FeoA family protein | -3.83 | 9.00E-06 | P |
| Ddes_0646 |  | small GTP-binding protein | -1.85 | 0.0228 | P |
| Ddes_0647 |  | hypothetical protein | -2.33 | 6.39E-05 |  |
| Ddes_0648 |  | hypothetical protein | -2.14 | 0.00181 | P |
| Ddes_0657 |  | hypothetical protein | -1.39 | 0.0185 | R |
| Ddes_0691 | *clpP* | ATP-dependent Clp protease proteolytic subunit | -1.07 | 0.0431 | O |
| Ddes_0693 |  | hypothetical protein | -1.71 | 0.00707 |  |
| Ddes_0695 |  | hypothetical protein | 2.98 | 3.02E-06 |  |
| Ddes_0696 | *truA* | tRNA pseudouridine synthase A | 1.42 | 0.033 | J |
| Ddes_0697 |  | hypothetical protein | 2.08 | 0.000457 | S |
| Ddes_0698 |  | flagellar export protein FliJ | 2.31 | 0.00044 | N |
| Ddes_0702 |  | hypothetical protein: part of a putative gene transfer island | -1.54 | 0.0449 |  |
| Ddes_0705 |  | hypothetical protein: part of a putative gene transfer island | -2.8 | 1.61E-05 |  |
| Ddes_0706 |  | hypothetical protein: part of a putative gene transfer island | -3.59 | 2.59E-06 |  |
| Ddes_0707 |  | hypothetical protein: part of a putative gene transfer island | -2.51 | 0.000194 |  |
| Ddes_0708 |  | GCN5-like N-acetyltransferase: part of a putative gene transfer island | -2.59 | 1.17E-05 | K |
| Ddes_0709 |  | hypothetical protein: part of a putative gene transfer island | -2.86 | 0.000297 |  |
| Ddes_0710 |  | hypothetical protein: part of a putative gene transfer island | -2.72 | 3.20E-05 |  |
| Ddes_0711 |  | hypothetical protein: part of a putative gene transfer island | -2.86 | 1.05E-05 | U |
| Ddes_0712 |  | hypothetical protein | -2.8 | 3.02E-06 |  |
| Ddes_0713 |  | hypothetical protein | -3.75 | 1.02E-05 |  |
| Ddes_0714 |  | hypothetical protein | -3.66 | 1.45E-05 |  |
| Ddes_0715 |  | hypothetical protein | -3.79 | 1.47E-07 |  |
| Ddes_0716 |  | hypothetical protein | -4.14 | 1.78E-08 |  |
| Ddes_0717 |  | hypothetical protein | -3.87 | 4.50E-06 |  |
| Ddes_0718 |  | hypothetical protein | -3.76 | 1.56E-07 |  |
| Ddes_0719 |  | hypothetical protein | -3.32 | 3.41E-06 |  |
| Ddes_0720 |  | hypothetical protein | -3.25 | 1.72E-06 |  |
| Ddes_0721 |  | hypothetical protein | -3.64 | 4.84E-08 |  |
| Ddes_0722 |  | hypothetical protein | -3.66 | 1.78E-08 |  |
| Ddes_0723 |  | hypothetical protein | -3.89 | 1.16E-07 | R |
| Ddes_0725 |  | hypothetical protein | -2.39 | 0.000234 |  |
| Ddes_0726 |  | putative phage repressor | -1.95 | 0.00514 | K |
| Ddes_0759 |  | hypothetical protein | 3.05 | 0.000493 | R |
| Ddes_0763 |  | hypothetical protein | 2.45 | 0.00395 |  |
| Ddes_0767 |  | hypothetical protein | 2.59 | 0.00714 | S |
| Ddes_0770 |  | diacylglycerol kinase | -1.4 | 0.0354 | M |
| Ddes_0771 |  | hypothetical protein | -1.55 | 0.0123 |  |
| Ddes_0772 |  | hypothetical protein | -1.75 | 0.0139 | E |
| Ddes_0784 |  | ABC transporter-like protein | 1.45 | 0.0244 | P |
| Ddes_0787 |  | glycine cleavage system H protein | 2.23 | 0.00658 | E |
| Ddes_0794 |  | hypothetical protein | 1.92 | 0.00559 | S |
| Ddes_0806 |  | L-aspartate oxidase | 1.86 | 0.0371 | H |
| Ddes_0807 |  | quinolinate synthetase | 1.73 | 0.0383 | H |
| Ddes_0819 |  | putative phage repressor | -1.95 | 0.0122 | K |
| Ddes_0822 |  | glycine betaine ABC transporter substrate-binding protein | 2.39 | 9.22E-05 | E |
| Ddes_0823 |  | binding-protein-dependent transport systems inner membrane component | 1.28 | 0.0417 | E |
| Ddes_0838 |  | hypothetical protein | 1.49 | 0.0382 | F |
| Ddes_0843 |  | BadM/Rrf2 family transcriptional regulator | 2.87 | 0.00152 | K |
| Ddes_0844 |  | response regulator receiver protein | 2.92 | 8.00E-04 | T |
| Ddes_0846 |  | hmc operon protein 5 | -1.87 | 0.000319 | C |
| Ddes_0847 |  | hypothetical protein | -2.61 | 8.29E-07 |  |
| Ddes_0851 |  | glucose-6-phosphate isomerase | 1.42 | 0.0298 | G |
| Ddes_0865 |  | hypothetical protein | 3.11 | 4.10E-06 |  |
| Ddes_0869 |  | Membrane-bound lytic murein transglycosylase B-like protein | 1.35 | 0.0295 | M |
| Ddes_0870 |  | hypothetical protein | 3.56 | 0.00656 |  |
| Ddes_0875 |  | polysaccharide biosynthesis domain-containing protein | -1.3 | 0.0345 | M |
| Ddes_0877 |  | glucose-1-phosphate cytidylyltransferase | -1.62 | 0.00956 | J |
| Ddes_0883 |  | hypothetical protein | 2.99 | 0.00409 | C |
| Ddes_0884 |  | hypothetical protein | 2.71 | 1.02E-05 |  |
| Ddes_0885 |  | hypothetical protein | 1.64 | 0.0199 |  |
| Ddes_0897 |  | Rubrerythrin | -2.8 | 0.000349 | C |
| Ddes_0898 |  | hypothetical protein | -1.23 | 0.0295 | S |
| Ddes_0913 |  | hypothetical protein | 1.21 | 0.0285 | S |
| Ddes_0917 | *rpsT* | 30S ribosomal protein S20 | -1.07 | 0.038 | J |
| Ddes_0925 |  | Cse3 family CRISPR-associated protein | -1.29 | 0.0268 |  |
| Ddes_0930 |  | hypothetical protein | 1.96 | 0.0122 | S |
| Ddes_0931 |  | hypothetical protein | 1.71 | 0.0119 | C |
| Ddes_0932 |  | hypothetical protein | 2.07 | 0.00932 | T |
| Ddes_0937 |  | hypothetical protein | -2.15 | 0.0179 | L |
| Ddes_0943 |  | transposase IS3/IS911 family protein | -1.36 | 0.0149 | L |
| Ddes_0957 |  | tRNA(Ile)-lysidine synthetase | 1.52 | 0.046 | D |
| Ddes_0966 |  | DNA protecting protein DprA | 1.29 | 0.0281 | L |
| Ddes_0981 |  | hypothetical protein | 1.72 | 0.0129 |  |
| Ddes_0989 |  | ABC transporter-like protein | -1.33 | 0.0431 | H |
| Ddes_0996 | *upp* | Uracil phosphoribosyltransferase | -1.48 | 0.00658 | F |
| Ddes_0999 |  | putative lipoprotein | -1.5 | 0.0129 |  |
| Ddes_1000 |  | Multidrug resistance efflux pump-like protein | 2.63 | 1.02E-05 | V |
| Ddes_1012 | *hslU* | ATP-dependent protease ATPase subunit HslU | -1.78 | 0.00158 | O |
| Ddes_1013 |  | group 1 glycosyl transferase | 1.54 | 0.0113 | M |
| Ddes_1028 |  | flagellin domain-containing protein | 3.32 | 0.000208 | N |
| Ddes_1044 | *greA* | Transcription elongation factor greA | -1.37 | 0.00849 | K |
| Ddes_1074 |  | hemerythrin-like metal-binding protein | 2.35 | 0.00899 | P |
| Ddes_1076 |  | Fe-S cluster assembly protein NifU | 2.5 | 0.00463 | C |
| Ddes_1077 |  | BadM/Rrf2 family transcriptional regulator | 2.12 | 0.00269 | K |
| Ddes_1079 |  | 4Fe-4S ferredoxin iron-sulfur binding domain-containing protein | 1.71 | 0.0482 | C |
| Ddes_1080 |  | HAD-superfamily hydrolase | 1.74 | 0.0371 | R |
| Ddes_1096 |  | carbamoyl-phosphate synthase, large subunit | -1.2 | 0.0344 | E |
| Ddes_1104 |  | MraZ protein | -1.93 | 0.000493 | S |
| Ddes_1108 |  | UDP-N-acetylmuramyl-tripeptide synthetase | 1.83 | 0.00251 | M |
| Ddes_1111 | *murD* | UDP-N-acetylmuramoylalanine--D-glutamate ligase | 1.27 | 0.0299 | M |
| Ddes_1113 | *murG* | undecaprenyldiphospho-muramoylpentapeptide beta-N- acetylglucosaminyltransferase | 1.18 | 0.0442 | M |
| Ddes_1114 | *murC* | UDP-N-acetylmuramate--L-alanine ligase | 1.32 | 0.0206 | M |
| Ddes_1118 |  | cell division protein FtsZ | 1.5 | 0.0216 | D |
| Ddes_1146 |  | Fis family transcriptional regulator | -1.49 | 0.0189 | K |
| Ddes_1161 |  | AraC family transcriptional regulator | -2.58 | 1.02E-05 | F |
| Ddes_1176 |  | Triose-phosphate isomerase | 1.4 | 0.0253 | G |
| Ddes_1227 |  | phage transcriptional regulator AlpA | -1.42 | 0.0403 | K |
| Ddes_1228 |  | hypothetical protein | -1.84 | 8.00E-04 |  |
| Ddes_1232 |  | metal dependent phosphohydrolase | -1.57 | 0.0194 |  |
| Ddes_1239 |  | NQR2 and RnfD family protein | 1.6 | 0.00748 | C |
| Ddes_1240 |  | FMN-binding domain-containing protein | 1.59 | 0.0063 | C |
| Ddes_1241 |  | electron transport complex RsxE subunit | 1.31 | 0.0206 | C |
| Ddes_1242 |  | RnfABCDGE type electron transport complex subunit A | 2.2 | 5.84E-05 | C |
| Ddes_1243 |  | ferredoxin | 2 | 0.000346 | C |
| Ddes_1244 |  | ApbE family lipoprotein | 1.6 | 0.00463 | H |
| Ddes_1247 |  | hypothetical protein | -4.37 | 6.26E-06 |  |
| Ddes_1259 |  | flagellar hook-length control protein | 2.57 | 4.88E-05 | N |
| Ddes_1260 |  | flagellar hook capping protein | 2.55 | 7.19E-06 | N |
| Ddes_1261 |  | hypothetical protein | 2.31 | 0.00254 | N |
| Ddes_1263 |  | transposase IS3/IS911 family protein | -1.55 | 0.00425 | L |
| Ddes_1264 |  | ArsR family transcriptional regulator | 3.79 | 0.000729 | K |
| Ddes_1265 |  | heavy metal translocating P-type ATPase | 2.72 | 0.000419 | P |
| Ddes_1270 |  | hypothetical protein | -1.7 | 0.000701 |  |
| Ddes_1297 |  | hypothetical protein | -1.86 | 0.0153 |  |
| Ddes_1315 |  | hypothetical protein | 1.46 | 0.0203 | R |
| Ddes_1316 |  | PpkA-like protein | 1.45 | 0.0228 |  |
| Ddes_1340 |  | ABC-2 type transporter | -1.91 | 0.000194 | V |
| Ddes_1341 |  | ABC transporter-like protein | -1.89 | 0.000531 | V |
| Ddes_1342 |  | HlyD family secretion protein | -2.05 | 5.66E-05 | M |
| Ddes_1343 |  | TonB-dependent receptor | -2.91 | 6.24E-05 | P |
| Ddes_1344 |  | Biopolymer transport protein ExbD/TolR | -3.49 | 9.42E-09 | U |
| Ddes_1345 |  | isochorismate synthase | -2.78 | 2.59E-06 | H |
| Ddes_1346 |  | chorismate mutase-like protein | -2.61 | 2.64E-05 | E |
| Ddes_1355 |  | microcompartments protein | 2.19 | 0.00277 | C |
| Ddes_1356 |  | aldehyde-alcohol dehydrogenase 2 | 2.31 | 0.00164 | C |
| Ddes_1357 |  | Formate C-acetyltransferase | 2.38 | 0.00514 | C |
| Ddes_1358 |  | glycyl-radical enzyme activating protein family | 1.76 | 0.0286 | O |
| Ddes_1359 |  | microcompartments protein | 2.07 | 0.0134 | C |
| Ddes_1360 |  | acetaldehyde dehydrogenase | 2.52 | 0.000484 | C |
| Ddes_1361 |  | microcompartments protein | 2.62 | 0.000468 | C |
| Ddes_1362 |  | Propanediol utilization protein | 2.13 | 0.00157 | Q |
| Ddes_1363 |  | ethanolamine utilization protein EutJ family protein | 2.19 | 0.00102 | E |
| Ddes_1364 |  | hypothetical protein | 1.65 | 0.0174 |  |
| Ddes_1365 |  | Ethanolamine utilization protein EutN/carboxysome structural protein Ccml | 1.94 | 0.00605 | C |
| Ddes_1366 |  | microcompartments protein | 2.4 | 0.000234 | C |
| Ddes_1367 |  | hypothetical protein | 1.8 | 0.00192 |  |
| Ddes_1368 |  | microcompartments protein | 1.94 | 0.00119 | C |
| Ddes_1369 |  | iron-containing alcohol dehydrogenase | 1.24 | 0.0383 | C |
| Ddes_1370 |  | Respiratory-chain NADH dehydrogenase domain 51 kDa subunit | 1.51 | 0.00847 | C |
| Ddes_1371 |  | microcompartments protein | 1.79 | 0.00332 | C |
| Ddes_1386 | *rd2* | Rubredoxin-2 | 2.46 | 0.0099 | C |
| Ddes_1388 |  | Fur family ferric uptake regulator | -1.77 | 0.0167 | P |
| Ddes_1396 |  | group 1 glycosyl transferase | -1.53 | 0.00453 | M |
| Ddes_1411 |  | cyclic nucleotide-binding protein | -1.37 | 0.0166 | T |
| Ddes_1430 |  | ATP-dependent Clp protease adaptor protein ClpS | -1.65 | 0.00193 | S |
| Ddes_1431 |  | adenylate cyclase | -1.38 | 0.00838 | F |
| Ddes_1452 | *rplY* | 50S ribosomal protein L25 | -1.4 | 0.00967 | J |
| Ddes_1458 |  | Deoxyribonuclease I | -1.82 | 0.00218 | L |
| Ddes_1459 |  | MotA/TolQ/ExbB proton channel | -2.06 | 0.00268 | U |
| Ddes_1460 |  | Biopolymer transport protein ExbD/TolR | -1.61 | 0.00166 | U |
| Ddes_1463 |  | hypothetical protein | 1.61 | 0.0124 | Q |
| Ddes_1484 |  | hypothetical protein | -1.71 | 8.00E-04 |  |
| Ddes_1494 |  | Catalase | -3.06 | 0.000211 | P |
| Ddes_1505 | *thiH* | thiamine biosynthesis protein ThiH | -1.73 | 0.0365 | H |
| Ddes_1507 |  | sodium:dicarboxylate symporter | 2.15 | 0.042 | C |
| Ddes_1528 |  | hydro-lyase, Fe-S type, tartrate/fumarate subfamily subunit alpha | 2.72 | 0.000468 | C |
| Ddes_1529 |  | hydro-lyase, Fe-S type, tartrate/fumarate subfamily subunit beta | 2.16 | 0.000194 | C |
| Ddes_1530 |  | fumarate reductase respiratory complex transmembrane subunit | 2.54 | 5.66E-05 | C |
| Ddes_1531 |  | fumarate reductase flavoprotein subunit | 2.64 | 0.000367 | C |
| Ddes_1533 |  | CBS domain containing membrane protein | 2.09 | 0.0395 | R |
| Ddes_1534 |  | malate dehydrogenase | 1.79 | 0.0397 | C |
| Ddes_1548 | *groL* | 60 kDa chaperonin | -1.88 | 0.0203 | O |
| Ddes_1559 |  | hypothetical protein | 2.76 | 1.61E-05 |  |
| Ddes_1560 |  | family 3 extracellular solute-binding protein | 1.35 | 0.0446 | E |
| Ddes_1562 |  | polar amino acid ABC transporter inner membrane subunit | -1.38 | 0.0383 | E |
| Ddes_1563 |  | ABC transporter-like protein | -1.13 | 0.0491 | E |
| Ddes_1569 |  | ATPase FliI/YscN | 1.23 | 0.0361 | N |
| Ddes_1570 |  | hypothetical protein | 1.72 | 0.00843 | N |
| Ddes_1571 |  | flagellar motor switch protein FliG | 1.14 | 0.0453 | N |
| Ddes_1573 |  | flagellar M-ring protein FliF | 2.54 | 0.00012 | N |
| Ddes_1574 |  | flagellar hook-basal body complex subunit FliE | 2.62 | 3.20E-05 | N |
| Ddes_1575 |  | flagellar basal-body rod protein FlgC | 2.94 | 1.05E-05 | N |
| Ddes_1576 | *flgB* | flagellar basal body rod protein FlgB | 3.35 | 4.57E-05 | N |
| Ddes_1579 |  | phosphoadenosine phosphosulfate reductase | -1.53 | 0.0234 | R |
| Ddes_1581 |  | hypothetical protein | -1.92 | 0.00546 |  |
| Ddes_1585 |  | Fe^++^ transport protein similar to FeoB | -2.55 | 3.25E-05 | P |
| Ddes_1586 |  | hypothetical protein | -3.72 | 6.79E-05 |  |
| Ddes_1587 | *trpA* | Tryptophan synthase alpha chain | 3.81 | 3.41E-06 | E |
| Ddes_1588 |  | tryptophan synthase subunit beta | 3.74 | 3.02E-06 | E |
| Ddes_1589 | *trpF* | N-(5'-phosphoribosyl)anthranilate isomerase | 1.94 | 0.0021 | E |
| Ddes_1590 |  | Indole-3-glycerol-phosphate synthase | 2.44 | 3.20E-05 | E |
| Ddes_1591 |  | anthranilate phosphoribosyltransferase | 2.84 | 1.02E-05 | E |
| Ddes_1607 |  | hypothetical protein | -1.44 | 0.0113 | O |
| Ddes_1624 |  | DNA polymerase III delta | -1.91 | 0.0113 | L |
| Ddes_1632 | *rplK* | 50S ribosomal protein L11 | -1.15 | 0.038 | J |
| Ddes_1647 |  | beta-lactamase domain-containing protein | 1.89 | 0.0218 | R |
| Ddes_1661 |  | NADH dehydrogenase (quinone) | -3.12 | 0.000229 | C |
| Ddes_1668 |  | 4Fe-4S ferredoxin iron-sulfur binding domain-containing protein | 2.93 | 0.000701 | C |
| Ddes_1669 |  | NADH-ubiquinone oxidoreductase chain 49kDa | 1.41 | 0.0204 | C |
| Ddes_1671 |  | NADH ubiquinone oxidoreductase 20 kDa subunit | 2.49 | 0.0021 | C |
| Ddes_1672 |  | respiratory-chain NADH dehydrogenase subunit 1 | 1.98 | 0.00207 | C |
| Ddes_1674 |  | putative diguanylate cyclase | -1.22 | 0.0365 | N |
| Ddes_1678 |  | K+-transporting ATPase subunit B | 1.61 | 0.00714 | P |
| Ddes_1688 |  | BFD domain-containing protein (2Fe-2S)-binding domain-containing protein | 1.9 | 0.0413 | R |
| Ddes_1702 |  | hypothetical protein | -1.92 | 0.000397 |  |
| Ddes_1705 |  | thiamine pyrophosphate TPP binding domain-containing protein | -1.86 | 0.0133 | E |
| Ddes_1706 |  | auxin efflux carrier | -1.28 | 0.0222 | R |
| Ddes_1715 |  | hypothetical protein | -1.88 | 7.00E-04 | S |
| Ddes_1718 |  | hypothetical protein | -1.48 | 0.0113 |  |
| Ddes_1719 |  | GntR family transcriptional regulator | -2.15 | 0.00984 | K |
| Ddes_1729 |  | ATPase | -1.86 | 0.01 | R |
| Ddes_1730 |  | diguanylate cyclase and serine/threonine protein kinase with TPR repeats | 2.22 | 0.00506 | R |
| Ddes_1734 |  | hypothetical protein | -1.44 | 0.0482 | P |
| Ddes_1735 |  | adenylyltransferase | -1.22 | 0.0286 | R |
| Ddes_1737 |  | thioesterase superfamily protein | -1.61 | 0.0019 | Q |
| Ddes_1745 |  | hypothetical protein | 1.38 | 0.0211 | S |
| Ddes_1750 |  | FeoA family protein | -3.04 | 3.24E-05 | P |
| Ddes_1753 |  | hypothetical protein | -1.17 | 0.0457 |  |
| Ddes_1757 |  | Undecaprenyl-phosphate galactose phosphotransferase, WbaP | -1.26 | 0.0281 | M |
| Ddes_1763 |  | threonine dehydratase, biosynthetic | -1.19 | 0.0232 | E |
| Ddes_1782 |  | major facilitator superfamily protein | -2.17 | 7.31E-05 | E |
| Ddes_1791 |  | Ribonuclease H | 1.93 | 0.0456 | L |
| Ddes_1807 |  | twin-arginine translocation protein, TatB subunit | 2.49 | 0.00554 | U |
| Ddes_1810 |  | putative lipoprotein | 1.67 | 0.00568 |  |
| Ddes_1811 | *hisA* | 1-(5-phosphoribosyl)-5-[(5-phosphoribosylamino)methylideneamino] imidazole-4-carboxamide isomerase | 1.18 | 0.0456 | E |
| Ddes_1813 |  | small multidrug resistance protein | -1.22 | 0.0496 | P |
| Ddes_1817 |  | coenzyme F390 synthetase | 1.87 | 0.0299 | H |
| Ddes_1819 |  | hypothetical protein | 1.59 | 0.0425 |  |
| Ddes_1833 |  | ribonucleotide-diphosphate reductase subunit alpha | -1.49 | 0.00863 | F |
| Ddes_1839 |  | phosphoribosylaminoimidazole synthetase | -1.21 | 0.033 | F |
| Ddes_1843 |  | hypothetical protein | 1.24 | 0.0403 |  |
| Ddes_1845 | *murA* | UDP-N-acetylglucosamine 1-carboxyvinyltransferase | 1.54 | 0.01 | M |
| Ddes_1846 |  | FAD-dependent pyridine nucleotide-disulfide oxidoreductase | 3.44 | 0.000484 | R |
| Ddes_1847 |  | hypothetical protein | 3.94 | 3.88E-05 | S |
| Ddes_1848 |  | hypothetical protein | -1.43 | 0.0216 |  |
| Ddes_1852 |  | hypothetical protein | 1.48 | 0.0195 |  |
| Ddes_1864 |  | Dinitrogenase iron-molybdenum cofactor biosynthesis protein | -4.49 | 3.11E-09 | S |
| Ddes_1865 |  | Cobyrinic acid ac-diamide synthase | -3.95 | 2.46E-07 | C |
| Ddes_1866 |  | 4Fe-4S ferredoxin iron-sulfur binding domain-containing protein | -3.2 | 0.00127 | C |
| Ddes_1867 |  | Dinitrogenase iron-molybdenum cofactor biosynthesis protein | -3.45 | 0.00101 | S |
| Ddes_1876 |  | heavy metal translocating P-type ATPase | -1.81 | 0.0155 | P |
| Ddes_1897 |  | SNO glutamine amidotransferase | -1.48 | 0.00658 | H |
| Ddes_1898 | *pdxS* | Pyridoxal biosynthesis lyase pdxS | -1.58 | 0.0155 | H |
| Ddes_1899 |  | hypothetical protein | 1.39 | 0.0139 |  |
| Ddes_1902 |  | hypothetical protein | 2.25 | 0.00707 | S |
| Ddes_1905 | *ilvC* | Ketol-acid reductoisomerase | 1.36 | 0.0399 | E |
| Ddes_1909 |  | hypothetical protein | -1.45 | 0.0281 | S |
| Ddes_1914 |  | hypothetical protein | -1.49 | 0.00324 | J |
| Ddes_1921 |  | hypothetical protein | -1.7 | 0.0364 |  |
| Ddes_1922 |  | ABC transporter-like protein | -1.29 | 0.0446 | O |
| Ddes_1925 |  | putative ArsR family transcriptional regulator | -2.44 | 0.00143 | K |
| Ddes_1926 |  | type 11 methyltransferase | -2.05 | 0.0185 | Q |
| Ddes_1930 |  | preprotein translocase subunit YajC | -1.08 | 0.0456 | U |
| Ddes_1935 |  | hypothetical protein | -1.34 | 0.0218 |  |
| Ddes_1939 |  | putative diguanylate cyclase | 2.71 | 0.00189 | T |
| Ddes_1946 | *thiH* | thiamine biosynthesis protein ThiH | -1.71 | 0.0289 | H |
| Ddes_1951 |  | Flavodoxin | -1.87 | 0.0147 | C |
| Ddes_1960 |  | phosphate transporter | -1.22 | 0.0329 | P |
| Ddes_1961 |  | hypothetical protein | -1.52 | 0.0114 | P |
| Ddes_1973 | *aroK* | shikimate kinase | -1.37 | 0.00834 | E |
| Ddes_1974 |  | hypothetical protein | -2.28 | 0.000369 |  |
| Ddes_1975 |  | hypothetical protein | -1.34 | 0.0125 | C |
| Ddes_1981 |  | aldehyde dehydrogenase | -2.66 | 0.000701 | C |
| Ddes_2000 |  | flagellar hook-associated protein 3 | 1.77 | 0.00425 | N |
| Ddes_2001 |  | flagellar hook-associated protein FlgK | 2.41 | 2.00E-04 | N |
| Ddes_2002 |  | FlgN family protein | 3.58 | 0.000156 |  |
| Ddes_2003 |  | flagellar protein FlgJ | 2.86 | 2.91E-05 | M |
| Ddes_2004 | *flgI* | Flagellar P-ring protein | 2.93 | 1.81E-05 | N |
| Ddes_2006 |  | hypothetical protein | -1.6 | 0.0165 |  |
| Ddes_2016 |  | hypothetical protein | -1.74 | 0.0221 |  |
| Ddes_2030 |  | Spermine synthase | -1.31 | 0.0349 | R |
| Ddes_2039 |  | 4Fe-4S ferredoxin iron-sulfur binding domain-containing protein | 1.87 | 0.0101 | C |
| Ddes_2041 |  | hypothetical protein | 1.33 | 0.0388 |  |
| Ddes_2044 |  | hypothetical protein | -1.31 | 0.0467 |  |
| Ddes_2050 |  | MoeA domain-containing protein domain I and II | 1.65 | 0.0394 | H |
| Ddes_2051 |  | iron-containing alcohol dehydrogenase | 3.47 | 0.000838 | C |
| Ddes_2052 |  | TOBE domain-containing protein | -1.64 | 0.0417 | R |
| Ddes_2057 |  | hypothetical protein | 2.1 | 0.0281 |  |
| Ddes_2058 |  | group 1 glycosyl transferase | 1.48 | 0.0482 | M |
| Ddes_2066 |  | hypothetical protein | 1.27 | 0.0403 |  |
| Ddes_2075 | *lipB* | Octanoyltransferase | -2.13 | 0.000455 | H |
| Ddes_2079 |  | lytic transglycosylase | 2.29 | 2.91e-05 | M |
| Ddes_2091 |  | hypothetical protein | 1.58 | 0.0221 | N |
| Ddes_2098 |  | heterodisulfide reductase subunit C | 1.62 | 0.00774 | C |
| Ddes_2103 |  | oxidoreductase FAD/NAD(P)-binding domain-containing protein | 1.36 | 0.0266 | C |
| Ddes_2106 |  | ABC transporter-like protein | 2.16 | 0.00641 | V |
| Ddes_2133 |  | hypothetical protein | -1.49 | 0.0266 |  |
| Ddes_2153 |  | glutamyl-tRNA(Gln) amidotransferase subunit C | -1.22 | 0.0281 | J |
| Ddes_2156 |  | sodium:dicarboxylate symporter | -1.42 | 0.0122 | C |
| Ddes_2157 |  | hypothetical protein | -1.59 | 0.00218 |  |
| Ddes_2158 |  | small multidrug resistance protein | -2.05 | 3.20E-05 | P |
| Ddes_2159 |  | peptidase M23 | -1.43 | 0.0091 | M |
| Ddes_2164 |  | hypothetical protein | -2.15 | 0.00122 |  |
| Ddes_2165 |  | thiamine biosynthesis protein ThiS | 2.23 | 0.00065 | H |
| Ddes_2166 | *thiG* | thiazole synthase | 1.74 | 0.00906 | H |
| Ddes_2202 |  | NAD-dependent epimerase/dehydratase | 2 | 0.000229 | G |
| Ddes_2205 |  | oxygen-independent coproporphyrinogen III oxidase | 2.3 | 8.57E-05 | H |
| Ddes_2219 |  | family 2 glycosyl transferase | -1.43 | 0.00599 | M |
| Ddes_2223 |  | hypothetical protein | -2.52 | 2.77E-06 |  |
| Ddes_2224 |  | K potassium transporter | -1.19 | 0.045 | P |
| Ddes_2228 |  | transposase IS3/IS911 family protein | -2.27 | 8.00E-04 | L |
| Ddes_2233 |  | XRE family transcriptional regulator | -3.44 | 3.88E-05 | K |
| Ddes_2236 |  | integrase family protein | -1.21 | 0.0373 | L |
| Ddes_2245 |  | class I and II aminotransferase | -1.08 | 0.0398 | E |
| Ddes_2253 |  | hypothetical protein | -1.22 | 0.0278 |  |
| Ddes_2256 |  | hypothetical protein | -1.71 | 0.0401 |  |
| Ddes_2261 |  | diguanylate cyclase | -1.85 | 0.00228 | T |
| Ddes_2265 |  | hypothetical protein | -1.11 | 0.0496 |  |
| Ddes_2267 |  | DNA polymerase III subunit delta' | -1.61 | 0.042 | L |
| Ddes_2282 |  | flagellin domain-containing protein | 2.24 | 0.0257 | N |
| Ddes_2285 |  | hypothetical protein | -1.84 | 0.000879 |  |
| Ddes_2287 |  | S-adenosylmethionine synthetase | -2 | 0.0114 | H |
| Ddes_2290 | *ribB* | 3,4-dihydroxy-2-butanone 4-phosphate synthase | -1.87 | 0.000972 | H |
| Ddes_2292 |  | AMMECR1 domain-containing protein | -1.14 | 0.038 | S |
| Ddes_2296 |  | hypothetical protein | -1.49 | 0.00605 |  |
| Ddes_2302 |  | hemolysin A | -1.21 | 0.0334 | J |
| Ddes_2308 |  | Antibiotic biosynthesis monooxygenase | 1.53 | 0.0097 | S |
| Ddes_2334 |  | anaerobic cobalt chelatase | 2.28 | 0.0122 | H |
| Ddes_2339 |  | Asparaginase | 1.16 | 0.04 | E |
| Ddes_2341 |  | major facilitator superfamily protein | -1.23 | 0.0286 | E |
| Ddes_2355 |  | transposase IS3/IS911 family protein | -1.43 | 0.00797 | L |
| Ddes_2356 |  | hypothetical protein | -1.2 | 0.0332 | K |
| Ddes_2359 |  | integrase family protein | -1.67 | 0.0322 | L |
| Ddes_2367 |  | heme exporter protein CcmA | 1.92 | 0.0148 | V |
| Ddes_2379 |  | hypothetical protein | 1.16 | 0.05 | S |
| Ddes_R0005 |  | tRNA-Ser | -1.93 | 0.00466 |  |
| Ddes_R0009 |  | tRNA-Ala | 2.9 | 0.0142 |  |
| Ddes_R0016 |  | tRNA-Leu | -1.53 | 0.0456 |  |
| Ddes_R0032 | *ssrA* |  | 2.67 | 0.0283 |  |
| Ddes_R0033 |  | tRNA-Arg | -3.34 | 5.87E-05 |  |
| Ddes_R0039 |  | tRNA-Ala | 2.97 | 0.00863 |  |
| Ddes_R0049 |  | tRNA-Thr | -1.38 | 0.00631 |  |
| Ddes_R0050 |  | tRNA-Tyr | -1.48 | 0.00546 |  |
| Ddes_R0051 |  | tRNA-Gly | -1.53 | 0.00218 |  |
| Ddes_R0060 |  | 6S RNA | 2.5 | 0.0244 |  |
| EBG00001164127 |  |  | -1.52 | 0.00302 |  |
| EBG00001164140 |  |  | -1.21 | 0.0207 |  |
| EBG00001164145 |  |  | 2.5 | 0.0246 |  |
| EBG00001164148 |  |  | -1.77 | 0.00757 |  |
| EBG00001164154 |  |  | -1.82 | 0.00847 |  |
| EBG00001164166 |  |  | 1.63 | 0.0162 |  |
| EBG00001164170 |  |  | -1.27 | 0.015 |  |
| EBG00001164172 |  |  | -3.85 | 4.50E-06 |  |
| EBG00001164173 |  |  | -1.33 | 0.0241 |  |
| EBG00001164177 |  |  | -2.08 | 0.043 |  |
| EBG00001164186 |  |  | -3.9 | 0.0154 |  |
| EBG00001164187 |  |  | 2.68 | 0.0281 |  |
| EBG00001164193 |  |  | -1.84 | 0.00339 |  |

^1^. FC : fold change.

^2^. Pdaj : adjusted p-value

Supplementary Table S7.

| **Gene ID** | **Name** | **description** | **Hcpr1 binding sites predicted with f1-PSSM pval1e-4** | **edgeR sulfate+NO vs sulfate edgeR.DEG** | **edgeR sulfate+NO vs sulfate edgeR.log2FC** | **edgeR nitrate vs sulfate edgeR.DEG** | **edgeR nitrate vs sulfate edgeR.log2FC** | **Detailed description** | **Cross-references** |
| --- | --- | --- | --- | --- | --- | --- | --- | --- | --- |
| Ddes_0528 | hcpR1 | Crp/Fnr family transcriptional regulator | 2 | 1 | 3.05 | 1 | 1.85 | PFAM: cyclic nucleotide-binding; SMART: regulatory protein Crp; KEGG: glo:Glov_2803 transcriptional regulator, Crp/Fnr family; Crp/Fnr family transcriptional regulator | YP_002479117.1;Ddes_0528;Ddes_0528;7284195;220903805;YP_002479117.1;YP_002479117 |
| Ddes_1427 | Ddes_1427 | hypothetical protein | 2 | 1 | -1.5 | 1 | -2.38 | KEGG: dvl:Dvul_1530 hypothetical protein; hypothetical protein | YP_002480007.1;Ddes_1427;Ddes_1427;7285123;220904695;YP_002480007.1;YP_002480007 |
| Ddes_1642 | Ddes_1642 | hypothetical protein | 1 | 1 | -1.85 | 1 | -3.29 | KEGG: dvl:Dvul_2817 hypothetical protein; hypothetical protein | YP_002480219.1;Ddes_1642;Ddes_1642;7285345;220904907;YP_002480219.1;YP_002480219 |
| Ddes_1643 | Ddes_1643 | Sigma 54 interacting domain-containing protein | 1 | 1 | -1.45 | 1 | -1.52 | PFAM: sigma-54 factor interaction domain-containing protein; histidine kinase HAMP region domain protein; ATPase associated with various cellular activities AAA_5; SMART: AAA ATPase; KEGG: dvu:DVU0151 HAMP domain/sigma-54 interaction domain-containing protein; sigma 54 interacting domain-containing protein | YP_002480220.1;Ddes_1643;Ddes_1643;7285346;220904908;YP_002480220.1;YP_002480220 |
| Ddes_1824 | Ddes_1824 | molybdopterin binding domain-containing protein | 2 | 1 | -2.14 | 1 | -1.55 | PFAM: molybdopterin binding domain; KEGG: dvl:Dvul_1573 molybdopterin binding domain-containing protein; molybdopterin binding domain-containing protein | YP_002480399.1;Ddes_1824;Ddes_1824;7285537;220905087;YP_002480399.1;YP_002480399 |
| Ddes_0087 | Ddes_0087 | histone family protein DNA-binding protein | 2 | 0 | 0.224 | 1 | -1.46 | PFAM: histone family protein DNA-binding protein; KEGG: dvl:Dvul_2207 histone family protein DNA-binding protein; histone family protein DNA-binding protein | YP_002478685.1;Ddes_0087;Ddes_0087;7283739;220903373;YP_002478685.1;YP_002478685 |
| Ddes_0154 | Ddes_0154 | Na+/solute symporter | 2 | 0 | -1.16 | 1 | -1.2 | PFAM: Na+/solute symporter; KEGG: drm:Dred_2834 Na+/solute symporter; Na+/solute symporter | YP_002478752.1;Ddes_0154;Ddes_0154;7283808;220903440;YP_002478752.1;YP_002478752 |
| Ddes_0214 | Ddes_0214 | NAD(P)(+) transhydrogenase | 2 | 0 | 0.311 | 1 | 2.33 | PFAM: alanine dehydrogenase/PNT domain protein; KEGG: pgn:PGN_1120 putative NADPH-NAD transhydrogenase; NAD(P)(+) transhydrogenase | YP_002478807.1;Ddes_0214;Ddes_0214;7283868;220903495;YP_002478807.1;YP_002478807 |
| Ddes_0419 | Ddes_0419 | 4-vinyl reductase 4VR | 1 | 0 | -0.804 | 1 | -1.75 | PFAM: 4-vinyl reductase 4VR; KEGG: mbn:Mboo_0934 4-vinyl reductase, 4VR; 4-vinyl reductase 4VR | YP_002479009.1;Ddes_0419;Ddes_0419;7284083;220903697;YP_002479009.1;YP_002479009 |
| Ddes_0446 | Ddes_0446 | metal dependent phosphohydrolase | 2 | 0 | -0.726 | 1 | -3.19 | PFAM: metal-dependent phosphohydrolase HD sub domain; KEGG: dvl:Dvul_0462 metal dependent phosphohydrolase; metal dependent phosphohydrolase | YP_002479036.1;Ddes_0446;Ddes_0446;7284111;220903724;YP_002479036.1;YP_002479036 |
| Ddes_0614 | Ddes_0614 | NapC/NirT cytochrome c domain-containing protein | 4 | 0 | 0.744 | 1 | 3.67 | KEGG: hsm:HSM_1276 NapC/NirT cytochrome c domain-containing protein; NapC/NirT cytochrome c domain-containing protein | YP_002479201.1;Ddes_0614;Ddes_0614;7284286;220903889;YP_002479201.1;YP_002479201 |
| Ddes_0843 | Ddes_0843 | BadM/Rrf2 family transcriptional regulator | 2 | 0 | 1.24 | 1 | 3.81 | TIGRFAM: transcriptional regulator, Rrf2 family; PFAM: protein of unknown function UPF0074; KEGG: dvl:Dvul_1204 BadM/Rrf2 family transcriptional regulator; BadM/Rrf2 family transcriptional regulator | YP_002479428.1;Ddes_0843;Ddes_0843;7284518;220904116;YP_002479428.1;YP_002479428 |
| Ddes_0844 | Ddes_0844 | response regulator receiver protein | 2 | 0 | 0.943 | 1 | 3.55 | KEGG: dvl:Dvul_1201 response regulator receiver protein; response regulator receiver protein | YP_002479429.1;Ddes_0844;Ddes_0844;7284519;220904117;YP_002479429.1;YP_002479429 |
| Ddes_1153 | Ddes_1153 | transglutaminase family protein cysteine peptidase BTLCP | 2 | 0 | -0.279 | 1 | -1.06 | PFAM: transglutaminase family protein cysteine peptidase BTLCP; KEGG: dvl:Dvul_1975 hypothetical protein; transglutaminase | YP_002479735.1;Ddes_1153;Ddes_1153;7284835;220904423;YP_002479735.1;YP_002479735 |
| Ddes_1164 | Ddes_1164 | Cupin 2 barrel domain-containing protein | 2 | 0 | 0.772 | 1 | -2.61 | PFAM: Cupin 2 conserved barrel domain protein; KEGG: dps:DP0971 hypothetical protein; cupin | YP_002479746.1;Ddes_1164;Ddes_1164;7284846;220904434;YP_002479746.1;YP_002479746 |
| Ddes_1411 | Ddes_1411 | cyclic nucleotide-binding protein | 2 | 0 | -1.11 | 1 | -1.52 | PFAM: cyclic nucleotide-binding; KEGG: dsy:DSY3652 hypothetical protein; cyclic nucleotide-binding protein | YP_002479991.1;Ddes_1411;Ddes_1411;7285106;220904679;YP_002479991.1;YP_002479991 |
| Ddes_1526 | Ddes_1526 | hypothetical protein | 2 | 0 | -0.743 | 1 | -1.85 | hypothetical protein | YP_002480106.1;Ddes_1526;Ddes_1526;7285224;220904794;YP_002480106.1;YP_002480106 |
| Ddes_1527 | Ddes_1527 | anion transporter | 2 | 0 | 0.657 | 1 | 2.16 | TIGRFAM: anion transporter; PFAM: sodium/sulphate symporter; Citrate transporter; TRAP C4-dicarboxylate transport system permease DctM subunit; KEGG: mmq:MmarC5_1523 citrate transporter; anion transporter | YP_002480107.1;Ddes_1527;Ddes_1527;7285225;220904795;YP_002480107.1;YP_002480107 |
| Ddes_1538 | Ddes_1538 | family 5 extracellular solute-binding protein | 2 | 0 | -0.455 | 1 | -1.7 | PFAM: extracellular solute-binding protein family 5; KEGG: dvl:Dvul_1145 extracellular solute-binding protein; family 5 extracellular solute-binding protein | YP_002480118.1;Ddes_1538;Ddes_1538;7285236;220904806;YP_002480118.1;YP_002480118 |
| Ddes_1606 | Ddes_1606 | Sel1 domain-containing protein | 1 | 0 | -0.246 | 1 | -1.66 | PFAM: Sel1 domain protein repeat-containing protein; KEGG: hiq:CGSHiGG_00130 Sel1 domain-containing protein; Sel1 domain-containing protein | YP_002480183.1;Ddes_1606;Ddes_1606;7285305;220904871;YP_002480183.1;YP_002480183 |
| Ddes_1607 | Ddes_1607 | hypothetical protein | 1 | 0 | -0.00634 | 1 | -1.85 | KEGG: hypothetical protein; hypothetical protein | YP_002480184.1;Ddes_1607;Ddes_1607;7285306;220904872;YP_002480184.1;YP_002480184 |
| Ddes_1673 | Ddes_1673 | NADH dehydrogenase (quinone) | 2 | 0 | 0.451 | 1 | 2.1 | PFAM: NADH/Ubiquinone/plastoquinone (complex I); KEGG: dvl:Dvul_2501 NADH dehydrogenase (quinone); NADH dehydrogenase (quinone) | YP_002480250.1;Ddes_1673;Ddes_1673;7285376;220904938;YP_002480250.1;YP_002480250 |
| Ddes_1674 | Ddes_1674 | putative diguanylate cyclase | 2 | 0 | -0.662 | 1 | -1.59 | KEGG: cdf:CD2965 putative signaling protein; putative diguanylate cyclase | YP_002480251.1;Ddes_1674;Ddes_1674;7285377;220904939;YP_002480251.1;YP_002480251 |
| Ddes_1704 | Ddes_1704 | hypothetical protein | 2 | 0 | -0.0341 | 1 | -2.1 | KEGG: dvl:Dvul_2198 hypothetical protein; hypothetical protein | YP_002480280.1;Ddes_1704;Ddes_1704;7285415;220904968;YP_002480280.1;YP_002480280 |
| Ddes_1705 | Ddes_1705 | thiamine pyrophosphate TPP binding domain-containing protein | 2 | 0 | 0.0105 | 1 | -2.4 | PFAM: thiamine pyrophosphate protein domain protein TPP-binding; thiamine pyrophosphate protein central region; thiamine pyrophosphate protein TPP binding domain protein; KEGG: pnu:Pnuc_1088 thiamine pyrophosphate binding domain-containing protein; thiamine pyrophosphate domain-containing TPP-binding protein | YP_002480281.1;Ddes_1705;Ddes_1705;7285416;220904969;YP_002480281.1;YP_002480281 |
| Ddes_1831 | Ddes_1831 | cyclopropane fatty acyl phospholipid synthase | 2 | 0 | -0.0653 | 1 | -1.88 | catalyzes the transfer of a methylene group from S-adenosyl-L-methionine to the cis double bond of an unsaturated fatty acid chain resulting in the replacement of the double bond with a methylene bridge; cyclopropane-fatty-acyl-phospholipid synthase | YP_002480406.1;Ddes_1831;Ddes_1831;7285544;220905094;YP_002480406.1;YP_002480406 |
| Ddes_1905 | Ddes_1905 | Ketol-acid reductoisomerase | 2 | 0 | 0.197 | 1 | 1.55 | catalyzes the formation of (R)-2,3-dihydroxy-3-methylbutanoate from (S)-2-hydroxy-2-methyl-3-oxobutanoate in valine and isoleucine biosynthesis; ketol-acid reductoisomerase | YP_002480479.1;Ddes_1905;Ddes_1905;7285620;220905167;YP_002480479.1;YP_002480479 |
| Ddes_1943 | Ddes_1943 | iron-containing alcohol dehydrogenase | 2 | 0 | 0.295 | 1 | 1.97 | PFAM: iron-containing alcohol dehydrogenase; KEGG: dvl:Dvul_0825 iron-containing alcohol dehydrogenase; iron-containing alcohol dehydrogenase | YP_002480517.1;Ddes_1943;Ddes_1943;7285659;220905205;YP_002480517.1;YP_002480517 |
| Ddes_1951 | Ddes_1951 | Flavodoxin | 2 | 0 | 0.233 | 1 | -4.19 | TIGRFAM: flavodoxin; PFAM: flavodoxin/nitric oxide synthase; KEGG: dvu:DVU2680 flavodoxin; flavodoxin | YP_002480525.1;Ddes_1951;Ddes_1951;7285667;220905213;YP_002480525.1;YP_002480525 |
| Ddes_2051 | Ddes_2051 | iron-containing alcohol dehydrogenase | 2 | 0 | -0.212 | 1 | 2.97 | PFAM: iron-containing alcohol dehydrogenase; KEGG: cbh:CLC_1475 alcohol dehydrogenase, iron-containing; iron-containing alcohol dehydrogenase | YP_002480625.1;Ddes_2051;Ddes_2051;7285767;220905313;YP_002480625.1;YP_002480625 |
| Ddes_2098 | Ddes_2098 | heterodisulfide reductase subunit C | 2 | 0 | 0.329 | 1 | 1.76 | KEGG: dvl:Dvul_0826 heterodisulfide reductase, C subunit; heterodisulfide reductase subunit C | YP_002480672.1;Ddes_2098;Ddes_2098;7285814;220905360;YP_002480672.1;YP_002480672 |
| Ddes_2228 | Ddes_2228 | transposase IS3/IS911 family protein | 3 | 0 | 0.247 | 1 | -3.22 | PFAM: transposase IS3/IS911 family protein; KEGG: dvu:DVU2011 ISD1, transposase OrfA; transposase IS3/IS911 family protein | YP_002480802.1;Ddes_2228;Ddes_2228;7285944;220905490;YP_002480802.1;YP_002480802 |
| Ddes_2252 | Ddes_2252 | hypothetical protein | 2 | 0 | -1.13 | 1 | -2.74 | KEGG: dvl:Dvul_2064 ATP synthase protein I; hypothetical protein | YP_002480825.1;Ddes_2252;Ddes_2252;7285969;220905513;YP_002480825.1;YP_002480825 |
| Ddes_2269 | Ddes_2269 | hypothetical protein | 2 | 0 | 0.495 | 1 | 1.75 | KEGG: dvu:DVU0741 hypothetical protein; hypothetical protein | YP_002480842.1;Ddes_2269;Ddes_2269;7285986;220905530;YP_002480842.1;YP_002480842 |
| Ddes_2296 | Ddes_2296 | hypothetical protein | 2 | 0 | 0.12 | 1 | -1.59 | KEGG: dvl:Dvul_2533 hypothetical protein; hypothetical protein | YP_002480869.1;Ddes_2296;Ddes_2296;7286014;220905557;YP_002480869.1;YP_002480869 |
| Ddes_2338 | Ddes_2338 | Phenylacetate--CoA ligase | 4 | 0 | 0.442 | 1 | 1.48 | KEGG: dvl:Dvul_2452 phenylacetate--CoA ligase; phenylacetate--CoA ligase | YP_002480910.1;Ddes_2338;Ddes_2338;7286056;220905598;YP_002480910.1;YP_002480910 |
| Ddes_2345 | Ddes_2345 | Fis family sigma-54 specific transcriptional regulator | 2 | 0 | -0.959 | 1 | -1.04 | PFAM: sigma-54 factor interaction domain-containing protein; helix-turn-helix Fis-type; ATPase associated with various cellular activities AAA_5; PAS fold-3 domain protein; SMART: PAC repeat-containing protein; AAA ATPase; KEGG: sfu:Sfum_1160 sigma54 specific transcriptional regulator, fis family; Fis family sigma-54 specific transcriptional regulator | YP_002480917.1;Ddes_2345;Ddes_2345;7286063;220905605;YP_002480917.1;YP_002480917 |
| Ddes_2355 | Ddes_2355 | transposase IS3/IS911 family protein | 2 | 0 | 0.0804 | 1 | -2.29 | PFAM: transposase IS3/IS911 family protein; KEGG: dvu:DVU2011 ISD1, transposase OrfA; transposase IS3/IS911 family protein | YP_002480927.1;Ddes_2355;Ddes_2355;7286074;220905615;YP_002480927.1;YP_002480927 |
| Ddes_2151 | dnaK | Chaperone protein DnaK | 2 | 0 | 0.0483 | 1 | -1.72 | heat shock protein 70; assists in folding of nascent polypeptide chains; refolding of misfolded proteins; utilizes ATPase activity to help fold; co-chaperones are DnaJ and GrpE; multiple copies in some bacteria; molecular chaperone DnaK | YP_002480725.1;dnaK;dnaK;Ddes_2151;7285867;220905413;YP_002480725.1;YP_002480725 |
| Ddes_2150 | Ddes_2150 | Split-Soret cytochrome c | 2 | 1 | -1.74 | 0 | -1.16 | KEGG: dol:Dole_3017 split soret cytochrome c precursor; split soret cytochrome c | YP_002480724.1;Ddes_2150;Ddes_2150;7285866;220905412;YP_002480724.1;YP_002480724 |
| Ddes_2068 | argJ | bifunctional ornithine acetyltransferase/N-acetylglutamate synthase protein | 1 | 0 | -0.124 | 0 | 0.109 | bifunctional arginine biosynthesis protein ArgJ; functions at the 1st and 5th steps in arginine biosynthesis; involved in synthesis of acetylglutamate from glutamate and acetyl-CoA and ornithine by transacetylation between acetylornithine and glutmate; bifunctional ornithine acetyltransferase/N-acetylglutamate synthase | YP_002480642.1;argJ;argJ;Ddes_2068;7285784;220905330;YP_002480642.1;YP_002480642 |
| Ddes_0119 | Ddes_0119 | methyl-accepting chemotaxis sensory transducer | 2 | 0 | -0.909 | 0 | -0.635 | PFAM: chemotaxis sensory transducer; KEGG: dvl:Dvul_0942 methyl-accepting chemotaxis sensory transducer; methyl-accepting chemotaxis sensory transducer | YP_002478717.1;Ddes_0119;Ddes_0119;7283771;220903405;YP_002478717.1;YP_002478717 |
| Ddes_0171 | Ddes_0171 | Alanine racemase | 2 | 0 | -1.07 | 0 | 0.176 | KEGG: mta:Moth_2167 alanine racemase; TIGRFAM: alanine racemase; PFAM: alanine racemase domain protein; alanine racemase | YP_002478769.1;Ddes_0171;Ddes_0171;7283825;220903457;YP_002478769.1;YP_002478769 |
| Ddes_0181 | Ddes_0181 | histone family protein DNA-binding protein | 2 | 0 | -0.678 | 0 | -0.548 | PFAM: histone family protein DNA-binding protein; KEGG: dvl:Dvul_2538 histone family protein DNA-binding protein; histone family protein DNA-binding protein | YP_002478777.1;Ddes_0181;Ddes_0181;7283835;220903465;YP_002478777.1;YP_002478777 |
| Ddes_0182 | Ddes_0182 | Radical SAM domain-containing protein | 2 | 0 | -0.345 | 0 | -0.89 | PFAM: Radical SAM domain protein; SMART: Elongator protein 3/MiaB/NifB; KEGG: lip:LI0429 coproporphyrinogen III oxidase-like protein; radical SAM protein | YP_002478778.1;Ddes_0182;Ddes_0182;7283836;220903466;YP_002478778.1;YP_002478778 |
| Ddes_0269 | Ddes_0269 | putative plasmid conjugal transfer protein | 2 | 0 | -0.98 | 0 | -0.0551 | KEGG: xfn:XfasM23_2250 putative plasmid conjugal transfer protein; putative plasmid conjugal transfer protein | YP_002478862.1;Ddes_0269;Ddes_0269;7283925;220903550;YP_002478862.1;YP_002478862 |
| Ddes_0285 | Ddes_0285 | Cache sensor-containing methyl-accepting chemotaxis sensory transducer | 1 | 0 | -0.463 | 0 | 0.651 | PFAM: histidine kinase HAMP region domain protein; Cache domain protein; chemotaxis sensory transducer; KEGG: dvl:Dvul_1305 methyl-accepting chemotaxis sensory transducer; Cache sensor-containing methyl-accepting chemotaxis sensory transducer | YP_002478878.1;Ddes_0285;Ddes_0285;7283941;220903566;YP_002478878.1;YP_002478878 |
| Ddes_0454 | Ddes_0454 | Sulfate adenylyltransferase | 2 | 0 | -0.912 | 0 | -1.18 | KEGG: dvl:Dvul_1771 sulfate adenylyltransferase; TIGRFAM: sulfate adenylyltransferase; PFAM: ATP-sulfurylase; sulfate adenylyltransferase | YP_002479044.1;Ddes_0454;Ddes_0454;7284119;220903732;YP_002479044.1;YP_002479044 |
| Ddes_0460 | Ddes_0460 | Pyruvate carboxylase | 1 | 0 | -0.5 | 0 | -0.553 | PFAM: Carbamoyl-phosphate synthase L chain ATP-binding; Carbamoyl-phosphate synthetase large chain domain protein; KEGG: dvl:Dvul_1015 pyruvate carboxylase; pyruvate carboxylase | YP_002479050.1;Ddes_0460;Ddes_0460;7284125;220903738;YP_002479050.1;YP_002479050 |
| Ddes_0461 | Ddes_0461 | purine nucleoside phosphorylase | 1 | 0 | -0.155 | 0 | -0.134 | catalyzes the formation of a purine and ribose phosphate from a purine nucleoside; in E. coli this enzyme functions in xanthosine degradation; purine nucleoside phosphorylase | YP_002479051.1;Ddes_0461;Ddes_0461;7284126;220903739;YP_002479051.1;YP_002479051 |
| Ddes_0472 | Ddes_0472 | Extracellular ligand-binding receptor | 2 | 0 | 0.0962 | 0 | -0.356 | PFAM: Extracellular ligand-binding receptor; KEGG: dvl:Dvul_2254 extracellular ligand-binding receptor; extracellular ligand-binding receptor | YP_002479062.1;Ddes_0472;Ddes_0472;7284137;220903750;YP_002479062.1;YP_002479062 |
| Ddes_0555 | Ddes_0555 | formate dehydrogenase subunit alpha | 2 | 0 | -0.326 | 0 | 1.04 | KEGG: dvu:DVU2812 formate dehydrogenase, alpha subunit, selenocysteine-containing; Contains selenocysteine; TIGRFAM: formate dehydrogenase, alpha subunit; PFAM: molybdopterin oxidoreductase; molydopterin dinucleotide-binding region; molybdopterin oxidoreductase Fe4S4 region; formate dehydrogenase subunit alpha | YP_002479143.1;Ddes_0555;Ddes_0555;7284223;220903831;YP_002479143.1;YP_002479143 |
| Ddes_0657 | Ddes_0657 | hypothetical protein | 1 | 0 | 0.203 | 0 | -0.824 | PFAM: TPR repeat-containing protein; response regulator receiver; Tetratricopeptide TPR_2 repeat protein; SMART: Tetratricopeptide domain protein; KEGG: dvl:Dvul_0431 response regulator receiver; hypothetical protein | YP_002479244.1;Ddes_0657;Ddes_0657;7284330;220903932;YP_002479244.1;YP_002479244 |
| Ddes_0658 | Ddes_0658 | hypothetical protein | 1 | 0 | 0.569 | 0 | 0.178 | hypothetical protein | YP_002479245.1;Ddes_0658;Ddes_0658;7284331;220903933;YP_002479245.1;YP_002479245 |
| Ddes_0808 | Ddes_0808 | nicotinate-nucleotide pyrophosphorylase | 1 | 0 | 0.0746 | 0 | -0.0738 | KEGG: dvl:Dvul_1353 nicotinate-nucleotide pyrophosphorylase; TIGRFAM: nicotinate-nucleotide pyrophosphorylase; PFAM: Quinolinate phosphoribosyl transferase; nicotinate-nucleotide pyrophosphorylase | YP_002479394.1;Ddes_0808;Ddes_0808;7284483;220904082;YP_002479394.1;YP_002479394 |
| Ddes_0809 | Ddes_0809 | magnesium transporter | 1 | 0 | 0.571 | 0 | 0.826 | TIGRFAM: magnesium transporter; PFAM: CBS domain containing protein; MgtE integral membrane region; MgtE intracellular region; KEGG: dvl:Dvul_1354 magnesium transporter; magnesium transporter | YP_002479395.1;Ddes_0809;Ddes_0809;7284484;220904083;YP_002479395.1;YP_002479395 |
| Ddes_0842 | Ddes_0842 | BadM/Rrf2 family transcriptional regulator | 2 | 0 | -0.0891 | 0 | -0.556 | TIGRFAM: transcriptional regulator, Rrf2 family; PFAM: protein of unknown function UPF0074; KEGG: dvl:Dvul_1205 BadM/Rrf2 family transcriptional regulator; BadM/Rrf2 family transcriptional regulator | YP_002479427.1;Ddes_0842;Ddes_0842;7284517;220904115;YP_002479427.1;YP_002479427 |
| Ddes_0857 | Ddes_0857 | peptidoglycan-associated lipoprotein | 1 | 0 | -0.309 | 0 | -0.412 | TIGRFAM: peptidoglycan-associated lipoprotein; PFAM: OmpA/MotB domain protein; KEGG: lip:LI0691 outer membrane protein and related peptidoglycan-associated (LipO)proteins; peptidoglycan-associated lipoprotein | YP_002479442.1;Ddes_0857;Ddes_0857;7284533;220904130;YP_002479442.1;YP_002479442 |
| Ddes_0889 | Ddes_0889 | 4Fe-4S ferredoxin iron-sulfur binding domain-containing protein | 2 | 0 | -0.136 | 0 | -0.168 | PFAM: 4Fe-4S ferredoxin iron-sulfur binding domain protein; KEGG: dvl:Dvul_0113 4Fe-4S ferredoxin iron-sulfur binding domain-containing protein; 4Fe-4S ferredoxin | YP_002479474.1;Ddes_0889;Ddes_0889;7284565;220904162;YP_002479474.1;YP_002479474 |
| Ddes_0988 | Ddes_0988 | sodium/hydrogen exchanger | 2 | 0 | 0.0871 | 0 | 0.0405 | PFAM: TrkA-N domain protein; TrkA-C domain protein; sodium/hydrogen exchanger; KEGG: dvu:DVU2302 glutathione-regulated potassium-efflux system protein KefB, putative; sodium/hydrogen exchanger | YP_002479573.1;Ddes_0988;Ddes_0988;7284668;220904261;YP_002479573.1;YP_002479573 |
| Ddes_1032 | Ddes_1032 | nickel ABC transporter periplasmic nickel-binding protein | 1 | 0 | -0.616 | 0 | 0.792 | KEGG: rru:Rru_A2277 nickel ABC transporter, periplasmic nickel-binding; TIGRFAM: nickel ABC transporter, periplasmic nickel-binding protein; PFAM: extracellular solute-binding protein family 5; nickel ABC transporter substrate-binding protein | YP_002479617.1;Ddes_1032;Ddes_1032;7284713;220904305;YP_002479617.1;YP_002479617 |
| Ddes_1078 | Ddes_1078 | Homoserine O-succinyltransferase | 1 | 0 | 1.42 | 0 | 1.08 | PFAM: homoserine O-succinyltransferase; KEGG: afl:Aflv_1464 homoserine O-succinyltransferase; Homoserine O-succinyltransferase | YP_002479662.1;Ddes_1078;Ddes_1078;7284760;220904350;YP_002479662.1;YP_002479662 |
| Ddes_1080 | Ddes_1080 | HAD-superfamily hydrolase | 2 | 0 | -0.618 | 0 | -0.973 | TIGRFAM: HAD-superfamily hydrolase, subfamily IA, variant 3; HAD-superfamily hydrolase, subfamily IA, variant 1; PFAM: Haloacid dehalogenase domain protein hydrolase; KEGG: hmo:HM1_1613 phosphoglycolate phosphatase, putative; HAD-superfamily hydrolase | YP_002479664.1;Ddes_1080;Ddes_1080;7284762;220904352;YP_002479664.1;YP_002479664 |
| Ddes_1081 | Ddes_1081 | aspartate/ornithine carbamoyltransferase Asp/Orn-binding region | 2 | 0 | -0.84 | 0 | -0.439 | PFAM: aspartate/ornithine carbamoyltransferase Asp/Orn-binding region; KEGG: dvl:Dvul_1899 ornithine carbamoyltransferase; aspartate/ornithine carbamoyltransferase Asp/Orn-binding region | YP_002479665.1;Ddes_1081;Ddes_1081;7284763;220904353;YP_002479665.1;YP_002479665 |
| Ddes_1173 | Ddes_1173 | Inositol-phosphate phosphatase | 2 | 0 | 0.0876 | 0 | -0.167 | PFAM: inositol monophosphatase; KEGG: dvu:DVU1680 inositol-1-monophosphatase; inositol-phosphate phosphatase | YP_002479755.1;Ddes_1173;Ddes_1173;7284855;220904443;YP_002479755.1;YP_002479755 |
| Ddes_1188 | Ddes_1188 | CoA-substrate-specific enzyme activase | 2 | 0 | -0.403 | 0 | -0.998 | TIGRFAM: CoA-substrate-specific enzyme activase; PFAM: ATPase BadF/BadG/BcrA/BcrD type; CoA protein activase; KEGG: cth:Cthe_1329 putative CoA-substrate-specific enzyme activase; CoA-substrate-specific enzyme activase | YP_002479770.1;Ddes_1188;Ddes_1188;7284870;220904458;YP_002479770.1;YP_002479770 |
| Ddes_1212 | Ddes_1212 | hypothetical protein | 2 | 0 | -0.0454 | 0 | -1.03 | KEGG: dvl:Dvul_0781 hypothetical protein; hypothetical protein | YP_002479794.1;Ddes_1212;Ddes_1212;7284894;220904482;YP_002479794.1;YP_002479794 |
| Ddes_1237 | Ddes_1237 | cytochrome c family protein | 2 | 0 | 0.572 | 0 | 1.33 | KEGG: dvl:Dvul_0523 cytochrome c family protein; cytochrome c family protein | YP_002479818.1;Ddes_1237;Ddes_1237;7284920;220904506;YP_002479818.1;YP_002479818 |
| Ddes_1349 | Ddes_1349 | Citrate transporter | 2 | 0 | 0.0916 | 0 | -0.078 | PFAM: Citrate transporter; TrkA-C domain protein; TRAP C4-dicarboxylate transport system permease DctM subunit; KEGG: ppw:PputW619_3221 citrate transporter; citrate transporter | YP_002479929.1;Ddes_1349;Ddes_1349;7285043;220904617;YP_002479929.1;YP_002479929 |
| Ddes_1412 | Ddes_1412 | hypothetical protein | 2 | 0 | -0.409 | 0 | 0.261 | KEGG: drm:Dred_0607 hypothetical protein; hypothetical protein | YP_002479992.1;Ddes_1412;Ddes_1412;7285107;220904680;YP_002479992.1;YP_002479992 |
| Ddes_1426 | Ddes_1426 | excinuclease ABC subunit B | 2 | 0 | -0.225 | 0 | 0.195 | The UvrABC repair system catalyzes the recognition and processing of DNA lesions. The beta-hairpin of the Uvr-B subunit is inserted between the strands, where it probes for the presence of a lesion; excinuclease ABC subunit B | YP_002480006.1;Ddes_1426;Ddes_1426;7285122;220904694;YP_002480006.1;YP_002480006 |
| Ddes_1436 | Ddes_1436 | Formate C-acetyltransferase | 2 | 0 | -0.552 | 0 | 0.263 | PFAM: formate C-acetyltransferase glycine radical; pyruvate formate-lyase PFL; KEGG: dvl:Dvul_0491 formate C-acetyltransferase; Formate C-acetyltransferase | YP_002480016.1;Ddes_1436;Ddes_1436;7285132;220904704;YP_002480016.1;YP_002480016 |
| Ddes_1437 | Ddes_1437 | amidohydrolase 2 | 4 | 0 | -1.08 | 0 | 0.18 | PFAM: amidohydrolase 2; KEGG: dvu:DVU2820 amidohydrolase family protein; amidohydrolase 2 | YP_002480017.1;Ddes_1437;Ddes_1437;7285133;220904705;YP_002480017.1;YP_002480017 |
| Ddes_1517 | Ddes_1517 | UspA domain-containing protein | 3 | 0 | 0.357 | 0 | -0.178 | PFAM: UspA domain protein; KEGG: dvu:DVU0423 universal stress protein; UspA domain-containing protein | YP_002480097.1;Ddes_1517;Ddes_1517;7285215;220904785;YP_002480097.1;YP_002480097 |
| Ddes_1539 | Ddes_1539 | Non-specific serine/threonine protein kinase | 2 | 0 | -1.1 | 0 | -0.895 | KEGG: dvl:Dvul_1144 SNF2-related protein; PFAM: SNF2-related protein; helicase domain protein; zinc finger SWIM domain protein; SMART: DEAD-like helicases; Non-specific serine/threonine protein kinase | YP_002480119.1;Ddes_1539;Ddes_1539;7285237;220904807;YP_002480119.1;YP_002480119 |
| Ddes_1546 | Ddes_1546 | D-lactate dehydrogenase (cytochrome) | 2 | 0 | 0.517 | 0 | 0.907 | PFAM: 4Fe-4S ferredoxin iron-sulfur binding domain protein; FAD linked oxidase domain protein; KEGG: dvl:Dvul_2725 D-lactate dehydrogenase (cytochrome); D-lactate dehydrogenase | YP_002480126.1;Ddes_1546;Ddes_1546;7285244;220904814;YP_002480126.1;YP_002480126 |
| Ddes_1568 | Ddes_1568 | pyruvate phosphate dikinase | 2 | 0 | -0.72 | 0 | 0.0999 | PFAM: pyruvate phosphate dikinase PEP/pyruvate-binding; PEP-utilising protein mobile region; KEGG: dvl:Dvul_0151 pyruvate, water dikinase; pyruvate phosphate dikinase | YP_002480147.1;Ddes_1568;Ddes_1568;7285266;220904835;YP_002480147.1;YP_002480147 |
| Ddes_1649 | Ddes_1649 | NADH:flavin oxidoreductase/NADH oxidase | 1 | 0 | 0.139 | 0 | 1.28 | PFAM: NADH:flavin oxidoreductase/NADH oxidase; KEGG: rru:Rru_A0144 NADH:flavin oxidoreductase/NADH oxidase; NADH:flavin oxidoreductase | YP_002480226.1;Ddes_1649;Ddes_1649;7285352;220904914;YP_002480226.1;YP_002480226 |
| Ddes_1748 | Ddes_1748 | acetate/CoA ligase | 4 | 0 | -0.381 | 0 | 0.982 | TIGRFAM: acetate/CoA ligase; PFAM: AMP-dependent synthetase and ligase; KEGG: dvl:Dvul_0401 acetate--CoA ligase; acetate/CoA ligase | YP_002480324.1;Ddes_1748;Ddes_1748;7285460;220905012;YP_002480324.1;YP_002480324 |
| Ddes_1778 | Ddes_1778 | ABC transporter periplasmic substrate-binding protein | 2 | 0 | -0.735 | 0 | -0.412 | KEGG: dvu:DVU0745 ABC transporter, periplasmic substrate-binding protein; ABC transporter periplasmic substrate-binding protein | YP_002480353.1;Ddes_1778;Ddes_1778;7285491;220905041;YP_002480353.1;YP_002480353 |
| Ddes_1779 | Ddes_1779 | hypothetical protein | 2 | 0 | -1.33 | 0 | 0.115 | hypothetical protein | YP_002480354.1;Ddes_1779;Ddes_1779;7285492;220905042;YP_002480354.1;YP_002480354 |
| Ddes_1825 | Ddes_1825 | putative lipoprotein | 2 | 0 | 0.323 | 0 | -0.0499 | KEGG: dvl:Dvul_0245 putative lipoprotein; putative lipoprotein | YP_002480400.1;Ddes_1825;Ddes_1825;7285538;220905088;YP_002480400.1;YP_002480400 |
| Ddes_1880 | Ddes_1880 | NADH dehydrogenase (quinone) | 2 | 0 | -0.738 | 0 | -0.0741 | PFAM: NADH/Ubiquinone/plastoquinone (complex I); KEGG: dvl:Dvul_0969 NADH dehydrogenase (quinone); NADH dehydrogenase (quinone) | YP_002480454.1;Ddes_1880;Ddes_1880;7285595;220905142;YP_002480454.1;YP_002480454 |
| Ddes_1933 | Ddes_1933 | diaminopimelate aminotransferase | 2 | 0 | -0.05 | 0 | -0.934 | catalyzes the transamination of diaminopimelate with 2-oxoglutarate to produce tetrahydrodipicolinate and glutamate; diaminopimelate aminotransferase | YP_002480507.1;Ddes_1933;Ddes_1933;7285649;220905195;YP_002480507.1;YP_002480507 |
| Ddes_1947 | Ddes_1947 | basic membrane lipoprotein | 2 | 0 | -0.103 | 0 | -0.363 | PFAM: basic membrane lipoprotein; KEGG: tte:TTE0457 surface lipoprotein; basic membrane lipoprotein | YP_002480521.1;Ddes_1947;Ddes_1947;7285663;220905209;YP_002480521.1;YP_002480521 |
| Ddes_2043 | Ddes_2043 | diaminopropionate ammonia-lyase | 1 | 0 | -0.784 | 0 | -0.781 | catalyzes the formation of pyruvate from 2,3-diaminopropionate; diaminopropionate ammonia-lyase | YP_002480617.1;Ddes_2043;Ddes_2043;7285759;220905305;YP_002480617.1;YP_002480617 |
| Ddes_2148 | Ddes_2148 | zinc/iron permease | 1 | 0 | -0.233 | 0 | 0.573 | PFAM: zinc/iron permease; KEGG: dvl:Dvul_2882 zinc/iron permease; zinc/iron permease | YP_002480722.1;Ddes_2148;Ddes_2148;7285864;220905410;YP_002480722.1;YP_002480722 |
| Ddes_2270 | Ddes_2270 | type 12 methyltransferase | 2 | 0 | 0.296 | 0 | 0.752 | PFAM: Methyltransferase type 12; KEGG: kra:Krad_2590 methyltransferase type 11; type 12 methyltransferase | YP_002480843.1;Ddes_2270;Ddes_2270;7285987;220905531;YP_002480843.1;YP_002480843 |
| Ddes_2346 | Ddes_2346 | hypothetical protein | 2 | 0 | -1.41 | 0 | 0.361 | hypothetical protein | YP_002480918.1;Ddes_2346;Ddes_2346;7286064;220905606;YP_002480918.1;YP_002480918 |
| Ddes_1207 | rpsA | 30S ribosomal protein S1 | 2 | 0 | 0.604 | 0 | -0.403 | in Escherichia coli this protein is involved in binding to the leader sequence of mRNAs and is itself bound to the 30S subunit; autoregulates expression via a C-terminal domain; in most gram negative organisms this protein is composed of 6 repeats of the S1 domain while in gram positive there are 4 repeats; the S1 nucleic acid-binding domain is found associated with other proteins; 30S ribosomal protein S1 | YP_002479789.1;rpsA;rpsA;Ddes_1207;7284889;220904477;YP_002479789.1;YP_002479789 |
| Ddes_1505 | thiH | thiamine biosynthesis protein ThiH | 2 | 0 | -0.224 | 0 | -0.462 | in Escherichia coli this enzyme functions in thiamine biosynthesis along with thiFSGI and IscS; with ThiFSG catalyzes the formation of thiazole phosphate from tyrosine, cysteine and 1-deoxy-D-xylulose-5-phosphate; forms a complex with ThiG; contains an iron-sulfur center; in Thermotoga this enzyme has an extra C-terminal domain; thiamine biosynthesis protein ThiH | YP_002480085.1;thiH;thiH;Ddes_1505;7285203;220904773;YP_002480085.1;YP_002480085 |

Supplementary Figure S1: Heatmap showing the inter-sample correlations (Pearson’s coefficient) between read numbers per gene. The correlation table was clustered by hierarchical clustering with complete linkage. Note the consistent grouping of samples according to the electron acceptor (Nitrate or Sulfate). Within the Sulfate cluster, samples SN1 and S1 are separated from the other ones and were therefore considered as outliers and discarded from the analysis of differential expression.


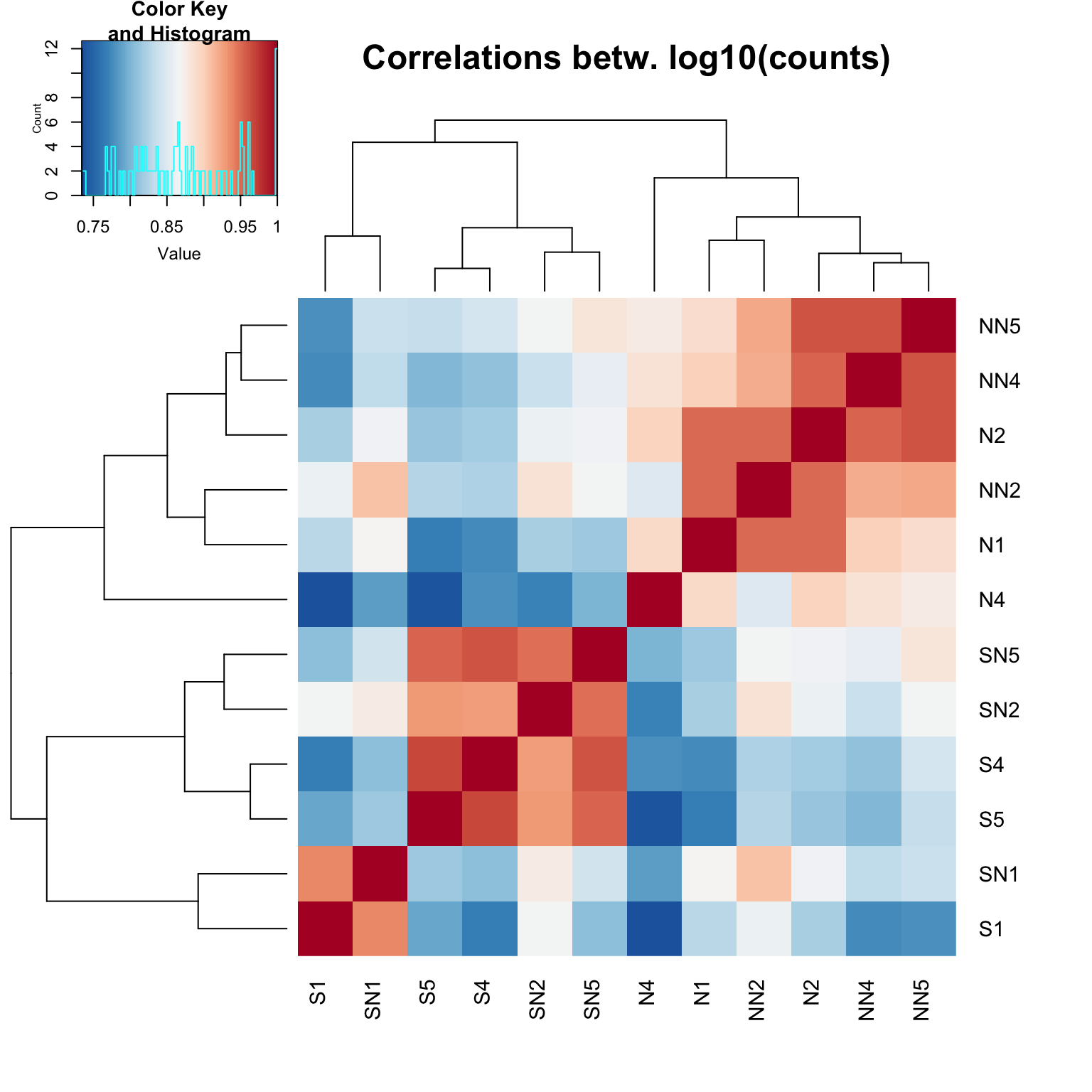


**Figure S2. Quantitative RT- PCR analysis of *hcpR1, hcp, nrfA, hcpR2* and *napC* genes in response to nitrate or NO**. (a) Differences in relative transcript abundances in response to growth in the presence of nitrate or sulfate. (b) Effect of nitric oxide treatment on transcript levels. *D. desulfuricans* were grown in the presence of sulfate and were either treated with nitric oxide saturated water or degassed water. Cells were harvested for total RNA purification. RNA was reverse transcribed and used for qPCR analysis using the ΔΔCt method. Transcript levels were normalised against *polA* levels and are presented relative to those of sulfate grown cells.

(a)


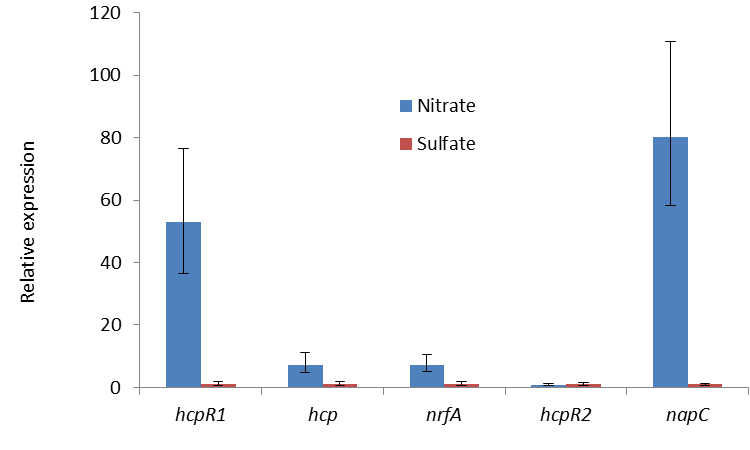


(b)


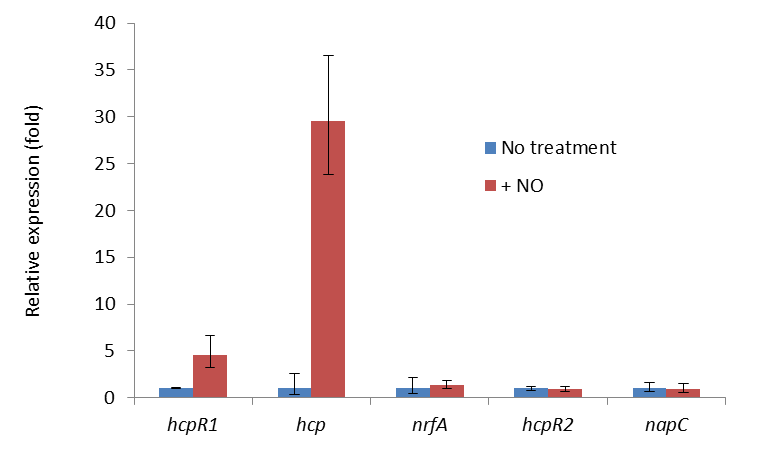


Figure S3. Development of a position specific scoring matrix for the bioinformatic prediction of the extent of the HcpR1 regulon

**Figure S4. Binding of HcpR1 to the *D. desulfuricans* Ddes_1825 and *sat* (Ddes_0454) promoter fragments.** DNA binding activities were measured by EMSA. ^32^P-labelled promoter DNA fragments were incubated with increasing concentrations of HcpR1 protein and then resolved by non-denaturing electrophoresis. Free DNA and HcpR-DNA complexes are labelled with arrows. Herring sperm DNA was included as a non-specific competitor. HcpR1 was included in the incubations at 0, 25, 50, 100 and 200 nM.


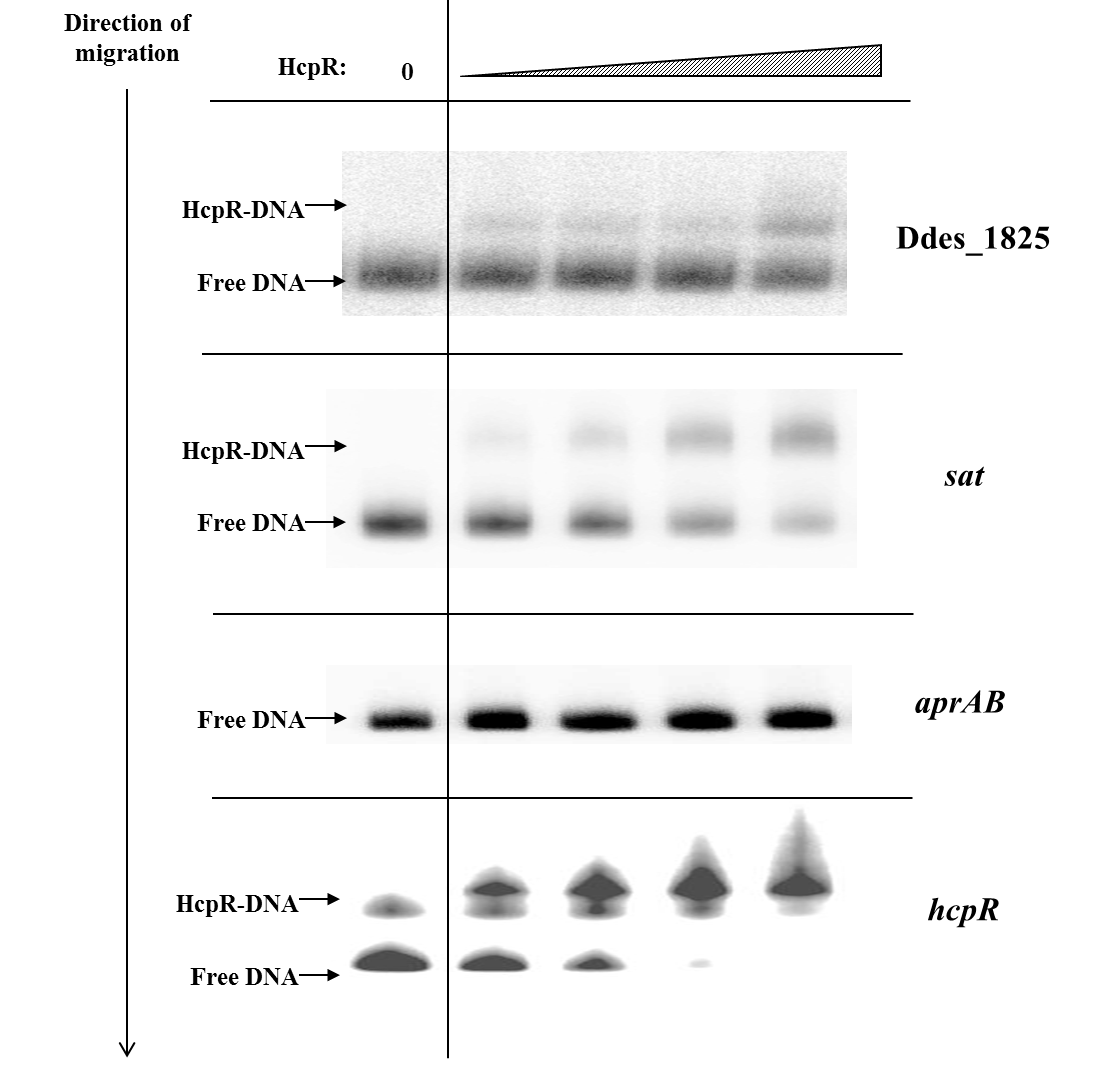


# Supplementary material

Technical report by Jacques van Helden

2017-10-02

Table of Contents

[DEG detected with all replicates or after discarting atypical samples 1](#__RefHeading___Toc495930907)

[References 4](#__RefHeading___Toc495930908)

## DEG detected with all replicates or after discarting atypical samples

Each experiment was led in 3 replicates. However, our exploration of the data clearly showed that two samples were atypical, since they clustered apart from all the other ones on the between-sample correlation heatmap. We thus decided to discard these the two following samples for the detection of differentially expressed genes:

- SN1: sulfate + NO stress
- S1: sulfate

To evaluate the impact of this decision on the detection of differentially expressed genes (**DEG**), we ran the analysis either with all the replicates (3 per condition) or without the atypical samples.

The Figure below compares the adjusted p-values returned by edgeR with all replicates (abcsissa) or after having discarded the atypical samples (ordinate).

For the three affected comparisons (sulfate + NO versus sulfate, nitrate versus sulfate, and notate + NO versus sulfate + NO) the results are consistently affected in the same way: the incorporation of the atypical samples increases all p-values, thereby resulting in a smaller number of genes passing the significance threshold (
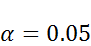
 on Padj).

In other terms, the impact of the atypical samples is to decrease the sensitivity of the DEG detection.

This result might seem contradictory with the detailed study of Schurch *et al.* (2016), who performed a detailed study of the impact of the number of replicates on the detection of differentially expressed genes in the yeast *Saccharomyces cerevisiae*, based on a sub-sampling of 48 samples per condition. As expected, their study clearly shows that the number of genes declared positives decreases when the number of samples is reduced (loss of sensitivity).

In contrast, in our case the inclusion of the atypical samples result in a reduction of the number of reported DEG. We interpret this loss of sensitivity as a consequence of an increase of the variance, since the atypical samples tend to increase the intra-group variance (variance between replicates of the same condition) for each gene. Since DEG detection always relies on an evaluation of the differences between conditions relative to the variance between replicates, and increase of this variance would provoke a loss of sensitivity.

Beyond this impact on the number of genes passing the significance threshold, Figure 1 shows a fairly good colinearity between the p-values obtained with and without the atypical replicates, respectively. Thus, our choice to discard the two atypical samples favours sensitivity, but does not completely perturbate the results.

Based on these observations, we considered that the inclusion of atypical samples would not bring additional information, but on the contrary it would blur out the relevant information, and we decided to discard them for the detection of differentially expressed genes.


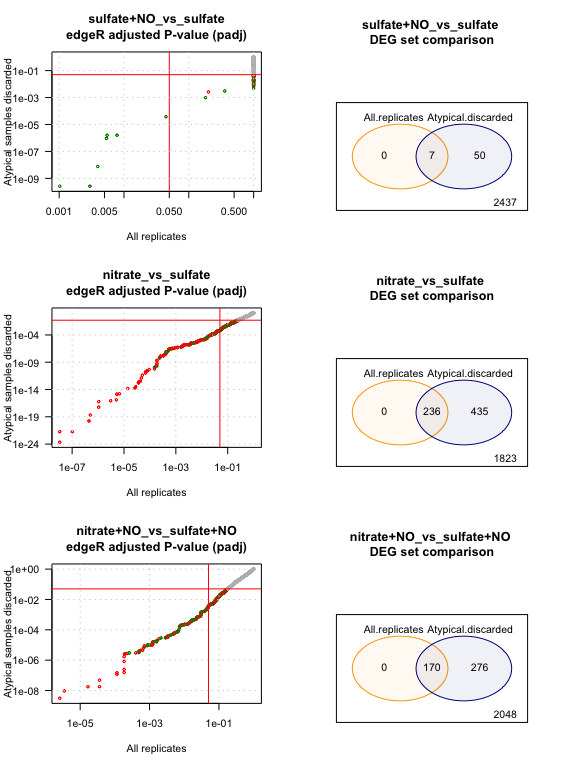


Impact of the removal of atypical samples on the detection of differentially expressed genes (DEG). Left panels: p-values obtained with all replicates (abscissa) or after discarding the atypical replicates (ordinate). THe red lines indicate the significance threshold (alpha=0.05). Right panels: Venn diagram comparing the number of DEG genes reported with all replicates or after having discarded the atypical samples, respectively.

## References

Schurch, N. J., P. Schofield, M. Gierli?ski, C. Cole, A. Sherstnev, V. Singh, N. Wrobel, et al. 2016. “How many biological replicates are needed in an RNA-seq experiment and which differential expression tool should you use?” *RNA* 22 (6): 839–51.
